# Anti-Inflammatory Activities of 8-Benzylaminoxanthines Showing High Adenosine A_2A_ and Dual A_1_/A_2A_ Receptor Affinity

**DOI:** 10.3390/ijms241813707

**Published:** 2023-09-05

**Authors:** Michał Załuski, Dorota Łażewska, Piotr Jaśko, Ewelina Honkisz-Orzechowska, Kamil J. Kuder, Andreas Brockmann, Gniewomir Latacz, Małgorzata Zygmunt, Maria Kaleta, Beril Anita Greser, Agnieszka Olejarz-Maciej, Magdalena Jastrzębska-Więsek, Christin Vielmuth, Christa E. Müller, Katarzyna Kieć-Kononowicz

**Affiliations:** 1 Department of Technology and Biotechnology of Drugs, Faculty of Pharmacy, Jagiellonian University Medical College in Kraków, Medyczna 9, 30-688 Krakow, Poland; zaluski.michal@gmail.com (M.Z.); ewelina.honkisz@uj.edu.pl (E.H.-O.); kamil.kuder@uj.edu.pl (K.J.K.); gniewomir.latacz@uj.edu.pl (G.L.); anita.greser@student.uj.edu.pl (B.A.G.); agnieszka.olejarz@uj.edu.pl (A.O.-M.); mfkonono@cyf-kr.edu.pl (K.K.-K.); 2 Department of Pharmaceutical & Medicinal Chemistry, Pharma Center Bonn & Pharmaceutical Institute, University of Bonn, An der Immenburg 4, 53121 Bonn, Germany; piotr.jasko@alumni.uj.edu.pl (P.J.); andi.brockmann@googlemail.com (A.B.); christin.vielmuth@uni-bonn.de (C.V.); christa.mueller@uni-bonn.de (C.E.M.); 3 Department of Pharmacodynamics, Jagiellonian University Medical College in Kraków, Medyczna 9, 30-688 Kraków, Poland; malgorzata.zygmunt@uj.edu.pl; 4 Department of Clinical Pharmacy, Jagiellonian University Medical College in Kraków, Medyczna 9, 30-688 Kraków, Poland; m.jastrzebska-wiesek@uj.edu.pl

**Keywords:** adenosine A_2A_ receptor, adenosine A_1_ receptor, xanthine derivatives, anti-inflammatory activity, Griess assay, phagocytic activity, molecular modelling, metabolic stability

## Abstract

Chronic inflammation plays an important role in the development of neurodegenerative diseases, such as Parkinson’s disease (PD). In the present study, we synthesized 25 novel xanthine derivatives with variable substituents at the *N1-*, *N3-* and C8-position as adenosine receptor antagonists with potential anti-inflammatory activity. The compounds were investigated in radioligand binding studies at all four human adenosine receptor subtypes, A_1_, A_2A_, A_2B_ and A_3_. Compounds showing nanomolar A_2A_ and dual A_1_/A_2A_ affinities were obtained. Three compounds, **19**, **22** and **24**, were selected for further studies. Docking and molecular dynamics simulation studies indicated binding poses and interactions within the orthosteric site of adenosine A_1_ and A_2A_ receptors. In vitro studies confirmed the high metabolic stability of the compounds, and the absence of toxicity at concentrations of up to 12.5 µM in various cell lines (SH-SY5Y, HepG2 and BV2). Compounds **19** and **22** showed anti-inflammatory activity in vitro. In vivo studies in mice investigating carrageenan- and formalin-induced inflammation identified compound **24** as the most potent anti-inflammatory derivative. Future studies are warranted to further optimize the compounds and to explore their therapeutic potential in neurodegenerative diseases.

## 1. Introduction

Parkinson’s disease is a progressive neurodegenerative disorder characterized by motor and non-motor symptoms. It is prevalent in older people, but in certain cases, younger patients can be affected [1]. The pathogenesis of Parkinson’s disease is related to the degeneration of dopaminergic neurons in the cortical part of the black matter, as well as the accumulation of Lewy bodies, i.e., abnormal aggregates containing proteins such as α-synuclein, which disrupts normal cell function [1,2]. Signs of PD are usually observed when approximately 60–70% of dopaminergic neurones have been destroyed [1]. However, the appearance of the first pathological changes can precede the onset of visible symptoms by up to 20 years. Increasing evidence suggests that the development of PD begins in the gut, and from there spreads to the brain via the vagus nerve [2]. In recent years, much attention has also been paid to the role of inflammatory factors, particularly the ongoing inflammatory processes in the nervous system (neuroinflammation) playing an important role in the pathogenesis of PD. Neuroinflammation is a process that occurs at the molecular and cellular levels, and results from interactions between different factors. In PD, it especially concerns cell types in the brain that are sensitive to α-synuclein aggregates, i.e., neurones, astrocytes, microglia or endothelial cells. This leads to an impairment of their function and increases the secretion of pro-inflammatory cytokines (such as IL6, IL1, TNF and IFN) and chemokines (e.g., CCL2 and CXCL1). In addition, peripheral immune cells (such as CD4^+^ T lymphocytes) enter the brain. All this causes an increase in the pro-inflammatory environment, i.e., activation of microglia, production of toxic factors or pro-inflammatory cytokines and chemokines, and together with oxidative stress, this results in neuroinflammation and consequently neurodegeneration [3]. In fact, some studies have shown a genetic similarity between patients with PD and other autoimmune and inflammatory diseases [4]. Moreover, with age, the normal function of the immune system declines, the number of naive T and B lymphocytes decreases and the accumulation of memory cells (T and B) increases, leading to an increased sensitivity to infection and the formation of autoantibodies [4]. 

The main treatment strategy for PD is based on increasing dopaminergic transmission. Increases in dopamine levels are primarily achieved using its precursor levodopa together with drugs that inhibit its metabolism as aromatic amino acid decarboxylase inhibitors (in the periphery: carbidopa, benserazide), catechol-*O*-methyltransferase inhibitors (tolcapone, entacapone, opicapone) or monoamine oxidase B inhibitors (selegiline, rasagiline, safinamide). However, long-term use of levodopa leads to a decrease in its efficacy and duration of action. Therefore, drugs with other targets and mechanisms of action, such as amantadine (NMDA antagonist) or istradefylline (adenosine A_2A_ receptor antagonist), are applied in combination therapies [5]. 

**Figure 1:**
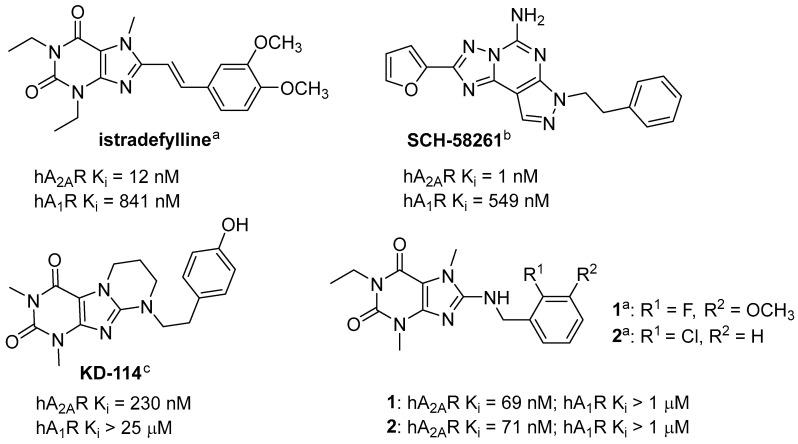
Structures of adenosine A_2A_ receptor antagonists; h = human; ^a^ information from [13]; ^b^ information from [9]; ^c^ information from [12].

Istradefylline (Figure 1) is an adenosine A_2A_ receptor (A_2A_R) antagonist with a xanthine core structure, which has been approved for therapeutic use in Japan (2013) and in the USA (2019) [6]. The A_2A_R belongs to the adenosine receptor family, which includes three further receptor subtypes: A_1_, A_2B_ and A_3_. At all of them, the endogenous nucleoside adenosine acts as the cognate agonist, while xanthine derivatives act as antagonists [7]. Under physiological conditions, adenosine levels are low, but increase as a result of hypoxia, tissue damage and inflammation [8]. In the body, adenosine can have a strong effect on inflammation.

Animal studies applying an A_2A_R antagonist (**SCH-58261** [9]; Figure 1) have shown its beneficial effects in a model of inflammation induced by lipopolysaccharide (LPS) administration [10]. This compound inhibited the increase in p38 and JNK phosphorylation and caspase 3 activation. In addition, it stopped activated microglial recruitment and the increase in the level of interleukin 1ß (IL-1ß) [10]. A similar beneficial effect of this compound was also observed in both in vivo and in vitro studies in two models of perinatal brain injury: the low-protein diet (LPD) model (chronic) and ibotenate model (acute) [11]. In both of these models, an increase in A_2A_R expression in microglia cells as well as in pro-inflammatory factors (IL-1ß, IL-6, inducible nitric oxide synthase and tumor necrosis factor alpha) was observed. The application of **SCH-58261** caused a decrease in the above-mentioned cytokines, whereas treatment with the A_2A_R agonist (CGS-21680) showed the opposite effect.

Our previous studies with A_2A_R antagonists (xanthine derivatives) showed the anti-inflammatory activity of tested compounds, **KD-114** and compound **1** (Figure 1), in carrageenan-induced edema in mice and rats, respectively [12,13]. **KD-114**, tested at a dose of 100 mg/kg *intraperitoneally* (*ip*), significantly (>90%) reduced edema volume in mice at the tested time points (1st, 2nd and 3rd hour). An especially strong effect was observed at the first hour with a reduction from 64% (control) to 2% (with the tested compound) [12]. For compound **1** tested at a dose of 20 mg/kg *ip*, the strongest inhibition of edema in rats was observed at the 2nd and 3rd hour (44% and 53%, respectively) [13]. Both compounds display good affinity for the A_2A_R (K_i_ = 230 nM, **KD-114**; K_i_ = 69 nM, **1**) and selectivity towards other adenosine receptors (K_i_ > 1 μM). 

**Figure 2:**
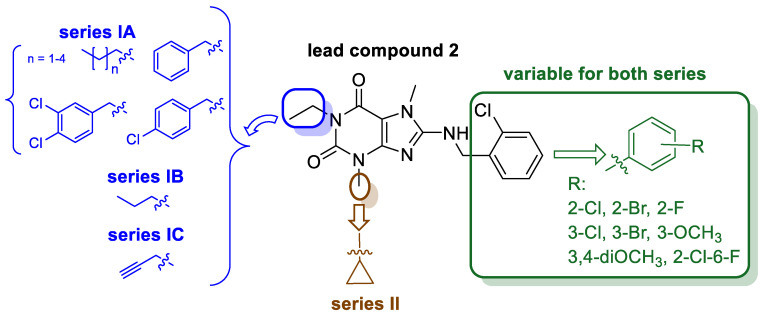
Lead compound **2** and structural modifications.

The aim of this work was to synthesize novel substituted xanthine derivatives, mainly with a 3,7-dimethylxanthine core, and to evaluate their affinity for adenosine receptors. Further pharmacological studies, both in vitro and in vivo, were then planned for selected derivatives. Compound **2** (Figure 1) from our previous publication was chosen as a lead structure for the design of new compounds [13]. This compound was characterized by a high affinity for A_2A_R (K_i_ = 71 nM) and the lack of significant interactions with other adenosine receptors (K_i_ > 1000 nM), similar to compound **1**. However, compound **2** was chosen for economic reasons as an alternative. The planned structural modifications are shown in Figure 2.

Two series of compounds were designed: Series **I** (blue—a variable substituent in the *N1* position) and series **II** (brown—a cyclopropyl substituent in the *N3* position). In series **I**, three groups of compounds were planned: series **IA**—a divergent substituent in the *N1* position; **IB**—a propyl substituent; **IC**—a propargyl substituent. In each of these series (I and II), the substituents in the benzylamine ring (green color) were changed as shown in Figure 2.

## 2. Results and Discussion

### 2.1. Synthesis of Target Compounds

**Scheme 1:**
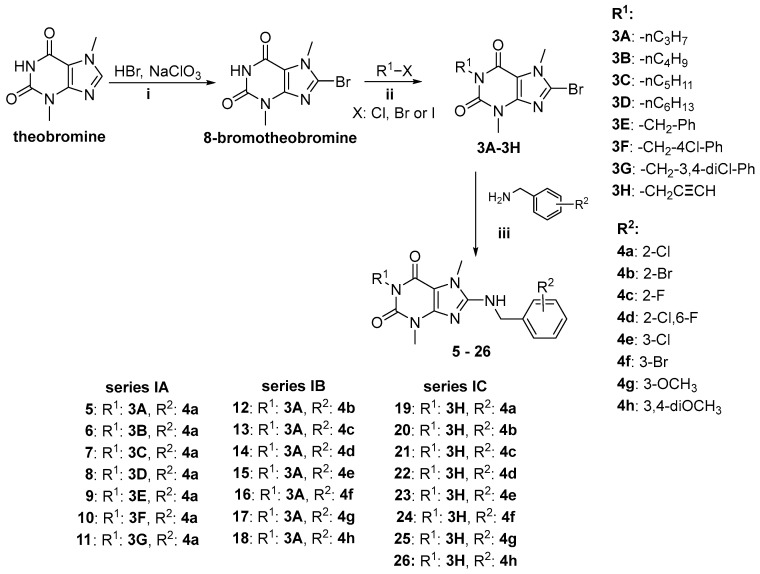
Synthesis of compounds **5**–**26**. Reagents and conditions: (**i**) NaClO_3_, CH_3_COOH, H_2_O, 80 °C, 2 h; (**ii**) K_2_CO_3_, DMF, 75 °C, 4 h; (**iii**) triethylamine (TEA), n−C_3_H_7_OH, 150 °C, microwave irradiation (300 W; 10 bar), 1 h or CH_3_OCH_2_OH, reflux 15–17 h.

**Scheme 2:**
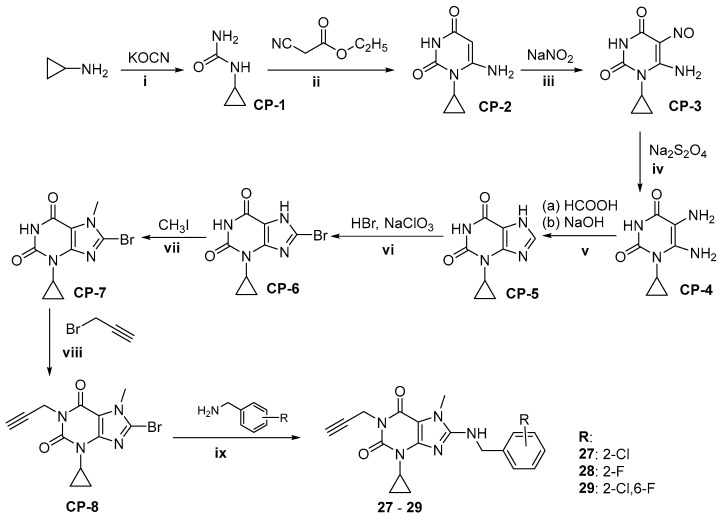
Synthetic route of compounds **27**–**29**. Reagents and conditions: (**i**) 5 N HCl, 70 °C, 4 h; (**ii**) CH_3_CH_2_ONa, 100 °C, 4 h; (**iii**) 50% CH_3_COOH, 60 °C; (**iv**) 12.5% NH_3_, 70 °C; (**v**) (a) reflux 1 h, (b) reflux 2 h; (**vi**) CH_3_COOH, first 58 °C, then: reflux 2 h; (**vii**) DIPEA, DMF, 40 °C, 4 h; (**viii**) K_2_CO_3_, DMF, 75 °C, 4 h; (**ix**) TEA, n−C_3_H_7_OH, 150 °C, microwave irradiation (300 W; 10 bar), 1 h.

The synthetic routes are shown in Scheme 1 and Scheme 2. Compounds were obtained according to the methods previously described [13]. For most compounds (Scheme 1), the starting substrate for the synthesis was theobromine, which was first oxidatively brominated at position C8, followed by *N*-alkylation at position 1 and finally followed by a nucleophilic substitution with benzylamine derivatives. Reactions with benzylamines were carried out either in a microwave oven or by traditional heating. All compounds of **series I** (**5**–**26**) were obtained in this way. The yields of the reactions carried out using the microwave oven were higher, ranging from 39–72%, whereas under traditional conditions, only 4–28% were obtained (except for product **9**: 50%).

For compounds **27**–**29**, the starting material was cyclopropylamine, which, through a series of reactions shown in Scheme 2, provided 1-cyclopropylxanthine (**CP-5**). Then, compound **CP-5** was sequentially brominated with hydrobromide in the presence of sodium chlorate at position C8 (**CP-6**), *N*-methylated with methyl iodide at position *N7* (**CP-7**), *N*-alkylated with propargyl bromide at position *N1* (**CP-8**) and finally condensed with benzylamines. The final reaction was carried out using a microwave oven with good yields (44–48%).

The structures of the synthesized compounds were confirmed by spectral analysis including ^1^H NMR, ^13^C NMR and LC-MS (spectra are provided in Supplementary Materials). Melting points were determined for all new compounds, and the purity of the compounds was determined to be at least 95% using ultra-performance liquid chromatography—mass spectrometry (UPLC-MS) (except **27**: 93.19%).

### 2.2. Pharmacological Activity In Vitro

#### 2.2.1. Structure-Activity Relationships at Adenosine Receptors

Affinities of the two series of 8-benzylaminoxanthine derivatives at human adenosine receptor subtypes were determined in radioligand binding studies (see Table 1). All modifications introduced (i.e., type of substituent at the *N1*, *N3* and C8 positions) affected affinities. All compounds (except five: **7**, **8**, **10**, **11** and **13**) showed affinities for adenosine, the A_2A_R in the nanomolar range (60 nM < K_i_ < 800 nM), whereas only ten compounds (**16**, **19**, **21**–**25**, **27**–**29**) displayed affinities for the A_1_R in the nanomolar range (70 nM < K_i_ < 470 nM). None of the compounds had significant affinities for the A_3_R (K_i_ > 1 µM), while only two compounds showed moderate affinities for the A_2B_R (**5**: K_i_ = 832 nM; **14**: K_i_ = 687 nM).

Although all of the introduced modifications affected binding affinity, the strongest effect was observed for substituents in the *N1*-position, and this was observed at both A_1_R and A_2A_R. This influence is particularly evident in compounds with a 2-chlorobenzylamine substituent in the C8-position (compounds **5**–**11**, **19**). An elongation of the alkyl chain from ethyl (lead **2**; K_i_ = 71 nM) to propyl (**5**; K_i_ = 302, nM), butyl (**6**; K_i_ = 318 nM), pentyl (**7**; K_i_ > 1000 nM) or hexyl (**8**; K_i_ > 1000 nM) led to a decrease in affinity for A_2A_R. A benzyl substituent at the *N1*-position (**9**; K_i_ = 323 nM) also caused a similar decrease in affinity for the A_2A_R as the propyl (**5**) or butyl (**6**) substituent. In contrast, the introduction of chlorine atom(s) into the benzyl ring (**10** and **11**: K_i_ > 1000 nM) resulted in a lack of affinity. In contrast, the introduced propargyl substituent at *N1* (**19**; K_i_ = 96 nM) induced a small decrease in affinity for A_2A_R compared to compound **2**, but contributed to good affinity for A_1_R (**19**: K_i_ = 407 nM). This beneficial effect of the propargyl group was observed throughout the **series IC**, with all compounds showing good affinities for A_2A_R (60 nM < K_i_ < 300 nM) and good affinities for A_1_R (70 < K_i_ < 470 nM) with the exception of compounds **20** and **26** (A_1_R K_i_ > 1000 nM). In this series, the type and position of the substituent in the benzylamine ring determined the affinity for these receptors. In this group, there are usually compounds with higher affinities for A_2A_R than A_1_R, but there is also compound **24** with a similar effect on both receptors (A_2A_R: K_i_ = 77 nM; A_1_R: K_i_ = 72 nM). This compound (**24**) and compound **22** (A_2A_R: K_i_ = 62 nM; A_1_R: K_i_ = 130 nM) showed the highest affinities for A_2A_R among all synthesized compounds. 

The introduction of a cyclopropyl moiety at the *N*3-position (compounds **27**–**29**) caused a change in the affinity profile: an increased affinity for A_1_R and a slightly decreased affinity for A_2A_R (compare **19** vs. **27**; **21** vs. **28** and **22** vs. **29**). In this group is compound **29** with a higher affinity for A_1_R than A_2A_R (A_1_R: K_i_ = 78 nM, A_2A_R: K_i_ = 204 nM).

#### 2.2.2. Human Monoamine Oxidase B (MAO B) Inhibition

The inhibitory activity of selected compounds for human MAO B (hMAO B) was determined by a spectrofluorometric method [13]. For these studies, compounds with propyl (**5**, **12**–**18**) and propargyl (**19**–**26**) moieties were tested at a concentration of 1 µM. None of them showed hMAO B inhibition of >50% at this concentration, therefore, no IC_50_ values were determined (Table 2). Generally, the percent of hMAO B inhibition was lower than 20% (except for **20**, 32%).

### 2.3. Molecular Docking to Adenosine A_1_ and A_2A_ Receptors and Molecular Dynamics (MD) Simulations

For docking studies, we selected receptor structures 5N2S and 5N2R [14], solved with the xanthine derivative PSB-36 [15], which shares common structural elements with the series of herein studied ligands: substitution with an alkyl at the *N*1 and *N*3 positions, and a spatially large substituent at the C8 position. For both of the biological targets tested, the ligands showed a number of interactions overlapping with those previously described in other resolved structures. Furthermore, in both cases, the so-called ionic-lock between R^3.50^ and E^6.30^ residues was maintained, which is typical for the inactive state of the receptors.

#### 2.3.1. Docking to the 5N2S (Adenosine A_1_ Receptor) and MD Simulations

**Figure 3:**
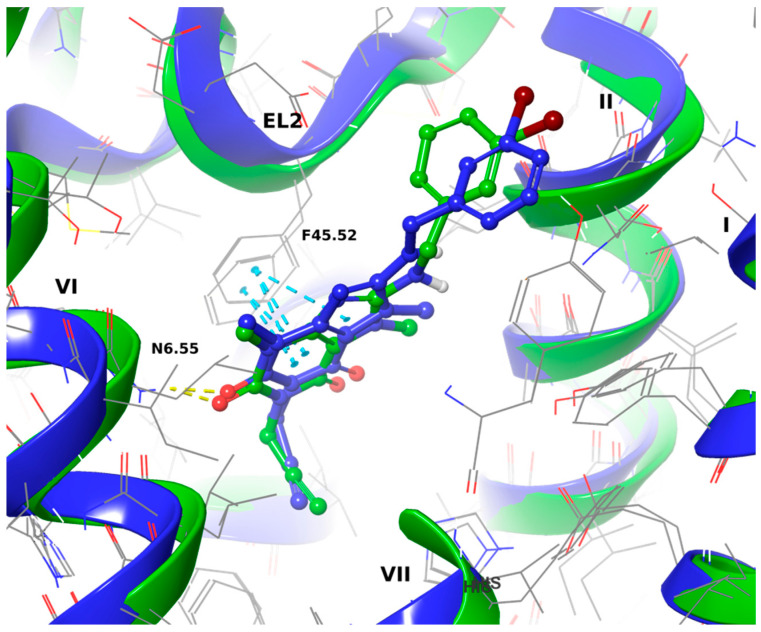
Putative binding mode of **24** in 5N2S (green ribbons) and 5N2R (blue ribbons) complexes. Yellow dashed lines depict H-bonds, blue π-π interaction, Roman numbers denote TM helices. TM7 was partially removed from both structures to increase figure visibility.

Three complexes were chosen for the estimation of stability, with MM-GBSA values [kcal/mol] of **19** (**MZ1483**): −79.01, **22** (**MZ1490**): −77.77 and **24** (**MZ1495**): −75.26. All three complexes showed similar, yet nonclassical, binding poses. The propargyl fragments are likely enclosed in a TM6 cage formed by L^6.51^, H^6.52^ on the sides and W^6.48^. Due to *N*7- and *N*3-methyl substitution, the ligands are expected to be placed in a rather parallel orientation with the membrane, and a key hydrogen bond may be formed between N^6.55^ and the C2 carbonyl oxygen. The xanthine ring is likely stabilized by π-π interactions with F^45.52^, and the chlorine atom of **19** may form a halogen bond with N^2.65^. During a 150-ns simulation, **19** remained stable from the ~20th ns of the trajectory, yet displayed additional interactions with the aminoalkyl group and H^7.43^ most of the time during the simulation. However, it did not display additional contacts, such as those seen with **24**, where K265 interacted through water with *N9*, and the distal substituted benzene ring was stabilized by Y^7.36^, which could explain its higher affinity to A_1A_R when compared to other tested ligands, despite the slight translation in the binding pocket at the beginning of the simulation. Compound **22**, on the other hand, lost the interaction with key N^6.55^ at ~20 ns of the recorded trajectory and was mainly stabilized by hydrophobic interactions with F171(^45.52^) and Y^7.36^ or I^7.39^. The comparison of the putative binding modes for **24** in both proteins is depicted in Figure 3.

#### 2.3.2. Docking to 5N2R (Adenosine A_2A_ Receptor) and MD Simulations

**Figure 4:**
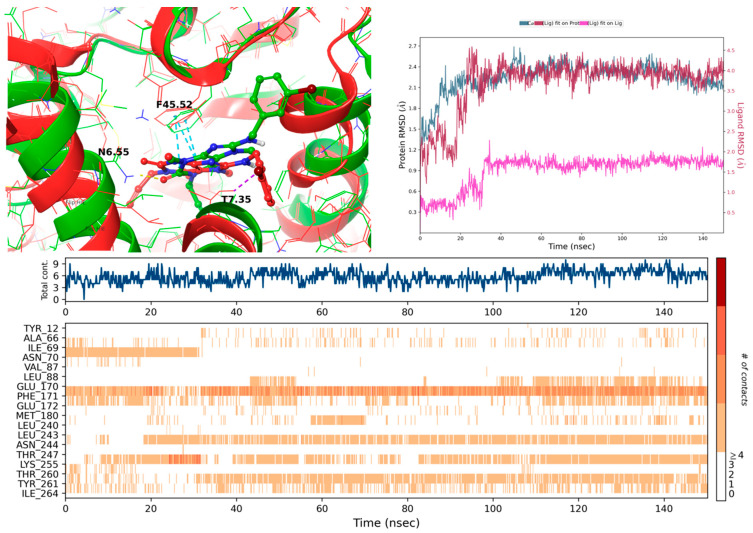
Top left panel—alignment of 1st (green) and 1000th (red) frame of **24**-5N2S complex trajectory. TM7 and EL3 were partially removed for better image visibility; top right panel RMSD evolution of a protein (left Y-axis) and ligand (right Y-axis; pink—Fit on ligand, maroon—Fit on protein). Bottom panel: Protein-ligand contacts timeline; the darker the color, the more interactions recorded.

Selected complexes displayed MM-GBSA values [kcal/mol] of −63.22 (**19**), −71.26 (**22**) and −64.76 (**24**). Values correlate with their biological affinity values. The orientation of the ligands in the 5N2R structure was similar to that of 5N2S. A key hydrogen bond likely exists between N^6.55^ and the carbonyl oxygen C2, and the xanthine core may be stabilized through π-π interactions with F^45.52^. An additional interaction of the alkyl linker amine group forming H-bond with E^45.53^ was also found. However, due to structural differences between the two receptor subtypes, the propargyl moiety appeared to interact with V^3.32^, L^6.51^ and W^6.48^. However, during the simulations, the ligands changed their positions in the binding pocket at the beginning of the recorded trajectories. In the case of **19** in the 20th ns of the simulation, the ligand moved toward N^6.55^ and retained the formed H-bond with *N9* through most of the remaining simulation time and occupied the very position until the end of the simulation. A similar shift of the ligand in the binding pocket was observed for the **22**/5N2R complex in ca. the 15th ns of the trajectory, so that the C2 carbonyl oxygen interacted through most of the simulation with H^6.52^ and the *N*9 formed a water-bridged H bond with N^6.55^. The least stable interactions through the recorded trajectory were found for **24**, mostly stabilized through π-π stacking with F^45.52^ and an H bond between the alkyl NH group and S^2.65^, lost when the substituted benzene ring targeted EL3, in the second half of the simulation performed. A detailed RMSD and RMSE, contact timeline and histogram for all recorded trajectories (including *apo* proteins) can be found in Supplementary Data (Figures S1–S3). Figure 4 shows the exemplary behavior of the **24**/5N2S complex in the recorded trajectory. 

### 2.4. Selected ADMET Properties—Evaluation In Vitro

Based on our results of the binding affinities (Table 1) three compounds, namely the most promising dual A_1_R/A_2A_R ligands, were selected for further studies: **19** (four times more potent as A_2A_R ligand), **22** (twice more potent as A_2A_R ligand) and **24** (with an equal potency for both receptors). 

#### 2.4.1. Toxicity Evaluation

**Figure 5:**
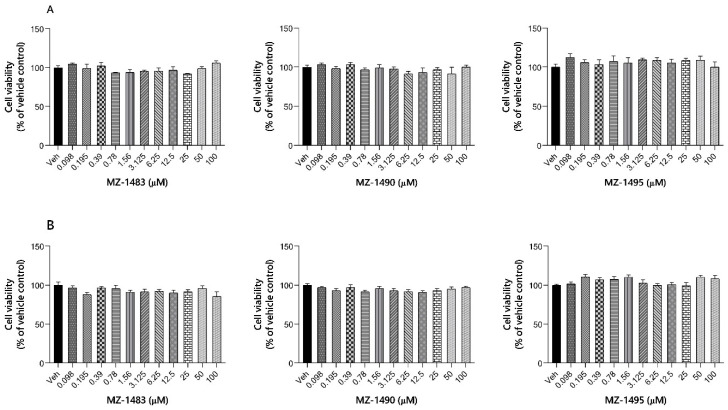
Toxicity evaluation of compounds **19** (**MZ-1483**), **22** (**MZ-1490**) and **24** (**MZ-1495**) in HepG2 cells (**A**) and SH-SY5Y cells (**B**). The graphs were generated using GraphPad Prism software 8.0. Each point represents the mean ± SEM of three independent experiments, each of which consisted of three replicates per treatment group. Statistical significance was evaluated by one-way ANOVA with post hoc Dunnett test at significance level α = 0.05 and *p*-values are detailed in the Figure.

Drug safety is a critical step in the process of drug development. It is important to ensure that these drugs do not cause adverse effects on important organ systems such as the central nervous system (CNS) or the liver. In our studies, neurotoxicity and hepatotoxicity were evaluated in SH-SY5Y and HepG2 cells, respectively. The compounds were tested at 11 concentrations from 0.098 μM to 100 μM (see Figure 5). Cell viability was assessed after incubation for 24 h with the SH-SY5Y line and for 48 h with the HepG2 line. 

Dose-response analysis showed that compounds **19** (**MZ-1483**), **22** (**MZ-1490**) and **24** (**MZ-1495**) displayed a high safety profile in these cell lines (Figure 5). An examination of cultures under an inverted microscope during incubation with the tested compounds revealed that, at the highest concentrations (25 μM, 50 μM, 100 μM), the compounds precipitated and formed crystals in the culture medium.

#### 2.4.2. Metabolic Stability

Metabolic stability is related to the ability of a compound to undergo biotransformation. As most therapeutic compounds undergo biotransformation in liver tissue, liver microsomes of various species, e.g., human liver microsomes (HLMs), are commonly used for preliminary in vitro assessment of metabolic stability [16]. 

In our study, the compounds were incubated for 120 min with HLMs, followed by liquid chromatography-mass spectrometry (LC-MS) to identify the formed metabolites. The tested compounds were found to be metabolically stable compared to the unstable reference drug verapamil. The reaction mixtures after incubation showed a similar % remaining for all substrates, which was more than 90% (Table 3, Figures S4–S6 in Supplementary Materials). The same calculated value for the unstable drug verapamil was only 31%, after incubation with HLMs under the same conditions (Table 3; Figure S11). Furthermore, **19** formed two metabolites, while **22** and **24** only formed one metabolite. Moreover, according to the MS data (*m*/*z*~249) and retention times (t~6.26 s) in UPLC chromatograms, all compounds were most likely metabolized in the same way (Table 3, Figures S4–S9 in Supplementary Materials). The most probable metabolic pathways for all compounds were proposed with the support of MetaSite 6.0.1 software (Figure S4 in Supplementary Materials), such as fragmentation and hydroxylation. Results are shown in Figures S4, S6, S8 and S10 in Supplementary Materials.

#### 2.4.3. Potential for Drug-Drug Interactions

**Figure 6:**
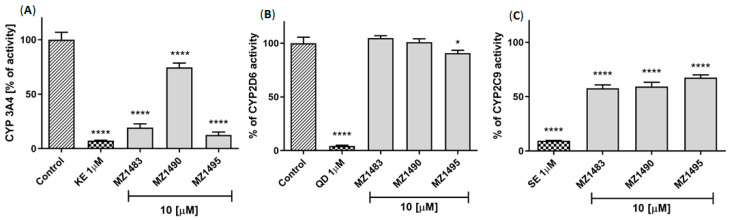
The effect of compounds **19** (**MZ-1483**), **22** (**MZ-1490**) and **24** (**MZ-1495**) on the activity of recombinant human cytochromes. (**A**): The influence on CYP3A4 activity. Statistical significance was evaluated by one-way ANOVA, followed by Bonferroni’s comparison test (**** *p* < 0.001). KE—Ketoconazole. (**B**): The influence on CYP2D6 activity. Statistical significance was evaluated by one-way ANOVA, followed by Bonferroni’s comparison test (* *p* < 0.05, **** *p* < 0.001). QD—Quinidine. (**C**): The influence on CYP2C9 activity. Statistical significance was evaluated by one-way ANOVA, followed by Bonferroni’s comparison test (**** *p* < 0.001). SE—Sulfaphenazole.

To predict potential drug-drug interactions (DDI) effects on three of the most relevant cytochrome P450 (CYP), isoforms CYP3A4, CYP2D6 and CYP2C9 were investigated at a concentration of 10 µM. The results were compared with the effects of 1 µM of the selective inhibitors ketoconazole (CYP3A4), quinidine (CYP2D6) and sulfaphenazole (CYP2C9). Compound **22** (**MZ1490**) showed the smallest effect on CYP3A4 activity (around 75% of the control activity), while **19** (**MZ1483**) and **24** (**MZ1495**) were found to be strong CYP3A4 inhibitors, decreasing its activity to ~20% at 10 µM (Figure 6A). Regarding CYP2D6, a slight, statistically significant inhibition was only observed for **24** (Figure 6B). All compounds similarly inhibited the activity of CYP2C9 to around 65–70% of control activity (Figure 6C).

#### 2.4.4. Blood Brain Barrier Permeability

The ability of compounds, especially those intended to act in the CNS, to penetrate the blood-brain barrier (BBB) is an important property to consider in the initial stages of drug candidate screening. The PAMPA assay is a commonly used test to assess the passive permeation of the BBB [17]. In our study, caffeine was used as a highly permeable compound. The tested compounds (**19**, **22** and **24**) were dissolved in phosphate-buffered saline (PBS) (100 μM); due to precipitation, the solutions were sonicated for 20 min before adding them to the donor plate. After 5 h of incubation at room temperature, the results were analyzed by LC-MS, and the permeability coefficients (*P_e_*) were calculated. The results (Table 4) showed that only one compound, **19**, could penetrate the BBB as indicated by a calculated *P_e_* parameter of greater than 1.5 × 10^−6^ cm/s. This value is the manufacturer’s stated breakpoint for permeable compounds. For compounds **22** and **24**, the values were much lower than 1.5 × 10^−6^ cm/s (0.37 and 0.89 × 10^−6^ cm/s, respectively). This is puzzling, as these compounds do not structurally differ that much from each other, nor are there any significant differences in the calculated lipophilicity parameter logP (2.19, 2.35, 2.46, respectively, calculated in ChemDraw). It is possible that the value of this parameter may have been influenced by their poor solubility in PBS and, although sonication improved it, it may not have been the same in every case, although the differences were not visually apparent. 

### 2.5. Anti-Inflammatory Activity In Vitro

#### 2.5.1. Preliminary Screening of Anti-Inflammatory Activity in Griess Assay

**Figure 7:**
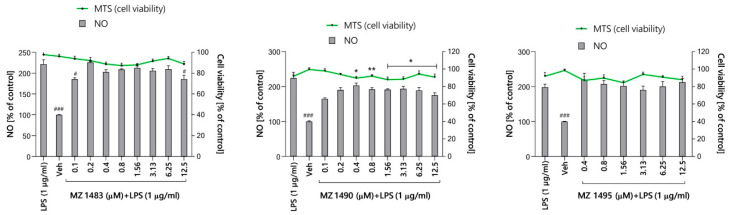
The effect of compounds **19** (**MZ-1483**), **22** (**MZ-1490**) and **24** (**MZ-1495**) on nitric oxide (NO) production in BV-2 cells assessed by the Griess assay (column graph) and cell viability assessed by MTS assay (line graph). Data are expressed as mean ± SEM of three independent experiments, each of which consisted of three replicates per treatment group and is expressed as a percentage of NO production versus vehicle cells (untreated cells) set at 100%. Statistical analysis by one-way ANOVA showed significant differences between the groups (α = 0.05) and was followed by Dunnett’s multiple comparison test. Data indicated with * *p* = 0.033; ** *p* = 0.002 reflect statistically significant differences between the control and experimental groups. Data indicated with ^#^ *p* = 0.033; ^###^ *p* < 0.001 reflect statistically significant differences between LPS = treated cells and experimental groups.

There are several types of immune cells that synthesize nitric oxide (NO), which is a signaling molecule in response to inflammation [18]. NO plays an important role in the pathogenesis of inflammation. To assess the ability of compounds to inhibit NO production, the Griess assay was applied. This is a colorimetric assay to measure the levels of nitrite (NO_2_^−^), which is one of the primary and stable metabolites of NO. The anti-inflammatory activity of compounds was examined in BV-2 cells treated with LPS as previously described [19]. Briefly, BV-2 cells were first pre-treated with increasing concentrations of tested compounds for 1 h, followed by LPS (1 μg/mL) stimulation for 24 h. Together with the Griess assay, the 3-(4,5-dimethylthiazol-2-yl)-5-(3-carboxymethoxyphenyl)-2-(4-sulfophenyl)-2H-tetrazolium (MTS) assay was performed to ensure that the decrease in NO level was not an effect of toxicity. No significant toxic effect of the compounds tested on BV2 cell viability was observed during the experiment. The results showed that only **19** (**MZ-1483**) and **22** (**MZ-1490**) showed moderate anti-inflammatory activity at the lowest concentrations (**19**: 100 nM and **22**: 100 and 200 nM) (Figure 7). While in the presence of LPS, the amount of NO produced increased by 140% as compared to the control cells; in the presence of the tested compounds, NO levels were only 60% to 90% higher than in the control cells. 

#### 2.5.2. Phagocytic Activity

Phagocytosis is a cellular process that allows a cell to properly function by removing harmful agents from it. Microglia, as resident macrophages of the CNS, naturally participate in phagocytosis during neural development, maintaining homeostasis, and diseased states [20]. It has not been clearly established which phagocytic phenotype (less or more active) is beneficial or deleterious for neurodegenerative disease progression. In PD, when α-synuclein aggregates are formed, both autophagy and microglia phagocytosis are impaired, affecting the degradation and clearance of α-synuclein. This dysfunction of autophagy and phagocytosis leads to predominantly M1-type (pro-inflammatory) microglia and the release of pro-inflammatory cytokines (IL-1ß, IL-6 and IL-12). This, in turn, contributes to the transformation of M2-type microglial cells into M1-type cells; i.e., an intensification of the entire inflammatory process [21]. 

**Figure 8:**
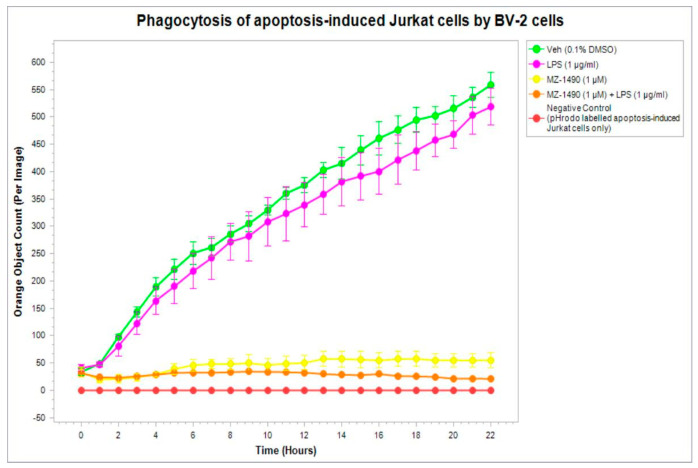
Real-time efferocytosis activity of BV−2 cells. The graph shows the kinetic curve of phagocytosis of pH−rodo labeled apoptotic Jurkat cells.

For the evaluation of phagocytic activity, compound **22** (**MZ1490**) was chosen as the most promising one on the basis of the results from the Griess assay. This compound was tested at a concentration of 1 µM, and phagocytosis was monitored in real time using an IncuCyte^®^ pHrodo^®^ Red Cell Labeling Kit (catalog no. 4766, Sartorius, Göttingen, Germany). IncuCyte analysis showed that BV-2 cells without or after treatment with LPS have a high phagocytic potential, which is in line with previous findings [22]. It is interesting to note that the phagocytic activity exerted by untreated BV-2 cells is also related to the serum concentration. A healthy CNS maintains the BBB, and microglia are often highly ramified surveillance microglia without enhanced phagocytosis [23]. However, in pathological states of the CNS, such as neurodegenerative diseases, the BBB is compromised, resulting in vascular leaks. This is a valuable finding in understanding the behavior of microglia. In our experimental conditions, BV-2 cells were cultured in the presence of 10% fetal bovine serum. Thus, it mimics a pathological state in which BV-2 cells have the morphology of fully activated microglia typically found in the injured brain [24]. An interesting finding is that **22** protects microglia from LPS-induced inflammation and, therefore, also from phagocytosis (Figure 8). Similar results were obtained, where BV-2 cells were treated with **22** alone.

### 2.6. Antinociceptive and Antiinflammatory Activity In Vivo

The positive results of the anti-inflammatory in vitro studies encouraged us to carry out preliminary in vivo studies to confirm the activity of tested compounds observed in vitro. For this purpose, a model with inflammation induction was used by the administration of carrageenan. This model has been used for years to evaluate the anti-inflammatory effects of compounds [25,26]. Furthermore, analgesic and anti-inflammatory activities were carried out in a formalin test [27].

#### 2.6.1. Anti-Inflammatory (Antiedematous) Effect in the Carrageenan-Induced Edema Model

The carrageenan test is used to evaluate the anti-inflammatory effect, as the injection of this compound into the hind paw of an animal induces long-lasting edema. The aim of the present study was to evaluate the anti-inflammatory (antiedematous) activity of the compounds tested: **19**, **22** and **24**. The compounds were administered at a dose of 20 mg/kg of body weight (bw). Ketoprofen, used as a reference compound and administered *ip* at a dose of 20 mg kg bw, inhibited edema formation by 60%, 63.3% and 45.1% in three consecutive hours of the experiment, respectively (Table 5). All tested compounds decreased the volume of edema induced by *subcutaneous* carrageenan injection into the hind paw of rats (Table 5). The strongest anti-inflammatory effect in this assay was produced by **24**. A statistically significant effect was observed in the first, second and third hour of the experiment, inhibiting the development of edema by 51.2%, 43.8% and 48.1%, respectively. Compound **22** inhibited the formation of edema by 37.1%, 18.7% and 30.8% in three consecutive hours of the experiment, respectively, but the effect was not statistically significant. Pretreatment with compound **19** reduced paw edema by 28.2%, 35.7% and 17.3%, in the first, second and third hour of the experiment, respectively, compared to the control group, but the effect was not statistically significant. 

#### 2.6.2. Anti-Nociceptive Activity in the Formalin Test

In the formalin test, which is a model of chronic pain induced by the administration of a 5% formalin solution, the evaluated compounds showed antinociceptive activity. Injection of formalin into the dorsal surface of the hind paw of a mouse produces a biphasic nocifensive behavioral response; i.e., licking, biting, flinching or lifting of the injected paw. The acute (neurogenic) nociceptive phase lasts for the first 5 min and is followed by a period of little activity for the next 10 min. The first phase of the test is directly associated with nociceptor stimulation and the development of neurogenic inflammation. The second (late) phase occurs between 15 and 30 min after formalin injection. The second phase depends on peripheral inflammation and the central sensitization of pain. Since this phase reflects the activation of inflammatory processes, the compounds active in this phase of the experiment also have an anti-inflammatory effect. The tested compounds **19**, **22** and **24**, were investigated in three doses: 10, 20 and 40 mg/kg bw. **ASA** (acetylsalicylic acid) was used as a reference compound and administered in doses of 50, 100 and 200 mg/kg. All compounds only reduced the licking/biting time of the right hind paw of mice in the II phase of the test (15–30 min) (Table 6) and showed antinociceptive activity. This effect was observed for each of these compounds, only at a dose of 40 mg/kg. The strongest effect in this assay was produced by **24**. The calculated ED_50_ value for this compound is 9.1 mg/kg. In addition, this anti-nociceptive and anti-inflammatory effect was approximately 13.8 times greater than that observed for the reference compound *(***ASA**). Compounds **19** and **22** significantly reduced the duration of the licking response in the late (inflammatory) phase by 47.2% (*p* < 0.05) and 48.0% (*p* < 0.05), respectively. **ASA** also attenuated statistically significant pain responses in the late phases of the formalin test. The calculated value of ED_50_ is 126.3 mg/kg. 

## 3. Conclusions

Twenty-five novel xanthine derivatives with varied substituents in the *N1-*, *N3-* and C8-positions were designed and synthesized. Compounds were divided into two series (I and II). The division depended on the position of the introduced substituents: series I—*N1* and C8; series II—*N1*, *N3* and C8. In vitro binding studies at human adenosine receptors showed that these compounds had:

✓ interaction with A_3_R at submicromolar concentrations,
✓ no interaction with A_2B_R at submicromolar concentrations (except for compounds **5** and **14**),
✓ affinities for A_1_R in the nanomolar range in the series IC and II (except **20** and **26**), and
✓ variable affinities for A_2A_R (most compounds had K_i_ values in the range from 62 nM to 773 nM)

**Figure 9:**
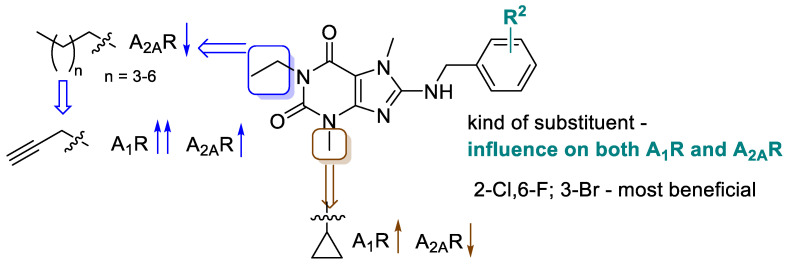
SAR analysis of synthesized xanthine derivatives **5**–**29**. (Arrow up: increase of affinity; arrow down: decrease of affinity).

Analysis of SARs indicated chemical features in the presented scaffold that are important for the enhancement of affinity and selectivity for the subtypes of adenosine receptors. A summary is shown in Figure 9.

The propargyl substituent in the *N1*-position led to affinities for A_1_R in the nanomolar range. Additionally, the exchange of the methyl for a cyclopropyl in the *N3*-position increased affinities for A_1_R, but slightly decreased those for the A_2A_R. The most beneficial substituents in the benzylamine group were 2-chloro-6-fluro and 3-bromo. 

Among all synthesized compounds, only two compounds, **22** and **24** (IC series), had affinities for A_2A_R comparable to that of the lead structure **2** (**22**: K_i_ = 62 nM; **24**: K_i_ = 77 nM vs. **2**: K_i_ = 71 nM). Compound **24** (the 3-bromobenzylamine derivative) also showed a simultaneously strong affinity for the A_1_R and is, therefore, a dual ligand with balanced affinities for both receptors (A_1_R *K*_i_ = 72 nM; A_2A_R *K*_i_ = 77 nM). In series II, compound **29** was obtained, which had a two-fold higher affinity for the A_1_R than for the A_2A_R (A_1_R *K*_i_ = 78 nM; A_2A_R *K*_i_ = 204 nM). Three compounds (**19, 22** and **24**), being the most promising dual ligands, were selected for further studies. 

Docking to the A_1_R and A_2A_R showed differences in positioning and interactions with amino acid residues in the binding pocket, suggesting the reasons for differences in affinities of the compounds for these receptors. Toxicity studies confirmed low hepato- and neurotoxicity up to a concentration of 12.5 µM. Higher concentrations caused compound precipitation in the culture medium (PBS). The compounds displayed high metabolic stability. The main metabolite formed for all of them was a product with a molecular weight of 249, which suggests that this product is a result of fragmentation (after dissociation of the benzyl substituent from the amino group at the C8 position of the xanthine moiety) with simultaneous hydroxylation in the propargyl chain. 

Anti-inflammatory studies in the Griess assay showed that compounds **19** and **22** had a moderate positive effect on nitric oxide production in BV2 cells, but only in the lowest tested concentrations (100 and/or 200 nM). Therefore, the most promising compound **22** was chosen for further in vitro evaluation and its influence on phagocytosis was tested in BV-2 cells using the IncuCyte imaging platform. Results showed that compound **22** (tested at a concentration of 1 µM) protected from phagocytosis.

Furthermore, in vivo studies showed that the most promising of the three tested compounds (**19**, **22** and **24**) was compound **24**, which, at the tested doses (20 mg/kg bw in the carrageenan test and 40 mg/kg in the formalin test), displayed anti-inflammatory activity.

Undoubtedly, our work has certain limitations, which may result from the conditions and procedure of the conducted studies, and may have influenced the results obtained. First of all, we can see that the problem is the limited solubility of our compounds in various solvents, especially in higher concentrations, which are indicated for the determination of a special parameter; e.g., *P_e_* in the PAMPA assay. In this assay, it is necessary to use high concentrations (from 100 to 200 µM [28]) in order to increase the sensitivity of the assay and mimic the composition of specific target barriers [29]. We did not accomplish better solubility of our compounds by increasing the concentrations of DMSO up to 5%, which is the highest concentration compatible with pre-coated filter plate. Moreover, xanthine derivatives are molecules, which are difficult to convert into salts because they do not have a sufficiently basic center (it is known that the formation of salts usually increases the solubility of compounds [30]). Furthermore, this work is our initial work that evaluates the anti-inflammatory activity of compounds from this group in the Griess assay and using the IncuCyte Live-Cell Analysis System. It has led to interesting results that encourage further studies to verify the anti-inflammatory activity of dual A_2A_/A_1_ receptor ligands. 

Thus, dual ligands with promising anti-inflammatory activity, found in the group of novel tri-substituted xanthines, could be useful tools for the future development of adenosine receptor antagonists as drugs for neuroinflammatory diseases.

## 4. Material and Methods

### 4.1. Chemistry

#### 4.1.1. General Information

All commercially available reagents and solvents were purchased and used without further purification. Melting points (mp.) were determined on a MEL-TEMP II (LD Inc., Long Beach, CA, USA) melting point apparatus and were uncorrected. ^1^H-NMR spectra were recorded on a Varian Mercury 300 MHz or JEOL FT-NMR 500 MHz apparatus in CDCl_3_ or in DMSO-d6 using tetramethylsilane as an internal standard. ^13^C NMR data were recorded on 75 MHz on a Varian-Mercury-VX 300 MHz PFG or JEOL FT-NMR 500 MHz spectrometer. The *J* values are reported in Hertz (Hz), and the splitting patterns are designated as follows: br s (broad singlet), d (doublet), dd (doublet of doublets), dt (doublet of triplets), m (multiplet), s (singlet), t (triplet), tt (triplet of triplets) and quin (quintet). Data are reported in order: Chemical shift, multiplicity, coupling constant, number of protons and proton’s position (cyclopropyl, phe-phenyl, xan-xanthine). The purity of the tested compounds was determined (%) on a Waters TQD mass spectrometer coupled with a Waters ACQUITY UPLC system. Retention times (*t*_R_) are given in minutes. The reactions were monitored by thin layer chromatography (TLC) using aluminum sheets coated with silica gel 60F254 (Merck) using as a developing system dichloromethane/metanol 9:1. Spots were detected under UV light.

#### 4.1.2. Synthesis of Compounds **5**–**26**

*Synthesis of 8-bromotheobromine*

To theobromine (40 mmol, 7.21 g) in 28 mL of glacial acetic acid, 48% HBr was added and the mixture was heated in a water bath (bath temperature 80 °C) for 30 min. Then, a solution of sodium chlorate (V) in water was dropped over a period of 1 h. Next, the temperature of the bath was increased to boiling and heating was continued with stirring for 1.5–2 h. After that time, the reaction mixture was left at room temperature (for several hours). The precipitate was filtered off and washed several times with cold water. The reaction was repeated several times. Yield: 40–50%. 

*General procedure of synthesis of 1-substituted 8-bromotheobromines* (**3A**–**3H**)

A mixture of 8-bromotheobromine (20 mmol, 5.18 g), halide (22 mmol) and K_2_CO_3_ (60 mmol, 8.30 g) in 40 mL of DMF was heated at 110 °C from 4 h to 18 h. Next, water was added and the resulting precipitate was filtered and washed with water and/or 1% NaOH. The product was directly used for the next step of the synthesis or purified by boiling in ethanol.

*1-Propyl-8-bromotheobromine* (**3A**)

Synthesis from propyl iodide. Yield: 74%, C_10_H_13_BrN_4_O_2_ (MW 301.14). 

*1-Butyl-8-bromotheobromine* (**3B**)

Synthesis from n-butyl iodide. Yield: 15%, C_11_H_15_ClN_4_O_2_ (MW 315.17). Synthesis of this compound from n-butyl bromide was described by Patrushev et al. [31] (yield: 81%; mp: 133–136 °C)

*1-Pentyl-8-bromotheobromine* (**3C**)

Synthesis from pentyl bromide. Yield: 73%, C_12_H_17_BrN_4_O_2_ (MW 329.20). 

*1-Heksyl-8-bromotheobromine* (**3D**)

Synthesis from hexyl bromide. Yield: 27%, C_13_H_19_BrN_4_O_2_ (MW 343.23). 

*1-Benzyl-8-bromotheobromine* (**3E**)

Synthesis from benzyl chloride. Yield: 42%, C_14_H_13_BrN_4_O_2_ (MW 349.19).

*1-(4-Chlorobenzyl-8-bromotheobromine* (**3F**)

Synthesis from 4-chlorobenzyl chloride. Yield: 67%, C_14_H_12_BrClN_4_O_2_ (MW 383.63).

*1-(3,4-Dichlorobenzyl-8-bromotheobromine* (**3G**)

Synthesis from 3,4-dichlorobenzyl chloride. Yield: 41%, C_14_H_11_BrCl_2_N_4_O_2_ (MW 418.07).

*1-(Prop-2-yn-1-yl)-8-bromotheobromine* (**3H**)

Synthesis from 3-bromoprop-1-yne. Yield: 41%, C_10_H_9_BrN_4_O_2_ (MW 297.11)

General procedure for the synthesis of 8-substitued benzylaminoxanthines: **5, 12**–**26**

A mixture of 0.55 mmoles of 8-bromo-1-propyl-3,7-dimethyl-3,7-dihydro-1H-purine-2,6-dione (**3A**), 1.1 mmoles of appropriate benzylamine, 1.6 mmoles of TEA and 1.00 mL of propanol was heated in closed vessels in a microwave oven (CEM Discover SC, 300 Watt, Power Max Off, 150 °C, 10 bar) for 1 h. The solvent was removed and the residue was treated with DCM. The products were purified by crystallization from ethanol or flash column chromatography over silica gel with CH_2_Cl_2_:MeOH (100:0 to 80:20).

*8-((2-Chlorobenzyl)amino)-3,7-dimethyl-1-propyl-3,7-dihydro-1H-purine-2,6-dione* (**5**)

Synthesis from **3A** (0.55 mmol; 0.157 g) and 2-chlorobenzylamine (1.1 mmol, 0.156 g). Yield: 48% (96 mg), mp: 190–191 °C, C_17_H_20_ClN_5_O_2_ (MW 361.83). ^1^H NMR (300 MHz, DMSO-*d*_6_) δ: 0.78–0.86 (m, 3H, *N1*CH_2_CH_2_CH_3_) 1.42–1.57 (m, 2H, *N1*CH_2_CH_2_) 3.26 (s, 3H, *N3*CH_3_) 3.62 (s, 3H, *N7*CH_3_) 3.70–3.79 (m, 2H, *N1*CH_2_) 4.60 (d, *J* = 5.86 Hz, 2H, NHCH_2_) 7.24–7.34 (m, 2H, phe-4,5-H) 7.40–7.47 (m, 2H, phe-3,6-H) 7.60 (t, *J* = 5.86 Hz, 1H, NHCH_2_). ^13^C NMR (DMSO-*d*_6_) δ: 11.7 (*N1*CH_2_CH_2_CH_3_), 21.4 (*N1*CH_2_CH_2_), 29.7 (*N7*CH_3_), 30.3 (*N3*CH_3_), 42.0 (*N1*CH_3_), 43.9 (NHCH_2_), 102.6 (Xan-C5), 127.6 (phe-C5), 129.1 (phe-C4), 129.4 (phe-C6), 129.6 (phe-C3), 132.5 (phe-C2), 136.9 (phe-C1), 148.6 (xan-C4), 151.1 (xan-C2), 153.3 (xan-C6), 154.2 (xan-C8). UPLC/MS purity > 99%; *t*_R_ = 6.42, (ESI) *m*/*z* [M]^+^ 362.2.

*8-((2-Bromobenzyl)amino)-3,7-dimethyl-1-propyl-3,7-dihydro-1H-purine-2,6-dione* (**12**)

Synthesis from **3A** (0.55 mmol; 0.157 g) and 2-bromobenzylamine (1.1 mmol, 0.204 g). Yield: 98 mg (44%), mp: 206–207 °C, C_17_H_20_BrN_5_O_2_ (MW 406.28). ^1^H NMR (300 MHz, DMSO-*d*_6_) δ: 0.78–0.85 (m, 3H, *N1*CH_2_CH_2_CH_3_) 1.43–1.56 (m, 2H, *N1*CH_2_CH_2_) 3.26 (s, 3H, *N3*CH_3_) 3.63 (s, 3H, *N7*CH_3_) 3.71–3.78 (m, 2H, *N1*CH_2_) 4.56 (d, *J* = 5.86 Hz, 2H, NHCH_2_) 7.16–7.24 (m, 1H, phe-4-H) 7.31–7.45 (m, 2H, phe-5,6-H) 7.57–7.66 (m, 2H, phe-3-H + NHCH_2_). ^13^C NMR (DMSO-*d*_6_) δ: 11.7 (*N1*CH_2_CH_2_CH_3_), 21.5 (*N1*CH_2_CH_2_), 29.8 (*N7*CH_3_), 30.4 (*N3*CH_3_), 42.0 (*N1*CH_3_), 46.5 (NHCH_2_), 102.7 (xan-C5), 122.9 (phe-C2), 128.3 (phe-C5), 129.4 (phe-C4), 129.5 (phe-C6), 132.9 (phe-C3), 138.5 (phe-C1), 148.7 (xan-C4), 151.2 (xan-C2), 153.4 (xan-C6), 154.3 (xan-C8). UPLC-MS purity 97.5%, *t*_R_ = 6.57, (ESI) *m*/*z* [M]^+^ 406.13. 

*8-((2-Fluorobenzyl)amino)-3,7-dimethyl-1-propyl-3,7-dihydro-1H-purine-2,6-dione* (**13**)

Synthesis from **3A** (0.55 mmol; 0.157 g) and 2-flurobenzylamine (1.1 mmol, 0.138 g). Yield: 74 mg (39%), mp: 224–226 °C, C_17_H_20_FN_5_O_2_ (MW 345.38). ^1^H NMR (300 MHz, DMSO-*d*_6_) δ: 0.78–0.85 (m, 3H, *N1*CH_2_CH_2_CH_3_), 1.42–1.56 (m, 2H, *N1*CH_2_CH_2_), 3.28 (s, 3H, *N3*CH_3_), 3.58 (s, 3H, *N7*CH_3_), 3.70–3.77 (m, 2H, *N1*CH_2_), 4.56 (d, *J* = 5.86 Hz, 2H, NHCH_2_), 7.11–7.20 (m, 2H, phe-5,6-H), 7.24–7.34 (m, 1H, phe-3-H), 7.44 (td, *J* = 7.77, 1.47 Hz, 1H, phe-4-H), 7.57 (t, *J* = 5.86 Hz, 1H, NHCH_2_). ^13^C NMR (DMSO-*d*_6_) δ: 11.6 (*N1*CH_2_CH_2_CH_3_), 21.4 (*N1*CH_2_CH_2_), 29.6 (*N7*CH_3_), 30.3 (*N3*CH_3_), 41.9 (*N1*CH_2_), 102.6 (xan-C5), 115.5 (d, ^2^*J*_C,F_ = 21.8 Hz, phe-C3), 124.7 (d, ^4^*J*_C,F_ = 3.5 Hz, phe-C5), 126.6 (d, ^2^*J*_C,F_ = 15.0 Hz, phe-C4), 129.4 (d, ^3^*J*_C,F_ = 8.1 Hz, phe-C6), 130.1 (d, ^3^*J*_C,F_ = 3.4 Hz, phe-C1), 148.6 (xan-C4), 151.1 (xan-C2), 153.3 (xan-C6), 154.2 (xan-C8), 160.6 (d, ^1^*J*_C,F_ = 244.2 Hz, phe-C2). UPLC/MS purity > 99%; *t*_R_ = 5.98, (ESI) *m/z* [M]^+^ 346.18.

*8-((2-Chloro-6-fluorobenzyl)amino)-3,7-dimethyl-1-propyl-3,7-dihydro-1H-purine-2,6-dione* (**14**)

Synthesis from **3A** (0.55 mmol; 0.157 g) and 2-chloro-6-flurobenzylamine (1.1 mmol, 0.176 g). Yield: 110 mg (53%), mp: 241–243 °C, C_17_H_19_ClFN_5_O_2_ (MW 379.82). ^1^H NMR (300 MHz, CDCl_3_-*d*) δ: 0.92 (t, *J* = 7.33 Hz, 3H, *N1*CH_2_CH_2_CH_3_), 1.56–1.72 (m, 2H, *N1*CH_2_CH_2_), 3.52 (s, 3H, *N3*CH_3_), 3.65 (s, 3H, *N7*CH_3_), 3.82–3.97 (m, 2H, *N1*CH_2_), 4.83 (s, 3H, NHCH_2_ + NHCH_2_) 6.94–7.06 (m, 1H, phe-4-H) 7.15–7.24 (m, 2 H, phe-3,5-H); ^13^C NMR (CDCl_3_-*d*) δ: 11,4 (*N1*CH_2_CH_2_CH_3_), 21.4 (*N1*CH_2_CH_2_), 29.7 (*N7*CH_3_/*N3*CH_3_), 38.8 (d, ^3^*J*_C,F_ = 4.6 Hz, NHCH_2_), 42.5 (*N1*CH_3_), 103.4 (xan-C5), 114.4 (d, ^2^*J*_C,F_ = 24.0 Hz, phe-C5), 123.7 (d, ^2^*J*_C,F_ = 17.3 Hz, phe-C1), 125.3 (d, ^4^*J*_C,F_ = 4.2 Hz, phe-C3), 130.0 (d, ^3^*J*_C,F_ = 10.3 Hz, phe-C4), 135.5 (phe-C2), 148.1 (xan-C4), 151.5 (xan-C2), 152.5 (xan-C6), 154.2 (xan-C8), 161.7 (d, ^1^*J*_C,F_ = 251.0 Hz, phe-C6). UPLC-MS purity > 99%; *t*_R_ = 6.39; (ESI) *m/z* [M]^+^ 380.21.

*8-((3-Chlorobenzyl)amino)-3,7-dimethyl-1-propyl-3,7-dihydro-1H-purine-2,6-dione* (**15**)

Synthesis from **3A** (0.55 mmol; 0.157 g) and 3-chlorobenzylamine (1.1 mmol, 0.156 g).

Yield: 111 (56%), mp: 185–187 °C, C_17_H_20_ClN_5_O_2_ (MW 361.83). ^1^H NMR (500 MHz, DMSO-*d*_6_) δ: 0.79 (t, *J* = 7.45 Hz, 3H, *N1*CH_2_CH_2_CH_3_), 1.47 (dq, *J* = 14.61, 7.35 Hz, 2H, *N1*CH_2_CH_2_), 3.26 (s, 3H, *N3*CH_3_), 3.56 (s, 3H, *N7*CH_3_), 3.72 (t, *J* = 7.45 Hz, 2H, *N1*CH_2_), 4.50 (d, *J* = 5.73 Hz, 2H, NHCH_2_), 7.23–7.34 (m, 3H, phe-2,5,6-H), 7.38–7.43 (m, 1H, phe-4-H), 7.54–7.63 (m, 1H, NHCH_2_); ^13^C NMR (DMSO-*d*_6_) δ: 11.7 (*N1*CH_2_CH_2_CH_3_), 21.5 (*N1*CH_2_CH_2_), 29.7 (*N7*CH_3_), 30.4 (*N3*CH_3_), 42.0 (*N1*CH_2_), 45.7 (NHCH_2_), 102.7 (xan-C5), 126.6 (phe-C4), 127.4 (phe-C2), 127.7 (phe-C6), 130.7 (phe-C5), 133.5 (phe-C1), 142.8 (phe-C3), 148.7 (xan-C4), 151.2 (xan-C2), 153.4 (xan-C6), 154.4 (xan-C8). UPLC-MS purity 96.2%; *t*_R_ = 6.48; (ESI) *m*/*z* [M]^+^ 362.2.

*8-((3-Bromobenzyl)amino)-3,7-dimethyl-1-propyl-3,7-dihydro-1H-purine-2,6-dione* (**16**)

Synthesis from **3A** (0.55 mmol; 0.157 g) and 3-bromobenzylamine (1.1 mmol, 0.204 g). Yield: 92 mg (41%), mp: 220–222 °C, C_17_H_20_BrN_5_O_2_ (MW 406.28). ^1^H NMR (300 MHz, DMSO-*d*_6_) δ: 0.81 (t, *J* = 7.33 Hz, 3H, *N1*CH_2_CH_2_CH_3_), 1.43–1.57 (m, 2H, *N1*CH_2_CH_2_), 3.29 (s, 3H, *N3*CH_3_), 3.58 (s, 3H, *N7*CH_3_), 3.70–3.78 (m, 2H, *N1*CH_2_), 4.51 (d, *J* = 5.86 Hz, 2H, NHCH_2_), 7.23–7.46 (m, 3H, phe-2,5,6-H), 7.56 (s, 1H, phe-4-H), 7.62 (t, *J* = 6.15 Hz, 1H, NHCH_2_); ^13^C NMR (DMSO-*d*_6_) δ: 11.7 (*N1*CH_2_CH_2_CH_3_), 21.4 (*N1*CH_2_CH_2_), 29.7 (*N7*CH_3_), 30.3 (*N3*CH_3_), 41.9 (NHCH_2_), 45.6 (*N1*CH_2_), 102.6 (xan-C5), 122.0 (phe-C3), 126.9 (phe-C6), 130.2 (phe-C5), 130.6 (phe-C4), 130.9 (phe-C2), 142.9 (phe-C1), 148.6 (xan-C4), 151.1 (xan-C2), 153.3 (xan-C6), 154.2 (xan-C8). UPLC-MS purity 96.8%; *t*_R_ = 6.62; (ESI) *m/z* [M + 2]^+^ 408.12.

*8-((3-methoxybenzyl)amino)-3,7-dimethyl-1-propyl-3,7-dihydro-1H-purine-2,6-dione* (**17**) 

Synthesis from **3A** (0.55 mmol; 0.157 g) and 3-methoxybenzylamine (1.1 mmol, 0.151 g). Yield: 116 mg (59%), mp: 173–175 °C, C_18_H_23_N_5_O_3_ (MW 357.41). ^1^H NMR (500 MHz, DMSO-*d*_6_) δ: 0.80 (t, *J* = 7.16 Hz, 3H, *N1*CH_2_CH_2_CH_3_), 1.44–1.52 (m, 2H, *N1*CH_2_CH_2_), 3.28 (s, 3H, *N3*CH_3_), 3.56 (s, 3H, *N7*CH_3_), 3.68–3.74 (m, 5H, *N1*CH_2_ + OCH_3_), 4.47 (d, *J* = 5.16 Hz, 2H, NHCH_2_), 6.77 (d, *J* = 6.30 Hz, 1H, phe-4-H), 6.88–6.93 (m, 2H, phe-2,6-H), 7.20 (t, *J* = 7.73 Hz, 1H, phe-5-H), 7.53 (br. s., 1H, NHCH_2_); ^13^C NMR (DMSO-*d*_6_) δ: 11.7 (*N1*CH_2_CH_2_CH_3_), 21.5 (*N1*CH_2_CH_2_), 29.7 (*N7*CH_3_), 30.3 (*N3*CH_3_), 42.0 (*N1* CH_2_CH_2_CH_3_), 46.2 (NHCH_2_), 55.5 (OCH_3_), 102.6 (xan-C5), 112.8 (phe-C2), 113.6 (phe-C4), 120.1 (phe-C6), 129.9 (phe-C5), 141.8 (phe-C1), 148.8 (xan-C4), 151.3 (xan-C2), 153.3 (xan-C6), 154.6 (xan-C8), 159.8 (phe-C3). UPLC-MS purity 98.5%; *t*_R_ = 5.79; (ESI) *m*/*z* [M + H]^+^ 358.27.

*8-((3,4-dimethoxybenzyl)amino)-3,7-dimethyl-1-propyl-3,7-dihydro-1H-purine-2,6-dione* (**18**)

Synthesis from **3A** (0.55 mmol; 0.157 g) and 3,4-dimethoxybenzylamine (1.1 mmol, 0.184 g). Yield: 140 mg (66%), mp: 200–201 °C, C_19_H_25_N_5_O_4_ (MW 387.44). ^1^H NMR (300 MHz, DMSO-*d*_6_) δ: 0.81 (t, *J* = 7.33 Hz, 3H, *N1*CH_2_CH_2_CH_3_), 1.43–1.55 (m, 2H, *N1*CH_2_CH_2_), 3.32 (s, 3H, *N3*CH_3_), 3.57 (s, 3H, *N7*CH_3_), 3.68–3.75 (m, 8H, *N1*CH_2_ +2 x OCH_3_), 4.43 (d, *J* = 5.86 Hz, 2H, NHCH_2_), 6.87 (s, 2H, phe-5,6-H), 7.01 (s, 1H, phe-1-H), 7.49 (t, *J* = 5.86 Hz, 1H, NHCH_2_); ^13^C NMR (DMSO-*d*_6_) δ: 11.7 (*N1*CH_2_CH_2_CH_3_), 21.4 (*N1*CH_2_CH_2_), 29.6 (*N7*CH_3_), 30.2 (*N3*CH_3_), 41.9 (*N1*CH_3_), 46.0 (NHCH_2_), 55.8 (OCH_3_), 56.0 (OCH_3_), 102.4 (xan-C5), 112.0 (phe-C2), 112.1 (phe-C5), 120.1 (phe-C6), 132.4 (phe-C1), 148.3 (phe-C4), 148.7 (xan-C4), 149.0 (phe-C3), 151.2 (xan-C2), 153.2 (xan-C6), 154.5 (xan-C8). UPLC-MS purity 98.7%; *t*_R_ = 5.23; (ESI) *m*/*z* [M + H]^+^ 388.25.

*8-((2-Chlorobenzyl)amino)-3,7-dimethyl-1-(prop-2-yn-1-yl)-3,7-dihydro-1H-purine-2,6-dione* (**19**)

Synthesis from **3H** (0.55 mmol; 0.065 g) and 2-chlorobenzylamine (1.1 mmol, 0.156 g). Yield: 110 mg (56%), mp: 223–225 °C, C_17_H_16_ClN_5_O_2_ (MW 357.80). ^1^H NMR (300 MHz, DMSO-*d*_6_) δ: 3.02 (t, *J* = 2.34 Hz, 1H, C≡CH), 3.28 (s, 3H, *N3*CH_3_), 3.62 (s, 3H, *N7*CH_3_), 4.52 (d, *J* = 2.34 Hz, 2H, *N1*CH_2_), 4.61 (d, *J* = 5.27 Hz, 2H, NHCH_2_), 7.24–7.34 (m, 2H, phe-5,6-H), 7.41–7.46 (m, 2H, phe-3,4-H), 7.68 (t, *J* = 5.86 Hz, 1H, NHCH_2_); ^13^C NMR (DMSO-*d*_6_) δ: 29.8 (*N7*CH_3_), 29.9 (*N3*CH_3_), 30.4 (*N1*CH_2_), 43.9, (NHCH_2_), 72.9 (C≡CH), 80.5 (C≡CH), 102.5 (xan-C5), 127.6 (phe-C5), 129.2 (phe-C4), 129.4 (phe-C6), 129.6 (phe-C3), 132.5 (phe-C2), 136.8 (phe-C1), 149.0 (xan-C4), 150.6 (xan-C2), 152.2 (xan-C6), 154.5 (xan-C8). UPLC-MS purity 96.7%; *t*_R_ = 5.97; (ESI) *m*/*z* [M]^+^ 358.21.

*8-((2-Bromobenzyl)amino)-3,7-dimethyl-1-(prop-2-yn-1-yl)-3,7-dihydro-1H-purine-2,6-dione* (**20**)

Synthesis from **3H** (0.55 mmol; 0.065 g) and 2-bromobenzylamine (1.1 mmol, 0.204 g). Yield: 137 mg (62%), mp: 210–212 °C, C_17_H_16_BrN_5_O_2_ (MW 402.25). ^1^H NMR (300 MHz, DMSO-*d*_6_) δ: 3.02 (t, *J* = 2.34 Hz, 1H, C≡CH), 3.29 (s, 3H, *N3*CH_3_), 3.63 (s, 3H, *N7*CH_3_), 4.52 (d, *J* = 2.34 Hz, 2H, *N1*CH_2_), 4.57 (d, *J* = 5.86 Hz, 2H, NHCH_2_), 7.17–7.24 (m, 1H, phe-4-H), 7.31–7.45 (m, 2H, phe-5,6-H), 7.60 (dd, *J* = 7.62, 1.17 Hz, 1H, phe-3-H), 7.70 (t, *J* = 5.86 Hz, 1H, NHCH_2_); ^13^C NMR (DMSO-*d*_6_) δ: 29.8 (*N7*CH_3_), 29.9 (*N3*CH_3_), 30.4 (*N1*CH_2_), 46.4 (NHCH_2_), 72.9 (C≡CH), 80.5 (C≡CH), 102.5 (xan-C5), 122.8 (phe-C2), 128.2 (phe-C5), 129.4 (phe-C4), 129.5 (phe-C6), 132.8 (phe-C3), 138.3 (phe-C1), 149.0 (xan-C4), 150.1 (xan-C2), 152.2 (xan-C6), 154.4 (xan-C8). UPLC-MS purity 95.1%; *t*_R_ = 6.10; (ESI) *m*/*z* [M]^+^ 402.14.

*8-((2-Fluorobenzyl)amino)-3,7-dimethyl-1-(prop-2-yn-1-yl)-3,7-dihydro-1H-purine-2,6-dione* (**21**)

Synthesis from **3H** (0.55 mmol; 0.065 g) and 2-flurobenzylamine (1.1 mmol, 0.138 g). Yield: 90 mg (48%), mp: 224–226 °C, C_17_H_16_FN_5_O_2_ (MW 341.35). ^1^H NMR (300 MHz, DMSO-*d*_6_) δ: 3.02 (t, *J* = 2.34 Hz, 1H, C≡CH), 3.31 (s, 3H, *N3*CH_3_), 3.59 (s, 3H, *N7*CH_3_), 4.52 (d, *J* = 2.34 Hz, 2H, *N1*CH_2_), 4.57 (d, *J* = 5.86 Hz, 2H, NHCH_2_), 7.12–7.21 (m, 2H, phe-5,6-H), 7.26–7.35 (m, 1H, phe-3-H), 7.44 (td, *J* = 7.77, 1.47 Hz, 1H, phe-4-H), 7.65 (t, *J* = 5.57 Hz, 1H, NHCH_2_); ^13^C NMR (DMSO-*d*_6_) δ: 29.8 (*N7*CH_3_), 29.9 (*N3*CH_3_), 30.4 (*N1*CH_2_), 72.9 (C≡CH), 80.5 (C≡CH), 102.4 (xan-C5), 115.9 (d, ^2^*J*_C,F_ = 20.7 Hz, phe-C3), 124.8 (d, ^4^*J*_C,F_ = 3.5 Hz, phe-C5), 126.5 (d, ^2^*J*_C,F_ = 15.0 Hz, phe-C4), 129.5 (d, ^3^*J*_C,F_ = 8.0 Hz, phe-C6), 130.2 (d, ^3^*J*_C,F_ = 3.5 Hz, phe-C1), 149.0 (xan-C4), 150.6 (xan-C2), 152.2 (xan-C6), 154.5 (xan-C8), 160.6 (d, ^1^*J*_C,F_ = 244.2 Hz, phe-C2). UPLC-MS purity 98.7%; *t*_R_ = 5.53; (ESI) *m*/*z* [M + H]^+^ 342.19. 

*8-((2-Chloro-6-fluorobenzyl)amino)-3,7-dimethyl-1-(prop-2-yn-1-yl)-3,7-dihydro-1H-purine-2,6-dione* (**22**)

Synthesis from **3H** (0.55 mmol; 0.157 g) and 2-chloro-6-flurobenzylamine (1.1 mmol, 0.176 g). Yield: 114 mg (55%), mp: 276–278 °C, C_17_H_15_ClFN_5_O_2_ (MW 375.79). ^1^H NMR (300 MHz, DMSO-*d*_6_) δ: 2.99–3.02 (m, 1H, C≡CH), 3.35 (s, 3H, *N3*CH_3_), 3.53 (s, 3H, *N7*CH_3_), 4.51 (d, *J* = 2.34 Hz, 2H, *N1*CH_2_), 4.62 (dd, *J* = 5.27, 1.17 Hz, 2H, NHCH_2_), 7.18–7.26 (m, 1H, phe-4-H), 7.30–7.41 (m, 2H, phe-3,5-H), 7.41–7.46 (m, 1H, NHCH_2_); ^13^C NMR (DMSO-*d*_6_) δ: 29.7 (*N7*CH_3_) 29.9 (*N3*CH_3_), 30.5 (*N1*CH_2_), 38.5 (d, ^3^*J*_C,F_ = 4.6 Hz, NHCH_2_), 72.8 (C≡CH), 80.6 (C≡CH), 102.4 (xan-C5), 114.9 (d, ^2^*J*_C,F_ = 23.0 Hz, phe-C5), 124.1 (d, ^2^*J*_C,F_ = 17.3 Hz, phe-C1), 125.9 (d, ^4^*J*_C,F_ = 4.6 Hz, phe-C3), 130.8 (d, ^3^*J*_C,F_ = 9.3 Hz, phe-C4), 135.0 (d, ^3^*J*_C,F_ = 5.8 Hz, phe-C2), 149.0 (xan-C4), 150.6 (xan-C2), 152.2 (xan-C6), 154.1 (xan-C8), 162.0 (d, ^1^*J*_C,F_ = 248.8 Hz, phe-C6). UPLC-MS purity 96.0%; *t*_R_ = 5.92; (ESI) *m*/*z* [M]^+^ 376.15.

*8-((3-Chlorobenzyl)amino)-3,7-dimethyl-1-(prop-2-yn-1-yl)-3,7-dihydro-1H-purine-2,6-dione* (**23**)

Synthesis from **3H** (0.55 mmol; 0.065 g) and 3-chlorobenzylamine (1.1 mmol, 0.156 g). Yield: 116 mg (59%), mp: 234–236 °C, C_17_H_16_ClN_5_O_2_ (MW 357.80). ^1^H NMR (300 MHz, DMSO-*d*_6_) δ: 3.01 (t, *J* = 2.34 Hz, 1H, C≡CH), 3.31 (s, 3H, *N3*CH_3_), 3.59 (s, 3H, *N7*CH_3_), 4.51–4.55 (m, 4H, *N1*CH_2_ + NHCH_2_), 7.27–7.36 (m, 3H, phe-4,5,6-H), 7.42 (s, 1H, phe-2-H), 7.68 (t, *J* = 6.15 Hz, 1H, NHCH_2_); ^13^C NMR (DMSO-*d*_6_) δ: 29.8 (*N7*CH_3_), 29.9 (*N3*CH_3_), 30.3 (*N1*CH_2_), 45.6 (NHCH_2_), 72.9 (C≡CH), 80.5 (C≡CH), 102.4 (xan-C5), 126.5 (phe-C6), 127.4 (phe-C5), 127.7 (phe-C4), 130.6 (phe-C2), 133.4 (phe-C1), 142.6 (phe-C3), 149.0 (xan-C4), 150.6 (xan-C2), 152.2 (Cxan-6), 154.5 (xan-C8). UPLC-MS purity 97.2%; *t*_R_ = 6.03; (ESI) *m*/*z* [M]^+^ 358.21.

*8-((3-Bromobenzyl)amino)-3,7-dimethyl-1-(prop-2-yn-1-yl)-3,7-dihydro-1H-purine-2,6-dione* (**24**)

Synthesis from **3H** (0.55 mmol; 0.065 g) and 3-bromobenzylamine (1.1 mmol, 0.204 g). Yield: 91 mg (41%), mp: 248–250 °C, C_17_H_16_BrN_5_O_2_ (MW 402.25). ^1^H NMR (300 MHz, DMSO-*d*_6_) δ: 3.01–3.04 (m, 1H, C≡CH), 3.31 (s, 3H, *N3*CH_3_), 3.59 (s, 3H, *N7*CH_3_), 4.50–4.54 (m, 4H, *N1*CH_2_ +NHCH_2_), 7.24–7.46 (m, 3H, phe-2,5,6-H), 7.56 (d, *J* = 1.76 Hz, 1H, phe-4-H), 7.69 (t, *J* = 6.15 Hz, 1H, NHCH_2_); ^13^C NMR (DMSO-*d*_6_) δ: 29.8 (*N7*CH_3_), 29.9 (*N3*CH_3_), 30.4 (*N1*CH_2_), 45.6 (NHCH_2_), 72.9 (C≡CH), 80.5 (C≡CH), 110.0 (xan-C5), 122.0 (phe-C3), 126.9 (phe-C6), 130.3 (phe-C5), 130.6 (phe-C4), 131.0 (phe-C2), 142.8 (phe-C1), 149.0 (xan-C4), 150.6 (xan-C2), 152.2 (xan-C6), 154.5 (xan-C8). UPLC-MS purity 97.8%; *t*_R_ = 6.16; (ESI) *m*/*z* [M]^+^ 402.07.

*8-((3-Methoxybenzyl)amino)-3,7-dimethyl-1-(prop-2-yn-1-yl)-3,7-dihydro-1H-purine-2,6-dione* (**25**)

Synthesis from **3H** (0.55 mmol; 0.065 g) and 3-methoxybenzylamine (1.1 mmol, 0.151 g).Yield: 128 mg (66%), mp: 217–219 °C, C_18_H_19_N_5_O_3_ (MW 353.38). ^1^H NMR (300 MHz, DMSO-*d*_6_) δ: 3.01 (t, *J* = 2.64 Hz, 1H, C≡CH), 3.32 (s, 3H, *N3*CH_3_), 3.58 (s, 3H, *N7*CH_3_), 3.72 (s, 3H, OCH_3_), 4.48–4.53 (m, 4H, *N1*CH_2_ +NHCH_2_), 6.77–6.83 (m, 1H, ph-4-H), 6.89–6.94 (m, 2H, phe-2,6-H), 7.18–7.26 (m, 1H, phe-5-H), 7.63 (t, *J* = 5.86 Hz, 1H, NHCH_2_); ^13^C NMR (DMSO-*d*_6_) δ: 29.8 (*N7*CH_3_), 29.9 (*N3*CH_3_), 30.3 (*N1*CH_2_), 46.1 (NHCH_2_), 55.4 (OCH_3_), 72.9 (C≡CH), 80.6 (C≡CH), 102.3 (xan-C5), 112.7 (phe-C2), 113.5 (phe-C4), 120.0 (phe-C6), 129.8 (phe-C5), 141.5 (phe-C1), 149.1 (xan-C4), 150.6 (xan-C2), 152.1 (xan-C6), 154.7 (xan-C8), 159.7 (phe-C3). UPLC-MS purity > 99%; *t*_R_ = 5.35; (ESI) *m*/*z* [M]^+^ 354.22.

*8-((3,4-dimethoxybenzyl)amino)-3,7-dimethyl-1-(prop-2-yn-1-yl)-3,7-dihydro-1H-purine-2,6-dione* (**26**)

Synthesis from **3H** (0.55 mmol; 0.157 g) and 3,4-dimethoxybenzylamine (1.1 mmol, 0.184 g). Yield: 152 mg (72%), mp: 247–249 °C, C_19_H_21_N_5_O_4_ (MW 383.41). ^1^H NMR (300 MHz, DMSO-*d*_6_) δ: 3.02 (t, *J* = 2.34 Hz, 1H, C≡CH), 3.33 (s, 3H, *N3*CH_3_), 3.57 (s, 3H, *N7*CH_3_), 3.70 (s, 3H, OCH_3_), 3.72 (s, 3H, OCH_3_), 4.44 (d, *J* = 5.86 Hz, 2H, NHCH_2_), 4.51 (d, *J* = 2.34 Hz, 2H, *N1*CH_2_), 6.87 (s, 2H, phe-5,6-H), 7.01 (s, 1H, phe-2-H), 7.56 (t, *J* = 5.86 Hz, 1H, NHCH_2_); ^13^C NMR (DMSO-*d*_6_) δ: 29.8 (*N7*CH_3_), 29.9 (*N3*CH_3_), 30.3 (*N1*CH_2_), 46.1 (NHCH_2_), 55.8 (OCH_3_), 56.0 (OCH_3_),72.9 (C≡CH), 80.6 (C≡CH), 102.3 (xan-C5), 112.1 (phe-C2), 116.5 (phe-C5), 120.1 (phe-C6), 132.3 (phe-C1), 148.3 (phe-C4), 149.0 (phe-C3), 149.1 (xan-C4), 150.6 (xan-C2), 152.1 (xan-C6), 154.7 (xan-C8). UPLC/MS purity 96.0%; *t*_R_ = 4.81; (ESI) *m*/*z* [M + H]^+^ 384.20.

*General procedure for the synthesis of 8-substitued benzylaminoxanthines:* **6**–**11**

The corresponding *N1*-substututed derivative of 8-bromothobromine (**3B**–**3E**) (1 eq) and 2-chlorobenzylamnie (2 eq) in methoxyethanol (3–10 mL) were refluxed for 15–17 h. Next, water was added and a solid was filtered off. A product was purified by column chromatography (CC; eluent:CH_2_Cl_2_:CH_3_OH from 98:12) or crystallized from ethanol. 

*1-Butyl-8-((2-chlorobenzyl)amino)-3,7-dimethyl-3,7-dihydro-1H-purine-2,6-dione* (**6**)

Synthesis from **3B** (0.8 mmol; 0.25 g) and 2-chlorobenzylamine (1.6 mmol, 0.23 g). Purified by CC. Yield: 60 mg (20%) mp: 181–183 °C, C_18_H_22_ClN_5_O_2_ (MW 375.86). ^1^H NMR (400 MHz, DMSO-*d*_6_) δ: 0.89 (t, *J* = 7.24 Hz, 3H, *N1*(CH_2_)_3_CH_3_), 1.22–1.32 (m, 2H, *N1*(CH_2_)_2_CH_2_), 1.44–1.53 (m, 2H, *N1*CH_2_CH_2_), 3.29 (s, 3H, *N3*CH_3_), 3.65 (s, 3H, *N7*CH_3_), 3.78–3.84 (m, 2H, *N1*CH_2_), 4.62 (d, *J* = 5.87 Hz, 2H, NHCH_2_), 7.27–7.36 (m, 2H, phe-5,6-H), 7.44–7.48 (m, 2H, phe-3,4-H), 7.62 (t, *J* = 5.87 Hz, 1H, NHCH_2_); ^13^C NMR (DMSO-*d*_6_) δ: 14.2 (*N1*CH_2_CH_2_CH_2_CH_3_), 20.1 (*N1*CH_2_CH_2_CH_2_), 29.7 (*N7*CH_3_), 30.3 (*N3*CH_3_), 30.3 (*N1*CH_2_CH_2_), 43.9 (NHCH_2_/*N1*CH_2_), 102.7 (xan-C5), 127.6 (phe-C5), 129.2 (phe-C4), 129.4 (phe-C6), 129.6 (phe-C3), 132.5 (phe-C2), 136.9 (phe-C1), 148.6 (xan-C4), 151.1 (xan-C2), 153.3 (xan-C6), 154.2 (xan-C8). UPLC-MS purity > 99%; *t*_R_ = 6.99; *m*/*z* [M + H]^+^ 376.15.

*8-((2-Chlorobenzyl)amino)-3,7-dimethyl-1-pentyl-3,7-dihydro-1H-purine-2,6-dione* (**7**)

Synthesis from **3C** (0.5 mmol; 0.16 g) and 2-chlorobenzylamine (1.0 mmol, 0.14 g). Purified by CC. Yield: 40 mg (21%) mp: 177–180 °C, C_19_H_24_ClN_5_O_2_ (MW 389.88). ^1^H NMR (500 MHz, DMSO-*d*_6_) δ: 0.81 (t, *J* = 7.09 Hz, 3H, *N1*(CH_2_)_4_CH_3_), 1.12–1.29 (m, 4H, *N1*CH_2_(CH_2_)_2_CH_2_CH_3_), 1.45 (quin, *J* = 7.09 Hz, 2H, *N1*(CH_2_)_3_CH_2_CH_3_), 3.24 (s, 3H, *N3*CH_3_), 3.60 (s, 3H, *N7*CH_3_), 3.75 (t, *J* = 7.09 Hz, 2H, *N1*CH_2_), 4.58 (d, *J* = 5.73 Hz, 2H, NHCH_2_), 7.21–7.33 (m, 2H, phe-3,6-H), 7.41 (dd, *J* = 7.30, 1.58 Hz, 2H, phe-4,5-H), 7.59 (t, *J* = 5.80 Hz, 1H, NHCH_2_). ^13^C NMR (DMSO-*d*_6_) δ: 14.3 (*N1*(CH_2_)_4_CH_3_), 22.3 (*N1*(CH_2_)_3_CH_2_), 27.8 (*N1*(CH_2_)_2_CH_2_), 29.0 (*N1*CH_2_CH_2_), 29.7 (*N7*CH_3_), 30.3 (*N3*CH_3_), 31.6 (*N1*CH_2_), 43.9 (NHCH_2_), 102.7 (xan-C5), 127.6 (phe-C5), 129.2 (phe-C6 + phe-C4), 129.6 (phe-C3), 132.5 (phe-C2), 136.9 (phe-C1), 148.6 (xan-C4), 151.1 (xan-C2), 153.3 (xan-C6), 154.2 (xan-C8). UPLC-MS^+^: Purity: > 99%, t_R_ = 7.55, (ESI) *m*/*z* [M + H]^+^ 390.18. 

*8-((2-Chlorobenzyl)amino)-1-hexyl-3,7-dimethyl-3,7-dihydro-1H-purine-2,6-dione* (**8**)

Synthesis from **3D** (0.5 mmol; 0.16 g) and 2-chlorobenzylamine (1.0 mmol, 0.343 g). Yield: 100 mg (25%), mp: 191–194 °C, C_20_H_26_ClN_5_O_2_ (MW 403.91). ^1^H NMR (400 MHz, DMSO-*d*_6_) δ: 0.83–0.88 (m, 3H, *N1*(CH_2_)_5_CH_3_), 1.26 (s, 6H, *N1*(CH_2_)_4_CH_2_ + *N1*(CH_2_)_3_CH_2_ + *N1*(CH_2_)_2_CH_2_), 1.45–1.53 (m, 2H, *N1*CH_2_CH_2_), 3.29 (s, 3H, *N3*CH_3_), 3.65 (s, 3H, *N7*CH_3_), 3.77–3.82 (m, 2H, *N1*CH_2_), 4.62 (d, *J* = 5.87 Hz, 2H, NHCH_2_), 7.28–7.36 (m, 2H, phe-5,6-H), 7.44–7.48 (m, 2H, phe-3,4-H), 7.62 (t, *J* = 5.87 Hz, 1H, NHCH_2_); ^13^C NMR (DMSO-*d*_6_) δ: 14.4 (*N1*(CH_2_)_5_CH_3_), 22.4 (*N1*(CH_2_)_4_CH_2_), 26.5 (*N1*CH_2_CH_2_CH_2_), 28.1 (*N1*CH_2_CH_2_), 29.7 (*N7*CH_3_), 30.3 (*N3*CH_3_), 31.5 (*N1*CH_2_CH_2_CH_2_), 43.9 (NHCH_2_ + N1CH_2_), 102.7 (xan-C5), 127.6 (phe-C5), 129.2 (phe-C4), 129.4 (phe-C6), 129.6 (phe-C3), 132.5 (phe-C2), 136.9 (phe-C1), 148.6 (xan-C4), 151.1 (xan-C2), 153.3 (xan-C6), 154.2 (xan-C8). UPLC-MS purity 99%; *t*_R_ = 8.10; (ESI) *m*/*z* [M]^+^ 404.20.

*1-Benzyl-8-((2-chlorobenzyl)amino)-3,7-dimethyl-1-propyl-3,7-dihydro-1H-purine-2,6-dione* (**9**)

Synthesis from **3E** (3 mmol; 1.05 g) and 2-chlorobenzylamine (6 mmol, 0.85 g). Yield: 620 mg (50%), mp: 205–208 °C, C_21_H_20_ClN_5_O_2_ (MW 409.87). ^1^H NMR (400 MHz, DMSO-*d*_6_) δ: 3.31 (s, 3H, *N3*CH_3_), 3.66 (s, 3H, *N7*CH_3_), 4.64 (d, *J* = 5.48 Hz, 2H, NHCH_2_), 5.01 (s, 2H, *N1*CH_2_), 7.19–7.37 (m, 7H, phe’-2,3,4,5,6-H + phe-5,6-H), 7.44–7.49 (m, 2H, phe-3,4-H), 7.68 (t, *J* = 5.87 Hz, 1H, NHCH_2_); ^13^C NMR (DMSO-*d*_6_) δ: 29.8 (*N7*CH_3_), 30.4 (*N3*CH_3_), 43.6 (*N1*CH_2_), 43.9 (NHCH_2_), 102.6 (xan-C5), 127.3 (phe’-C4′), 127.6 (phe-C5), 127.9 (phe’-C2′,C6′), 128.6 (phe’-C3′,C5′), 129.2 (phe-C4), 129.4 (phe-C6), 129.6 (phe-C3), 132.6 (phe-C2), 136.9 (phe-C1), 138.6 (phe’-C1′), 148.9 (xan-C4), 151.3 (xan-C2), 153.2 (xan-C6), 154.4 (xan-C8). UPLC-MS purity 98.7%, *t*_R_ = 7.12, (ESI) *m*/*z* [M]^+^ 410.25.

*1-(4-Chlorobenzyl)-8-((2-chlorobenzyl)amino)-3,7-dimethyl-3,7-dihydro-1H-purine-2,6-dione* (**10**)

Synthesis from **3F** (3 mmol; 1.15 g) and 2-chlorobenzylamine (6 mmol, 0.85 g). Yield: 50 mg (4%), mp: 228–230 °C, C_21_H_19_Cl_2_N_5_O_2_ (MW 444.32). ^1^H NMR (400 MHz, DMSO-*d*_6_) δ: 3.31 (s, 3H, *N3*CH_3_), 3.65 (s, 3H, *N7*CH_3_), 4.64 (d, *J* = 5.87 Hz, 2H, NHCH_2_), 4.99 (s, 2H, *N1*CH_2_), 7.28–7.36 (m, 6H, phe-5,6-H + phe’-2,3-5,6-H), 7.44–7.48 (m, 2H, phe-3,4-H), 7.69 (t, *J* = 5.87 Hz, 1H, NHCH_2_); ^13^C NMR (DMSO-*d*_6_) δ: 29.8 (*N7*CH_3_), 30.4 (*N3*CH_3_), 43.0 (*N1*CH_2_), 43.9 (NHCH_2_), 102.7 (xan-C5), 127.6 (phe-C5), 128.6 (phe’-C3′,C5′), 129.2 (phe-C4), 129.4 (phe-C6), 129.6 (phe-C3), 129.9 (phe’-C2′,C6′), 131.9 (phe’-C1′), 132.6 (phe-C2), 136.9 (phe-C1), 137.6 (phe’-C4′), 149.0 (xan-C4), 151.3 (xan-C2), 153.1 (xan-C6), 154.5 (xan-C8). UPLC-MS purity 98.7%; *t*_R_ = 7.77, (ESI) *m*/*z* [M]^+^ 444.21.

*8-((2-Chlorobenzyl)amino)-1-(3,4-dichlorobenzyl)-3,7-dimethyl-3,7-dihydro-1H-purine-2,6-dione* (**11**)

Synthesis from **3G** (3 mmol; 1.25 g) and 2-chlorobenzylamine (6 mmol, 0.85 g). Yield: 15% (220 mg), mp: 198–203 °C, C_21_H_18_Cl_3_N_5_O_2_ (MW 477.05). ^1^H NMR (400 MHz, DMSO-*d*_6_) δ: 3.30 (s, 3H, *N3*CH_3_), 3.65 (s, 3H, *N7*CH_3_), 4.64 (d, *J*= 5.87 Hz, 2H, NHCH_2_), 4.98 (s, 2H, N1CH_2_), 7.24–7.36 (m, 3H, Phe-5,6-H + phe’-6′-H), 7.44–7.49 (m, 2H, phe-3,4-H), 7.51–7.56 (m, 2H, phe’-2′,5′-H), 7.70 (t, *J* = 5.87 Hz, 1H, NHCH_2_); ^13^C NMR (DMSO-*d*_6_) δ: 29.9 (N7CH_3_), 30.4 (N3CH_3_), 42.8 (N1CH_2_), 43.9 (NHCH_2_), 102.7 (xan-C5), 127.6 (phe-C5), 128.4 (phe’-C6′), 129.2 (phe-C4), 129.4 (phe-C6), 129.6 (phe-C3), 130.0 (phe’-C1′), 130.1 (phe’-C3′), 130.9 (phe’-C5′), 131.2 (phe-C4′),132.6 (phe-C2), 136.9 (phe-C1, phe), 139.8 (phe’-C1), 149.1 (xan-C4), 151.3 (xan-C2), 153.0 (xan-C6), 154.5 (xan-C8). UPLC-MS purity > 99%, *t*_R_ = 8.35, (ESI) *m*/*z* [M]^+^ 478.11.

#### 4.1.3. Synthesis of 8-substitued 3-cyclopropyl-7-methyl-1-propargylxanthines **27**–**29**

##### Synthesis of Substrates

*Synthesis of cyclopropylurea* (**CP-1**)

To a mixture of cyclopropylamine (0.13 mol) in 26 mL of 5 N HCl was added KOCN (0.13 mol) and then heated for 4 h at 70 °C. Next, the reaction mixture was evaporated to dryness and ethanol was added. The precipitated KCl was filtered off and the filtrate was concentrated on an evaporator and petroleum ether was added. The solid was then filtered and washed with petroleum ether, and recrystallized with acetone. Yield 41% (5.2 g), mp: 115–117 °C, C_4_H_8_N_2_O (MW 100.12). UPLC-MS: Purity 92.9%, *t*_R_ = 1.26, (ESI) *m*/*z* [M]^+^ 106.96. 

*Synthesis of 6-amino-1-cyclopropyl uracil* (**CP-2**)

A mixture of cyclopropylurea (**CP-1**) (0.045 mol), cyanoacetic acid ethyl ester (0.045 mol) and 67 mL of sodium ethanolate was heated for 4 h at 100 °C. Then, the solvent was evaporated and water was added to the residue. The resulting solution was adjusted to pH–7 with acetic acid. The precipitate was filtered and washed with acetone. Yield: 27% (2.1 g), mp: 246–248 °C, C_7_H_10_N_3_O_2_ (MW 168.17). UPLC-MS: Purity > 99%, *t*_R_ = 1.46, (ESI) *m*/*z* [M]^+^ 169.07. 

*Synthesis of 6-amino-1-cyclopropyl-5-nitrosouracil* (**CP-3**)

A mixture of 6-amino-1-cyclopropylouracil (**CP-3**) (0.02 mol) in 45 mL of 50% acetic acid was heated until completely dissolved at 60 °C. Then, 1.2 g NaNO_2_ was gradually added until brown vapors formed and the violet precipitate was formed. The solid was filtered off, washed with water, dried and directly used for the next step. Yield: 48% (1.9 g), C_7_H_8_N_4_O_3_ (MW 196.16).

*Synthesis of 1-cyclopropyl-5,6-diaminouracil* (**CP-4**)

A mixture of 6-amino-1-cyclopropyl-5-nitrosouracil (**CP-3**) (0.01 mol) in 60 mL of 12.5% NH_3_ was heated at 70 °C until a clear solution was obtained. At this temperature, about 4.2 g of sodium dithionite was gradually added over a period of 10 min until the color changed from red to yellow. The resulting solution was concentrated until the product began to crystallize, then cooled to 4 °C. The product was filtered off, washed with water and directly used in the next reaction. Yield: 80% (1.4 g), C_7_H_10_N_4_O_2_ (MW 182.18).

*Synthesis of 3-cyclopropyl-3,7-dihydro-1H-purine-2,6-dione* (**CP-5**)

A mixture of 1-cyclopropyl-5,6-diaminouracil (**CP-4**) (0.01 mol) and 15 mL of a 95–97% solution of formic acid was refluxed for 1 h. Then, the excess formic acid was distilled off and the formed precipitate was directly cyclized by adding 15 mL of a 10% sodium hydroxide solution to alkalize the reaction medium. The mixture was then refluxed for 2 h, cooled and acidified with 10% HCl to pH = 5. The precipitated xanthine was filtered off and dried. Yield: 65% (1.24 g), C_8_H_8_N_4_O_2_ (MW 192.18).

*Synthesis of 8-bromo-3-cyclopropyl-3,7-dihydro-1H-purine-2,6-dione* (**CP-6**)

A mixture of 3-cyclopropylxanthine (**CP-5**) (8 mmol), 99.5% acetic acid (8.8 mL) and 40% HBr (1.22 mL) was heated in a water bath at 58 °C until a clear solution was obtained. Next, NaClO_3_ (0.4 g dissolved in 2.50 mL water) was slowly dropped. The resulting mixture was heated for 2 h and the precipitate was filtered, washed with water and dried. Yield: 62% (1.35 g), C_8_H_7_BrN_4_O_2_ (MW 271.07).

*Synthesis of 8-bromo-3-cyclopropyl-7-methyl-3,7-dihydro-1H-purine-2,6-dione* (**CP-7**)

A mixture of 8-bromo-3-cyclopropylxanthine (**CP-6**) (4 mmol), iodomethane (4.8 mmol) and DIPEA (8 mmol) in 8 mL of DMF was heated at 40 °C for 4 h. Then, water was added and the resulting precipitate was filtered, washed with water, dried and directly used for the next step of the synthesis. C_9_H_9_BrN_4_O_2_ (MW 285.10).

*Synthesis of 8-bromo-3-cyclopropyl-7-methyl-1-(prop-2-yn-1-yl)-3,7-dihydro-1H-purine-2,6-dione* (**CP-8**)

A mixture of 8-bromo-3-cyclopropylo-7-methyl-3,7-dihydro-1H-purine-2,6-dione (**CP-7**) (4 mmol), propargyl bromide (8 mmol) and K_2_CO_3_ (8 mmol) in 4 mL of DMF was heated at 75 °C for 4 h. Then, water was added and the resulting precipitate was filtered, washed with water, dried and directly used for the next step of the synthesis. C_12_H_11_BrN_4_O_2_ (MW 323.15).

##### General Procedure of Synthesis of Compounds **27**–**29**

A mixture of 8-bromo-3-cyclopropyl-7-methyl-1-(prop-2-yn-1-yl)-3,7-dihydro-1H-purine-2,6-dione (**CP-8**) (0.55 mmol, 0.178 g), an appropriate benzylamine (1.1 mmol), TEA (1.6 mmol) and 1.00 mL of n-propanol was heated in closed vessels in a microwave oven (CEM Discover SC, 300 Watt, Power Max Off, 150 °C, 10 bar) for 1 h. Then, the solvent was removed and the residue was treated with CH_2_Cl_2_. The products were purified by crystallization from ethanol or flash column chromatography over silica gel with CH_2_Cl_2_:MeOH (100:0 to 80:20).

*8-((2-Chlorobenzyl)amino)-3-cyclopropyl-7-methyl-1-(prop-2-yn-1-yl)-3,7-dihydro-1H-purine-2,6-dione* (**27**)

Synthesis from 2-chlorobenzylamine (1.1 mmol, 0.156 g). Yield: 46% (97 mg), mp: 172–173 °C, C_19_H_18_ClN_5_O_2_ (MW 383.84). ^1^H NMR (300 MHz, DMSO-*d*_6_) δ: 0.78–0.87 (m, 2H, cyclopropyl-2,3-H), 0.87–0.96 (m, 2H, cyclopropyl-2,3-H), 2.86 (tt, *J* = 7.03, 3.52 Hz, 1H, cyclopropyl-1-H), 3.00 (s, 1H, C≡CH), 3.60 (s, 3H, *N7*CH_3_), 4.49 (d, *J* = 2.34 Hz, 2H, *N1*CH_2_), 4.60 (d, *J* = 5.86 Hz, 2H, NHCH_2_), 7.24–7.33 (m, 2H, phe-5,6-H), 7.40–7.50 (m, 2H, phe-3,4-H), 7.64 (t, *J* = 5.57 Hz, 1H, NHCH_2_); ^13^C NMR (DMSO-*d*_6_) δ: 8.0 (cyclopropyl-C2,C3), 26.5 (*N3*CH_2_), 29.9 (*N7*CH_3_), 30.2 (*N1*CH_2_), 44.0 (NHCH_2_), 72.8 (C≡CH), 80.6 (C≡CH), 102.8 (xan-C5), 127.5 (phe-C5), 129.1 (phe-C4), 129.5 (phe-C6), 129.9 (phe-C3), 132.7 (phe-C2), 137.0 (phe-C1), 149.5 (xan-C4), 151.1 (xan-C2), 152.4 (xan-C6), 154.0 (xan-C8). UPLC-MS purity 93.19%, *t*_R_ = 6.25, *m*/*z* [M]^+^ 383.93.

*3-Cyclopropyl-8-((2-fluorobenzyl)amino)-7-methyl-1-(prop-2-yn-1-yl)-3,7-dihydro-1H-purine-2,6-dione* (**28**)

Synthesis from 2-fluorobenzylamine (1.1 mmol, 0.138 g). Yield: 48% (97 mg), mp: 190–192 °C, C_19_H_18_FN_5_O_2_ (MW 367.38). ^1^H NMR (300 MHz, DMSO-*d*_6_) δ: 0.80–0.99 (m, 4H, cyclopropyl-2,3-H), 2.88 (tt, *J* = 7.11, 3.74 Hz, 1H, cyclopropyl-1-H,), 2.99 (s, 1H, C≡CH), 3.57 (s, 3H, *N7*CH_3_), 4.49 (d, *J* = 2.34 Hz, 2H, *N1*CH_2_), 4.55 (d, *J* = 5.28 Hz, 2H, NHCH_2_), 7.11–7.20 (m, 2H, phe-5,6-H), 7.25–7.34 (m, 1H, phe-3-H), 7.48 (t, *J* = 7.62 Hz, 1H, phe-4-H), 7.62 (t, *J* = 5.57 Hz, 1H, NHCH_2_); ^13^C NMR (DMSO-*d*_6_) δ: 8.0 (cyclopropyl-C2,C3), 26.5 (*N3*CH_2_), 29.9 (*N7*CH_3_), 30.2 (*N1*CH_2_), 72.8 (C≡CH), 80.6 (C≡CH), 102.8 (xan-C5), 115.5 (d, ^2^*J*_C,F_ = 20.7 Hz, phe-C3), 124.3 (d, ^4^*J*_C,F_ = 3.4 Hz, phe-C5), 126.7 (d, ^2^*J*_C,F_ = 15.0 Hz, phe-C4), 129.4 (d, ^2^*J*_C,F_ = 8.1 Hz, phe-C1), 130.6 (d, ^3^*J*_C,F_ = 3.4 Hz, phe-C6), 149.5 (xan-C4), 151.1 (xan-C2), 152.4 (xan-C6), 154.1 (xan-C8), 160.7 (d, ^1^*J*_C,F_ = 244.1 Hz, phe-C2). UPLC-MS purity 97.5%, *t*_R_ = 5.77, *m*/*z* [M]^+^ 368.04.

*8-((2-Chloro-6-fluorobenzyl)amino)-3-cyclopropyl-7-methyl-1-(prop-2-yn-1-yl)-3,7-dihydro-1H-purine-2,6-dione* (**29**)

Synthesis from 2-chloro-6-fluorobenzylamine (1.1 mmol, 0.176 g). Yield: 44% (97 mg), mp: 186–187 °C, C_19_H_17_ClFN_5_O_2_ (MW 401.83). ^1^H NMR (300 MHz, DMSO-*d*_6_) δ: 0.83–1.02 (m, 4 H, C2H_2_, C3H_2_, cyclopropyl), 2.84–2.93 (m, 1 H, C1H, cyclopropyl), 3.00 (t, *J* = 2.34 Hz, 1H, C≡CH), 3.52 (s, 3H, *N7*CH_3_), 4.49 (d, *J* = 2.34 Hz, 2H, *N1*CH_2_), 4.63 (d, *J* = 4.10 Hz, 2H, NHCH_2_), 7.17–7.26 (m, 1H, phe-4-H), 7.28–7.44 (m, 3H, phe-3,5-H + NHCH_2_); ^13^C NMR (DMSO-*d*_6_) δ: 8.0 (cyclopropyl-C2,C3,), 26.5 (*N3*CH_2_), 29.9 (*N7*CH_3_), 30.3 (*N1*CH_2_), 38.4 (d, ^3^*J*_C,F_ = 4.6 Hz, NHCH_2_), 72.8 (C≡CH), 80.6 (C≡CH), 102.7 (xan-C5), 114.9 (d, ^2^*J*_C,F_ = 23.1 Hz, phe-C5), 124.4 (d, ^2^*J*_C,F_ = 17.3 Hz, phe-C1), 125.9 (d, ^4^*J*_C,F_ = 3.4 Hz, phe-C3), 130.6 (d, ^3^*J*_C,F_ = 9.2 Hz, phe-C4), 135.4 (d, ^3^*J*_C,F_ = 5.7 Hz, phe-C2), 149.5 (xan-C4), 152.2 (xan-C2), 152.5 (xan-C6), 153.7 (xan-C8), 162.0 (d, ^1^*J*_C,F_ = 248.8 Hz, phe-C6). UPLC-MS purity 96.5%, *t*_R_ = 6.20, *m*/*z* [M]^+^ 402.21.

### 4.2. Radioligand Binding Assays at Human Adenosine Receptors

Affinities for adenosine receptors were evaluated in radioligand binding assays as previously described [13,32]. Human adenosine receptors were stably expressed in CHO cells. The following radioligands were used: [^3^H]2-chloro-N^6^-cyclopentyladenosine ([^3^H]CCPA) for A_1_R; [^3^H]3-(3-hydroxypropyl)-7-methyl-8-(*m*-methoxystyryl)-1-propargylxanthine ([^3^H]MSX-2) for A_2A_R; [^3^H]8-(4-(4-(4-chlorophenyl)piperazine-1-sulfonyl)phenyl)-1-propylxanthine ([^3^H]PSB-603) for A_2B_R; [^3^H]phenyl-8-ethyl-4-methyl-(8*R*)-4,5,7,8-tetrahydro-1*H*-imidazo [2,1-*i*]purine-5-one ([^3^H]PSB-11) for A_3_R. Initially, compounds were tested at a concentration of 1 µM. In cases where the percent inhibition was > 50, full concentration-inhibition curves were determined to calculate K_i_ values. At least three independent experiments were performed. Data were analyzed using GraphPad PRISM version 5.0 or higher (Graph Pad, San Diego, CA, USA). K_i_ were calculated using the Cheng–Prusoff equation.

### 4.3. Human MAO B Inhibitory Activity

The compounds were tested twice in duplicates at a concentration of 1 μM by fluorometric method as previously described [13]. Paratyramine (200 μM) was used as a substrate for the enzyme. Safinamide (1 μM; reversible) and rasagiline (1 μM; irreversible) were used as reference inhibitors.

### 4.4. Molecular Modeling Studies to Adenosine A_1_ and A_2A_ Receptors

For docking studies, Schrodinger 2022-4 [33] was used. Bioactive conformations were generated using ConfGen [34] (water environment, target number of conformers—20). For all the compounds, the five lowest energy conformers were selected for docking studies. Docking to a rigid form of the receptor (bound ligand-centered grid) was performed using a standard docking protocol [35]. To validate the methods used, the ligand present in the protein structures 5N2S and 5N2R (adenosine A_1_R and A_2A_R, respectively) [14] was redocked with high confidence. The binding free energy of the docked pose was calculated using Prime MM-GBSA [36]. Proteins were prepared with Protein Preparation Workflow using default settings. OPLS4 forcefield was used for calculations.

Dynamics simulations (for 150 ns, T = 300 K) were run in Desmond [37]. The protein orientation in the membrane was obtained from the OPM database [38]. The simulation lasted for 150 ns and the POPC (300 K) membrane / TIP3P solvent model was applied [39]. A total of 1000 frames were produced for the run. The obtained trajectories were then visually analyzed, as well as using the Simulation Interaction Analysis tool of Desmond/Maestro. All of the figures are derived from the Schrödinger package and were prepared using freely available graphics software. 

### 4.5. ADMET Properties

#### 4.5.1. Toxicity Evaluation

To evaluate neurotoxicity and hepatotoxicity, the human neuroblastoma cell line SH-SY5Y (ATCC^®^ no. CRL-2266™) and hepatoma cell line HepG2 (ATCC^®^ no. HB-8065™) were used, respectively. The cells (SH-SY5Y: 5 × 10^3^ cells/100μL/well and HepG2: 7 × 10^3^ cells/100 μL/well) were seeded in transparent 96-well plates (Nunc) in DMEM supplemented with 10% FBS and cultured overnight. The next day, the medium was changed to medium containing dimethylsulfoxide (DMSO < 0.1%, vehicle control) or an increasing concentration of compounds MZ-1483 (**19**), MZ-1490 (**22**) and MZ-1495 (**24**) (9.8 × 10^−6^–100 × 10^−6^ M). To perform dose-response analysis, two-fold serial dilutions (11 points) were prepared. Treatment with compounds was performed for 48 h. After the incubation time, the cell viability was examined using an MTS-based [3-(4,5-dimethylthiazol-2-yl)-5-(3-carboxymethoxyphenyl)-2-(4-sulfophenyl)-2 H tetrazolium] CellTiter96^®^ AQueous One Solution Cell Proliferation Assay (Promega, Madison, WI, USA) following the manufacturer’s protocol. Briefly, 20 μL of MTS solution was pipetted into each well containing 100 μL of culture or culture medium (negative control) and incubated at 37 °C for 1 h. After incubation time, formazan product turnover absorbance was measured at 490 nm using the microplate reader (Tecan Spark^®^, Tecan Group Ltd., Maennedorf, Switzerland). A reference wavelength of 630 nm was used.

#### 4.5.2. Metabolic Stability in Human Liver Microsomes

Human liver microsomes (HLMs) were purchased from Sigma-Aldrich (St. Louis, MO, USA; catalog nr M9066-1VL). The studies with microsomes were supported by MetaSite 6.0.1 software provided by Molecular Discovery Ltd. (Hertfordshire, UK), which allowed for the determination of the most probable sites of metabolism. The compounds were incubated with HLMs for 120 min. After the addition of methanol and centrifugation, the supernatants were analyzed with the Waters ACQUITY™ TQD system (Waters, Milford, CT, USA). All reference drugs used (caffeine, ketoconazole, quinidine and sulfaphenazole) were purchased from Sigma-Aldrich (St. Louis, MO, USA).

#### 4.5.3. Drug—Drug Interactions

The influence of the tested compounds on recombinant human cytochromes was determined with the use of CYP3A4, CYP2D6 and CYP2C9 P450-Glo™ kits and protocols provided by Promega (Madison, WI, USA). Compounds were tested at a concentration of 10 μM. Reference inhibitors: Ketoconazole (CYP3A4), quinidine (CYP2D6) and sulfaphenazole (CYP2C9) were tested at 1 μM. The luminescent signals were measured using a microplate reader EnSpire PerkinElmer (Waltham, MA, USA).

#### 4.5.4. Blood Brain Barrier Permeability

For permeability evaluation, a PAMPA Gentest™ pre-coated platelet system, provided by Corning (Tewksbury, MA, USA), was used. The precise procedure was previously described [40]. The permeability coefficient *P_e_* was calculated based on the given equations. Concentrations of the tested compounds in the apical and basolateral wells were determined by LC/MS analysis using an internal standard. The results were compared with a high-permeability reference compound caffeine (CFN).

### 4.6. Anti-Inflammatory Activity In Vitro

#### 4.6.1. Preliminary Screening for Anti-Inflammatory Activity (Griess Assay)

Griess reagent (Sigma-Aldrich, Saint Louis, MO, USA, G4410) was freshly prepared by dissolving 1 g in ultrapure water and mixing by inversion for about 4 min under protection from light. After 24 h of incubation, 100 μL of culture medium was transferred to a transparent 96-well plate and mixed with the same volume of Griess reagent. The plate was incubated for 15 min at room temperature and the absorbance was read at 540 nm using the EnSpire microplate reader (PerkinElmer, Waltham, MA, USA). Data were analyzed by direct comparison based on the average absorbance of LPS treatment, compound treatment or co-treatment with LPS, performed by normalizing treated cells (compound alone or co-treatment with LPS) to LPS-treated cells set at 100%.

#### 4.6.2. Phagocytic Activity

Phagocytosis was monitored in real time using the IncuCyte^®^ pHrodo^®^ Red Cell Labeling Kit. The principle of this test is based on the ability of the pHrodo to emit fluorescence in an acidic environment, which is precisely the environment inside the phagosome. If effector cells engulf labeled target cells and these enter the acidic phagosome, a substantial increase in fluorescence is observed. In this way, we can quantify phagocytosis. In our experiment, Jurkat cells were used as target cells. In order to induce apoptosis in these cells, staurosporine was applied at a final concentration of 5 μM for 24 h. Following this, apoptotic Jurkat cells were labeled with IncuCyte pHrodo at 250 ng/mL for 1 h. According to the manufacturer’s instructions, the following steps were taken. BV-2 cells were used as effector cells in this study. One day prior to the experiment, BV-2 cells were seeded in 96-well plates at a density of 1 × 10^4^ cells/well/100 μL. The next day, BV-2 cells were treated for 1 h according to the scheme to create four experimental groups: (1) 0.1% DMSO—Vehicle; (2) LPS 1 μg/mL; (3) MZ-1490 (**22**) at 1 μM; (4) MZ-1490 (**22**) at 1 μM + LPS 1 μg/mL. After this time, the labeled Jurkat apoptotic cells were added to the BV-2 cells at a density of 100,000 cells/well and the cells were monitored in an IncuCyte Live-Cell Analysis System. Repeat scanning to record phase and fluorescence images was carried out every 1 h, for up to 22 h. Objective 10× and 800 ms acquisition was applied. 

### 4.7. Antinociceptive and Anti-Inflammatory Activity In Vivo

#### 4.7.1. Animals

The in vivo experiments were carried out on male albino Swiss mice weighing 18–26 g and male Wistar rats weighing 150–180 g. The animals were housed in constant temperature facilities exposed to a 12:12 light-dark cycle and maintained on a standard pellet diet and tap water given ad libitum. The control and experimental groups consisted of 6–8 animals each. The investigated compounds were *intraperitoneally* (*ip*) administered in the form of a suspension in 0.5% methylcellulose. Control animals received the equivalent volume of solvent. All procedures were conducted according to the guidelines of ICLAS (International Council on Laboratory Animals Science) and were approved by the Local Ethics Committee of the Jagiellonian University in Kraków.

#### 4.7.2. Statistical Analysis 

The data are expressed as the mean ± SEM (standard error of the mean). Differences between vehicle control and treatment groups were tested using two-way ANOVA followed by Bonferroni multiple comparison test. The difference in means was statistically significant if *p* < 0.05. 

#### 4.7.3. The Formalin Test

The mice were pretreated with the test compound or the vehicle and were allowed to acclimate in Plexiglas observation chambers (20 × 30 × 15 cm) for 30 min before the test. Then, 20 μL of a 5% formalin solution was intraplantarly injected into the right hind paw using a 26-gauge needle. Immediately after formalin injection, the animals were individually placed into glass beakers and were observed during the next 30 min. Time (in seconds) spent on licking or biting the injected paw in selected intervals, 0–5, 15–20, 20–25 and 25–30 min, was measured in each experimental group and was an indicator of nociceptive behavior [41]. The ED_50_ values and their confidence limits were estimated by the method of Litchfield and Wilcoxon [42]. 

#### 4.7.4. Carrageenan-Induced Edema Model 

Rats were divided into four groups, one of them being the control. In order to produce inflammation, 0.1 mL of 1% carrageenan solution in water was injected into the hind paw subplantar tissue of rats, according to the modified method of C. A. Winter [25] and P. Lence [43]. The development of paw edema was measured with a plethysmometr (Plethysmometr 7140, Ugo Basile). Prior to the administration of test substances, paw diameters were measured by dividers and recorded. The investigated compounds were administered at a dose of 20 mg/kg, *ip* (as a suspension in methylcellulose), prior to carrageenan injection. Methylcellulose was administered by the same route to the control group (methylcelullose had no effect on edema, data not shown). After these administrations, paw diameters were measured at 1, 2 and 3 h. The edema % and edema inhibition % were calculated according to the formulas given below.

Edema% = (N′ × 100)/N

Edema inhibition% = (N – N′ × 100)/N

N: Paw diameters measured 1, 2 and 3 h after injection of carrageenan to the control group—paw diameters at the beginning. 

N′: Paw diameters measured 1, 2 and 3 h after injection of carrageenan to the test groups—paw diameters at the beginning. 

## Figures and Tables

**Table 1:** Affinities of xanthine derivatives **5**–**29** for human adenosine receptor subtypes ^a^.

Compd.	Structure	A_1_R vs. [³H]CCPA ^b^	A_2A_R vs. [³H]MSX-2 ^c^	A_2B_R vs. [³H]PSB-603 ^d^	A_3_R vs. [H³]PSB-11 ^e^
		*K*_i_ ± SEM (nM) (or % Inhibition ± SEM at 1 µM)			
Series IA—*N1*-Different Substituents					
Compound **2** **(lead)**	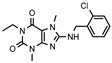	>1000 ^f^ (22 ± 1)	71 ± 6 ^f^	>1000 ^f^ (44 ± 10)	>1000 ^f^ (14 ± 1)
**5**	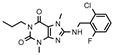	>1000 (26 ± 8)	302 ± 34	832 ± 133	>1000 (4 ± 4)
**6**	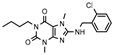	>1000 (21 ± 6)	318 ± 141	>1000 (32 ± 0)	>1000 (11 ± 2)
**7**	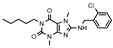	>1000 (35 ± 2)	>1000 (38 ± 4)	>1000 (41 ± 8)	>1000 (13 ± 3)
**8**	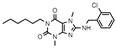	>1000 (17 ± 6)	>1000 (9 ± 8)	>1000 (13 ± 5)	>1000 (31 ± 3)
**9**	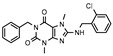	>1000 (23 ± 5)	323 ± 150	>1000 (9 ± 1)	>1000 (24 ± 7)
**10**	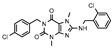	>1000 (12 ± 1)	>1000	>1000 (14 ± 11)	>1000 (31 ± 2)
**11**	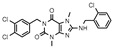	>1000 (10 ± 2)	>1000 (23 ± 7)	>1000 (4 ± 7)	>1000 (40 ± 9)
**Series IB—*N1*-propyl derivatives**					
**12**	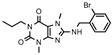	>1000 (30 ± 1)	361 ± 82	>1000 (33 ± 10)	>1000 (8 ± 4)
**13**	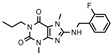	>1000 (32 ± 4)	>1000 (47 ± 2)	>1000 (21 ± 11)	>1000 (6 ± 8)
**14**	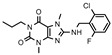	>1000 (29 ± 10)	124± 26	687 ± 147	>1000 (18 ± 4)
**15**	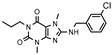	>1000 (40 ± 3)	588 ± 74	>1000 (34 ± 8)	>1000 (0 ± 1)
**16**	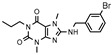	340 ± 73	254 ± 59	>1000 (30 ± 1)	>1000 (11 ± 11)
**17**	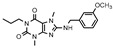	>1000 (17 ± 2)	605 ± 120	>1000 (20 ± 1)	>1000 (6 ± 3)
**18**	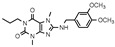	>1000 (7 ± 12)	773 ± 127	>1000 (17 ± 9)	>1000 (−5 ± 10)
**Series IC—*N1*-propargyl derivatives**					
**19** **(MZ-1483)**	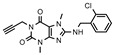	407 ± 75	96 ± 31	>1000 (14 ± 6)	>1000 (9 ± 1)
**20**	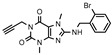	>1000 (44 ± 5)	137 ± 35	>1000 (25 ± 10)	>1000 (13 ± 9)
**21**	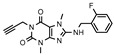	353 ± 42	287 ± 90	>1000 (41 ± 3)	>1000 (21 ± 1)
**22** **(MZ-1490)**	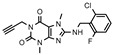	130 ± 34	62 ± 5	>1000 (41 ± 6)	>1000 (21 ± 6)
**23**	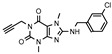	334 ± 122	126± 15	>1000 (26 ± 2)	>1000 (14 ± 4)
**24** **(MZ-1495)**	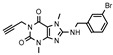	72 ± 8	77 ± 24	>1000 (25 ± 8)	>1000 (7 ± 8)
**25**	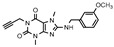	460 ± 99	225 ± 40	>1000 (35 ± 9)	>1000 (0 ± 2)
**26**	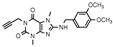	>1000 (−2 ± 7)	108 ± 22	>1000 (28 ± 6)	>1000 (2 ± 0)
**Series II—*N1*-propargyl-*N3*-cyclopropyl derivatives**					
**27**	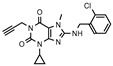	247 ± 43	311 ± 167	>1000 (9 ± 1)	>1000 (16 ± 2)
**28**	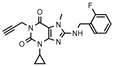	166 ± 65	386 ± 61	>1000 (14 ± 4)	>1000 (−2 ± 6)
**29**	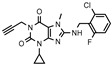	78 ± 9	204 ± 38	>1000 (16 ± 3)	>1000 (16 ± 1)

^a^ Human recombinant receptors expressed in CHO cells. K_i_ values were determined as means from three independent experiments ± standard error of the mean (SEM); ^b^ [³H]2-Chloro-N^6^-cyclopentyladenosine; ^c^ [³H] (3-(3-Hydroxypropyl)-7-methyl-8-(*m*-methoxystyryl)-1-propargylxanthine); ^d^ [³H]8-(4-(4-(4-hClorophenyl)piperazine-1-sulfonyl)phenyl)-1-propylxanthine; ^e^ [^3^H]8-Ethyl-4-methyl-2-phenyl-(8*R*)-4,5,7,8-tetrahydro-1*H*-imidazo [2,1-*i*]-purin-5-one; ^f^ data from [13].

**Table 2:** Inhibitory activity of compounds **5**, **12**–**26** at human MAO B evaluated in a fluorescence assay.

Compound	% of hMAO B Inhibition at 1 µM ^a^	Compound	% of hMAO B Inhibition at 1 µM ^a^
**5**	8	**19**	6
**12**	7	**20**	32
**13**	15	**21**	8
**14**	9	**22**	7
**15**	10	**23**	8
**16**	11	**24**	10
**17**	5	**25**	11
**18**	2	**26**	3

^a^ Mean value of two independent experiments.

**Table 3:** The most probable metabolic pathways for compounds **19**, **22** and **24**, and the reference drug verapamil determined in vitro using human liver microsomes.

Drug	% Remaining in the Reaction Mixture ^a^	Molecular Mass (*m*/*z*)	Molecular Mass of the Metabolite (*m*/*z*)/ Retention Time (min)	Metabolic Pathway
**19**	>95	358.16	249.07/ 6.27 (M1)	fragmentation and hydroxylation
			234.16/ 3.59 (M2)	fragmentation, demethylation and hydroxylation
**22**	>90	376.12	249.53/ 6.26 (M1)	fragmentation, and hydroxylation
**24**	>95	402.13	249.13/ 6.26 (M1)	fragmentation, and hydroxylation
Verapamil ^b^	31	455.31	441.35/ 4.95 (M1)	demethylation
			291.32/ 4.00 (M2)	fragmentation
			165.09/ 3.38 (M3)	fragmentation
			441.29/ 4.62 (M4)	demethylation
			427.33/4.54 (M5)	double-demethylation
			277.26/ 3.92 (M6)	fragmentation

^a^ after 120 min of incubation with HLMs; ^b^ reference drug (low metabolic stability).

**Table 4:** Permeability coefficient of compounds (**19**, **22** and **24**) and caffeine.

Compound	^a,b^ *P_e_* (10^−6^ cm/s) ± SD	Permeable	Drug Retention (%)
Caffeine	6.57 ± 1.57	yes	29.46
**19**	3.13 ± 0.82	yes	51.78
**22**	0.37 ± 0.01	no	68.62
**24**	0.89 ± 0.71	no	93.30

^a^ PAMPA plate’s manufacturer breakpoint for permeable compounds: P_e_ ≥ 1.5. ^b^ Tested in triplicate.

**Table 5:** Anti-inflammatory (antiedematous) effect of the compounds in the carrageenan-induced paw edema test in rats.

Treatment	Dose (mg/kg)	Change in Edema Volume [mL]		
		1 h	2 h	3 h
Control		0.78 ± 0.03	1.12 ± 0.13	1.33 ± 0.13
**19**	20	0.56 ± 0.13	0.72 ± 0.09	1.10 ± 0.21
**22**	20	0.49 ± 0.08	0.91 ± 0.05	0.92 ± 0.06
**24**	20	0.38 ± 0.02 ^a^	0.63 ± 0.14 ^a^	0.69 ± 0.01 ^b^
**ketoprofen**	20	0.32 ± 0.03 ^a^	0.41 ± 0.04 ^a^	0.43 ± 0.03 ^b^

Data are presented as the means ± SEM of six to eight animals per group, ^a^ *p* < 0.05, ^b^ *p* < 0.01.

**Table 6:** The influence of the investigated compounds on the duration of pain reaction in the formalin test in mice ^a^.

Treatment	Dose (mg/kg)	Licking of the Hind Paw (sec)			
		Early Phase (0–5 min)	Inhibition (%)	Late Phase (15–30 min)	Inhibition (%)
		Mean ± SEM		Mean ± SEM	
Control		41.6 ± 15.2	-	119.4 ± 32.8	-
**19**	40	42.6 ± 2.8	0	63.0 ± 6.6 ^b^	47.2
	20	48.2 ± 10.0	0	76.8 ± 26.4	35.7
	10	41.8 ± 5.5	23.1	88.4 ± 5.6	26.0
**22**	40	39.2 ± 1.3	5.8	62.0 ± 5.2 ^b^	48.0
	20	41.0 ± 8.6	0	78.2 ± 20.3	0
	10	41.6 ± 5.4	0	92.2 ± 6.5	22.8
**24**	40	30.8 ± 6.7	26.0	12.2 ± 3.3 ^d^	89.7
	20	31.4 ± 5.0	24.5	20.4 ± 10.6 ^d^	82.9
	10	43.2 ± 6.6	0	61.2 ± 6.4 ^b^	48.7
Control		63.8 ± 2.0	-	120.8 ± 3.4	-
**ASA**	200	62.0 ± 8.6	9.2	40.6 ± 14.0 ^c^	66.3
	100	58.5 ± 4.5	14.3	60.3 ± 9.5 ^c^	50.0
	50	59.8 ± 6.3	12.4	108.7 ± 13.8	10.0

^a^ Data are presented as the means ± SEM of 6–8 mice per group. ^b^ *p* < 0.05, ^c^ *p* < 0.01, ^d^ *p* < 0.001 vs. control.

## Data Availability

Not applicable.

## Supplementary Materials

The following supporting information can be downloaded at: https://www.mdpi.com/article/10.3390/ijms241813707/s1.
