# Supplementary material for: Anti-Inflammatory Activities of 8-Benzylaminoxanthines Showing High Adenosine A2A and Dual A1/A2A Receptor Affinity
Source: Int J Mol Sci. 2023 Sep 5;24(18):13707. doi: 10.3390/ijms241813707 (PMC10531311; doi:10.3390/ijms241813707)
Supplement: Supplementary file 1 [file ijms-24-13707-s001.zip › 1H and 13C NMR data.pdf]

Anti-inflammatory activities of 8-benzylaminoxanthines showing potent adenosine A_{2A} receptor antagonistic properties

Michał Załuski[1‡], Dorota Łażewska[1‡*], Piotr Jaśko[2], Ewelina Honkisz-Orzechowska[1], Kamil J. Kuder[1], Andreas Brockmann[2], Gniewomir Latacz[1], Małgorzata Zygmunt[3], Maria Kaleta[1], Beril Anita Greser[1], Agnieszka Olejarz-Maciej[1], Magdalena Jastrzębska-Więsek[4], Christin Vielmuth[2], Christa E. Müller[2], Katarzyna Kieć-Kononowicz[1]

[1] Department of Technology and Biotechnology of Drugs, Faculty of Pharmacy, Jagiellonian University Medical College in Kraków, Medyczna 9, 30-688 Krakow, Poland;

[2] PharmaCenter Bonn, Pharmaceutical Institute, Pharmaceutical & Medicinal Chemistry, University of Bonn, An der Immenburg 4, 53121 Bonn, Germany

[3] Department of Pharmacodynamics, Jagiellonian University Medical College in Kraków, Medyczna 9, 30-688 Kraków, Poland; (Ma.Z.)

[4] Department of Clinical Pharmacy, Jagiellonian University Medical College in Kraków, Medyczna 9, 30-688 Kraków, Poland;

* Correspondence: dorota.lazewska@uj.edu.pl (D.Ł.)

‡ These authors contributed equally to this work.

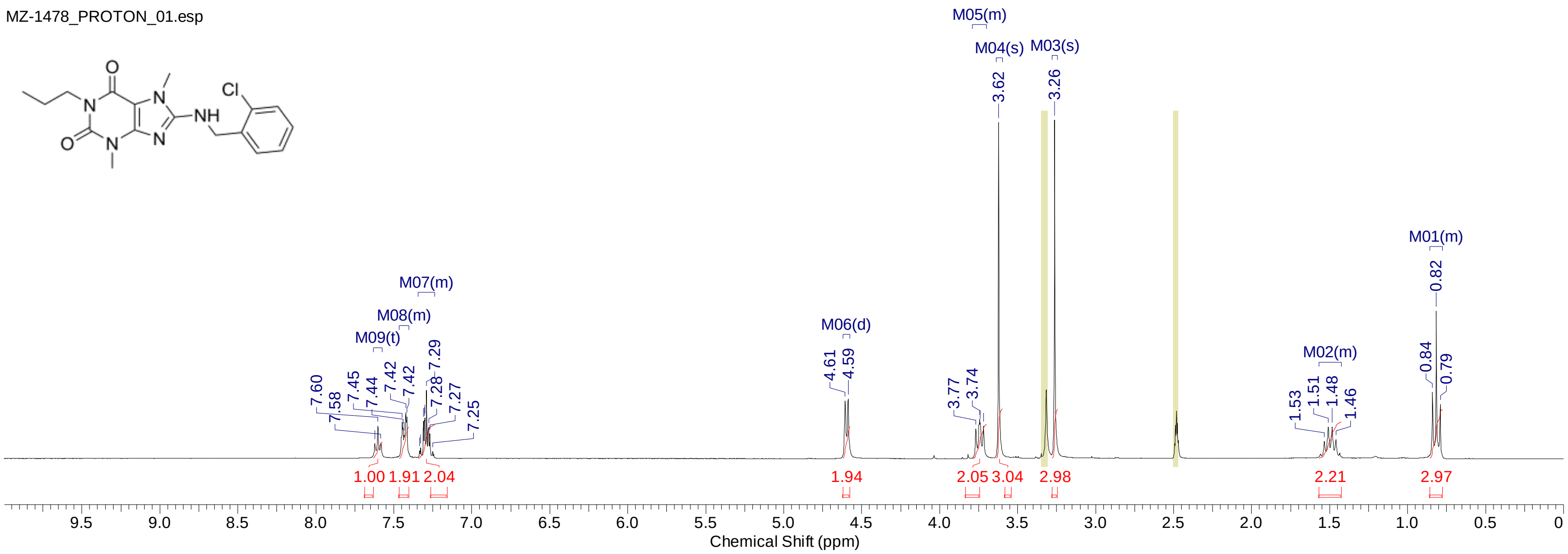

Figure 1S. ^{1}H NMR spectrum of 8-((2-chlorobenzyl)amino)-3,7-dimethyl-1-propyl-3,7-dihydro-1H-purine-2,6-dione (**5**)

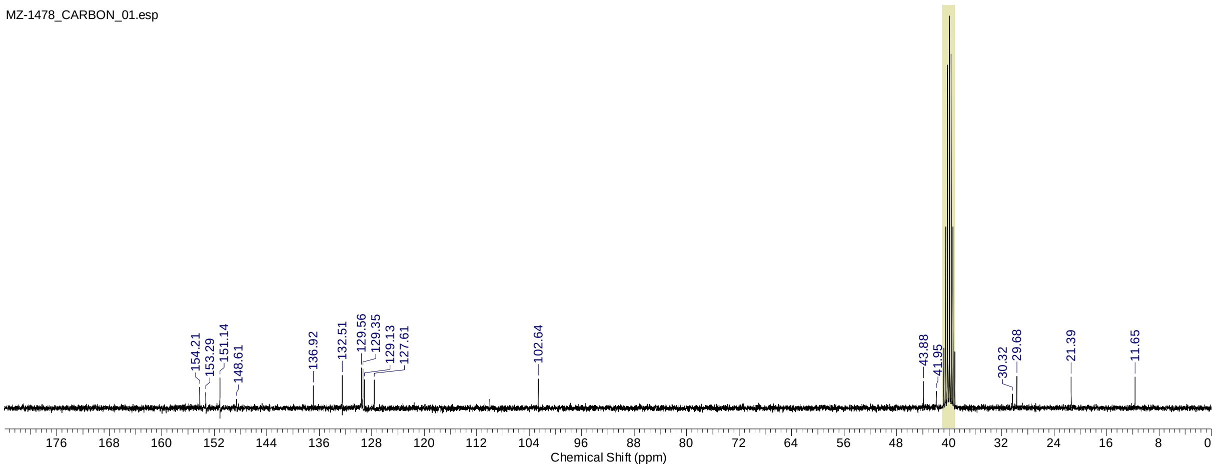

Figure 2S. ^{13}C NMR spectrum of 8-((2-chlorobenzyl)amino)-3,7-dimethyl-1-propyl-3,7-dihydro-1H-purine-2,6-dione (**5**)

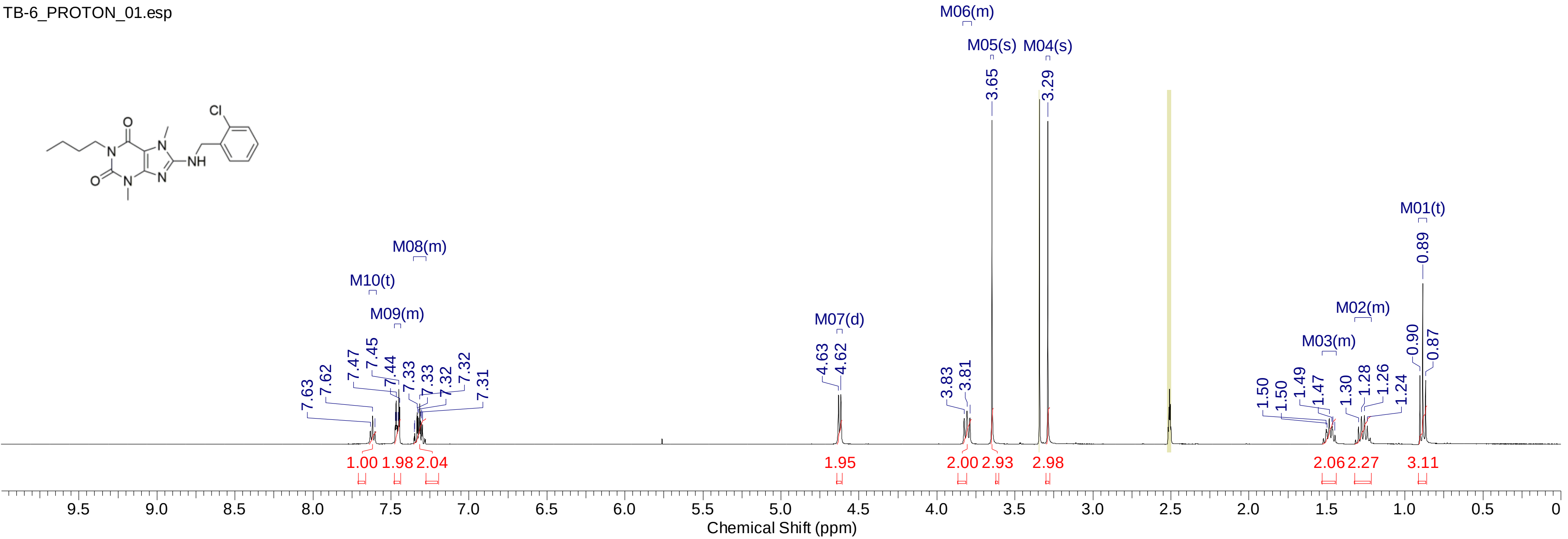

Figure 3S. ^{1}H NMR spectrum of 1-butyl-8-((2-chlorobenzyl)amino)-3,7-dimethyl-3,7-dihydro-1H-purine-2,6-dione (**6**)

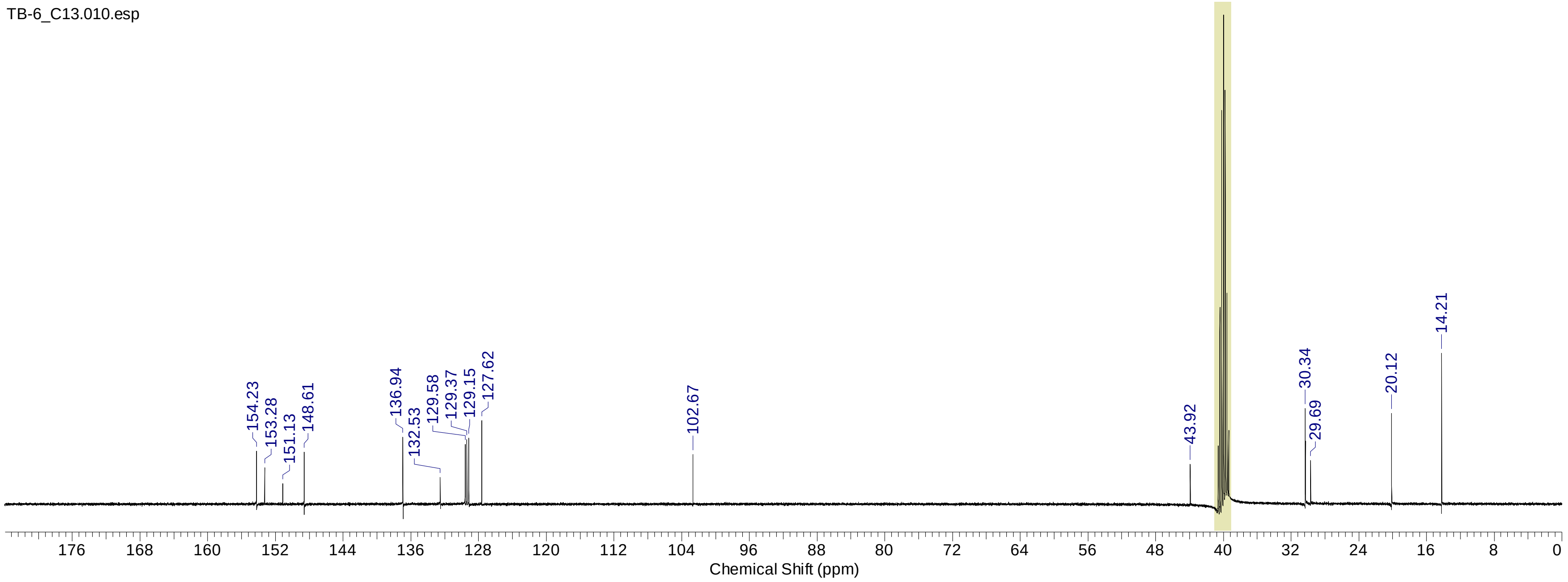

Figure 4S. ^{13}C NMR spectrum of 1-butyl-8-((2-chlorobenzyl)amino)-3,7-dimethyl-3,7-dihydro-1H-purine-2,6-dione (**6**)

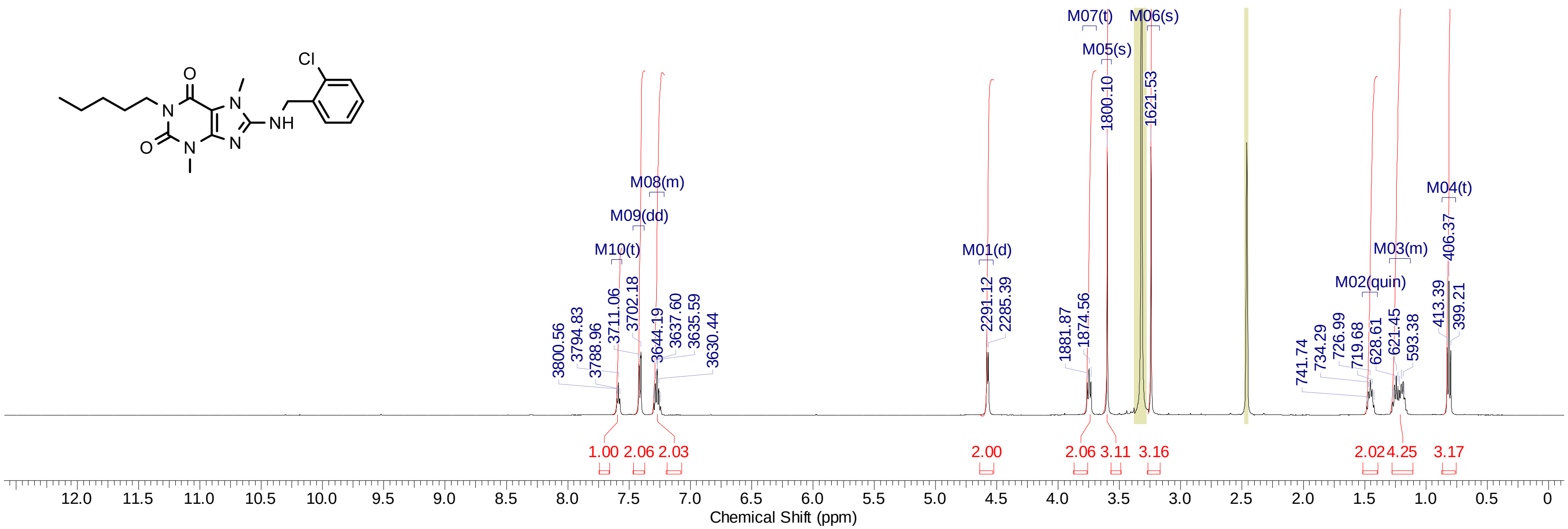

Figure 5S. ^{1}H NMR spectrum of 8-((2-chlorobenzyl)amino)-3,7-dimethyl-1-pentyl-3,7-dihydro-1H-purine-2,6-dione (**7**)

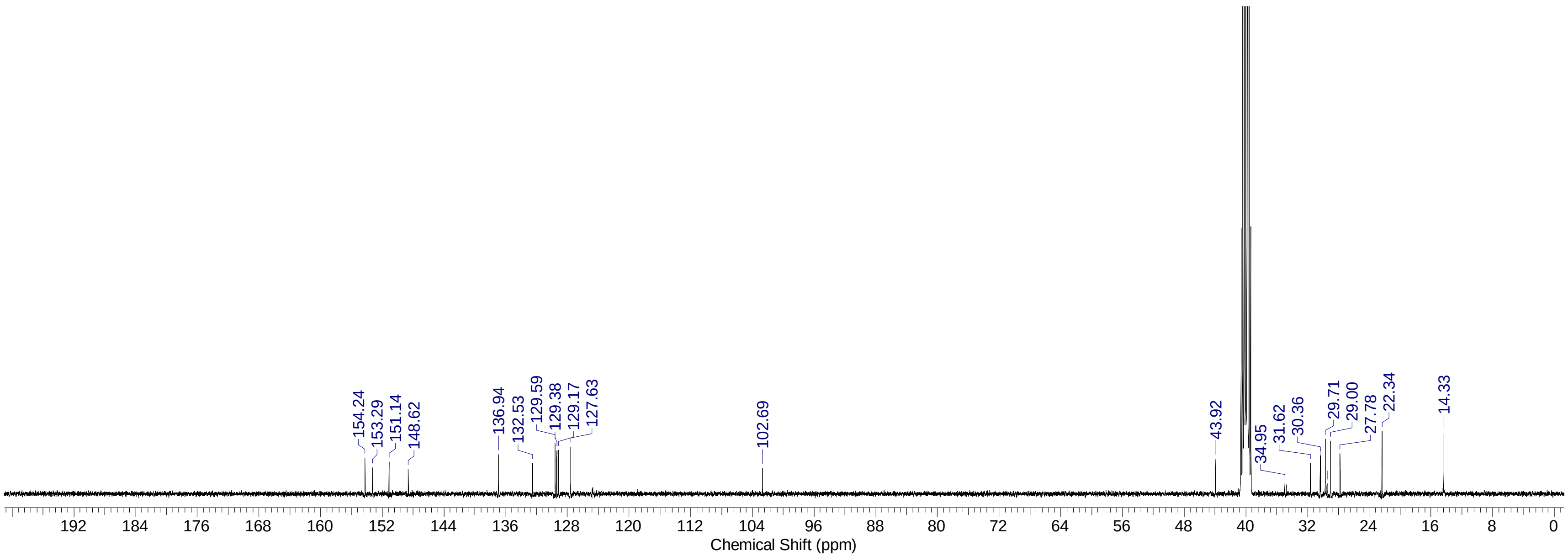

Figure 6S. ^{13}C NMR spectrum of 8-((2-chlorobenzyl)amino)-3,7-dimethyl-1-pentyl-3,7-dihydro-1H-purine-2,6-dione (**7**)

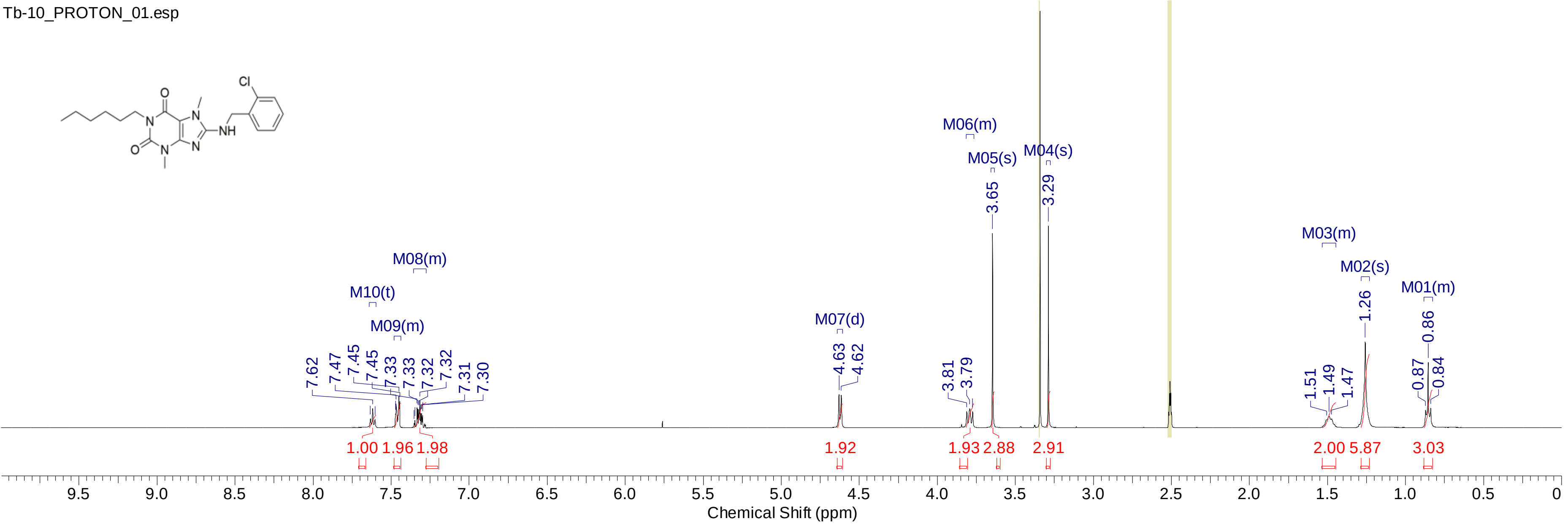

Figure 7S. ^{1}H NMR spectrum of 8-((2-chlorobenzyl)amino)-1-hexyl-3,7-dimethyl-3,7-dihydro-1H-purine-2,6-dione (**8**)

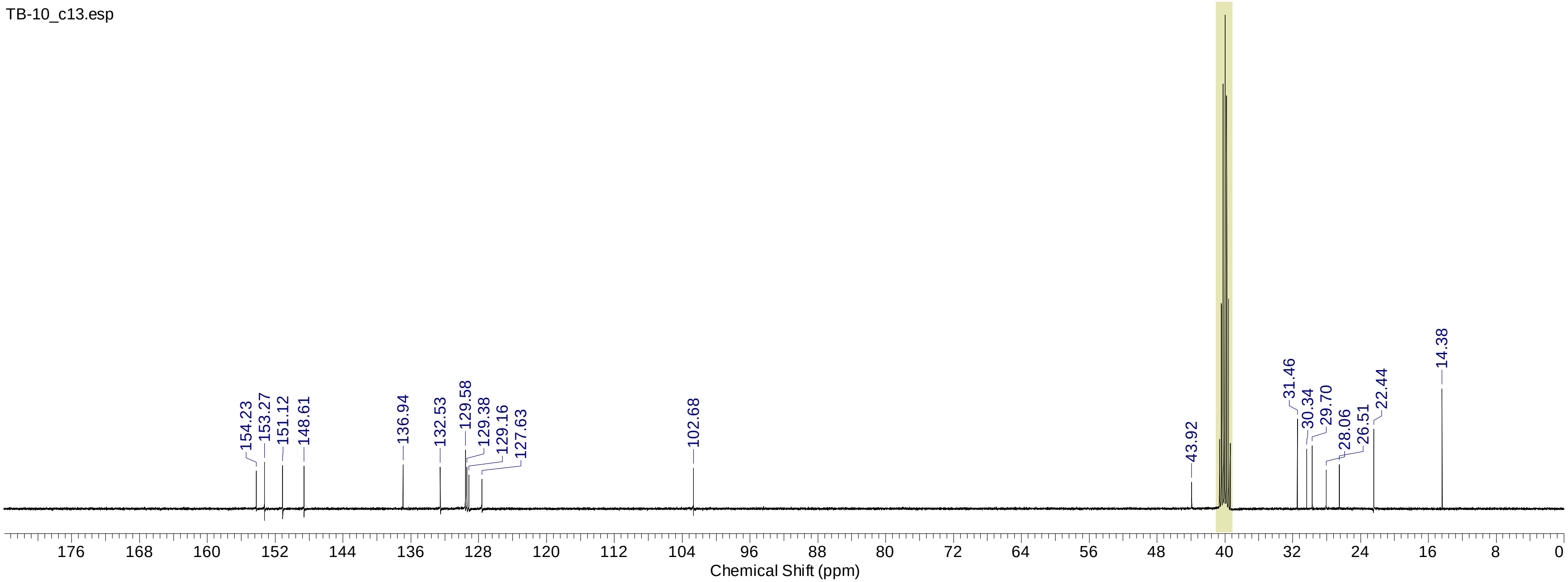

Figure 8S. ^{13}C NMR spectrum of 8-((2-chlorobenzyl)amino)-1-hexyl-3,7-dimethyl-3,7-dihydro-1H-purine-2,6-dione (**8**)

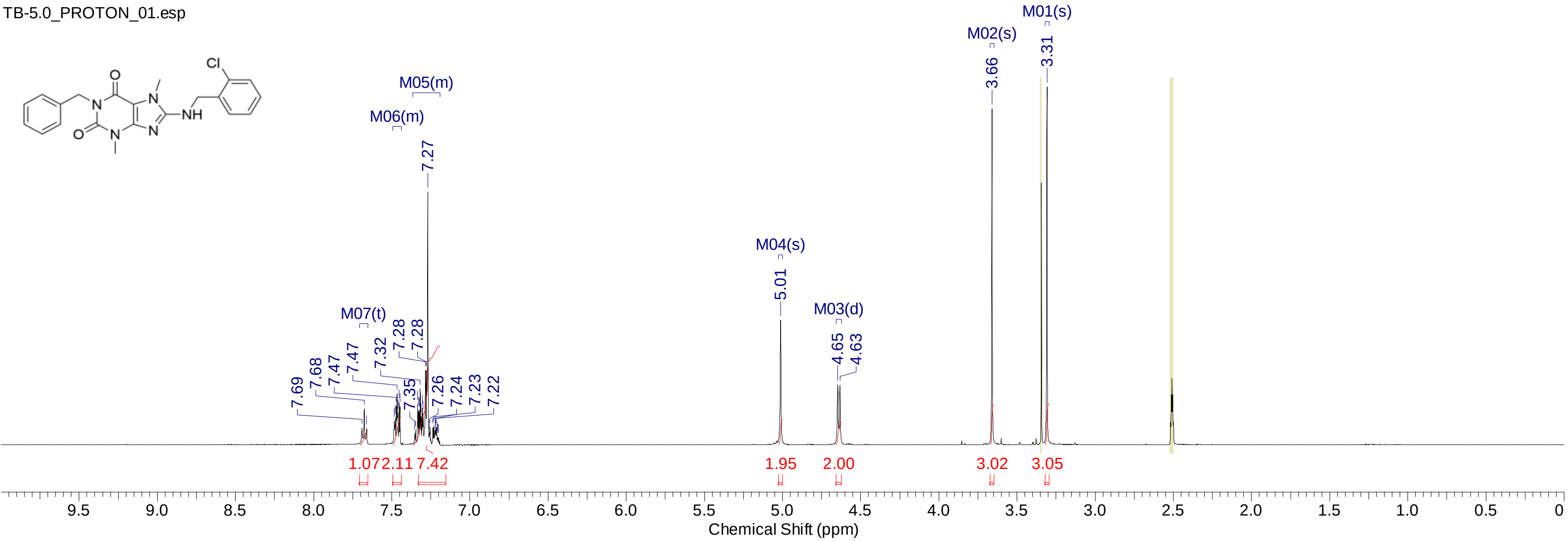

Figure 9S. ^{1}H NMR spectrum of 1-benzyl-8-((2-chlorobenzyl)amino)-3,7-dimethyl-1-propyl-3,7-dihydro-1H-purine-2,6-dione (**9**)

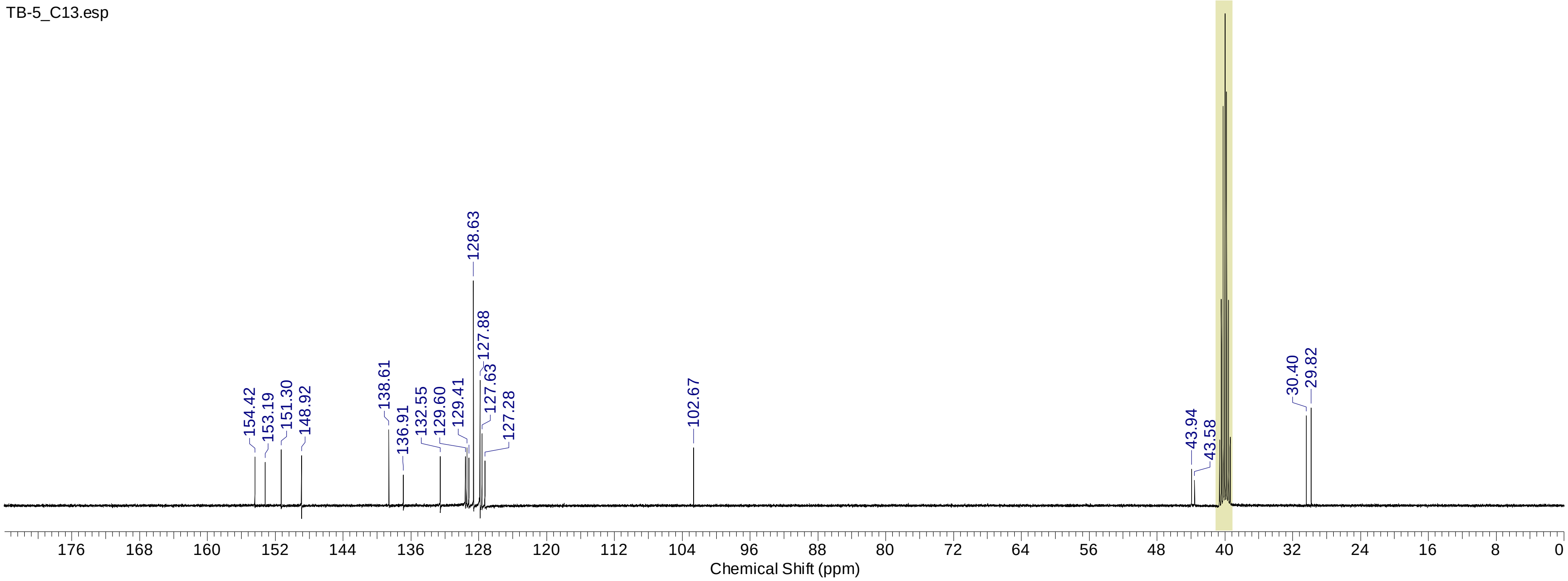

Figure 10S. ^{13}C NMR spectrum of 1-benzyl-8-((2-chlorobenzyl)amino)-3,7-dimethyl-1-propyl-3,7-dihydro-1H-purine-2,6-dione (**9**)

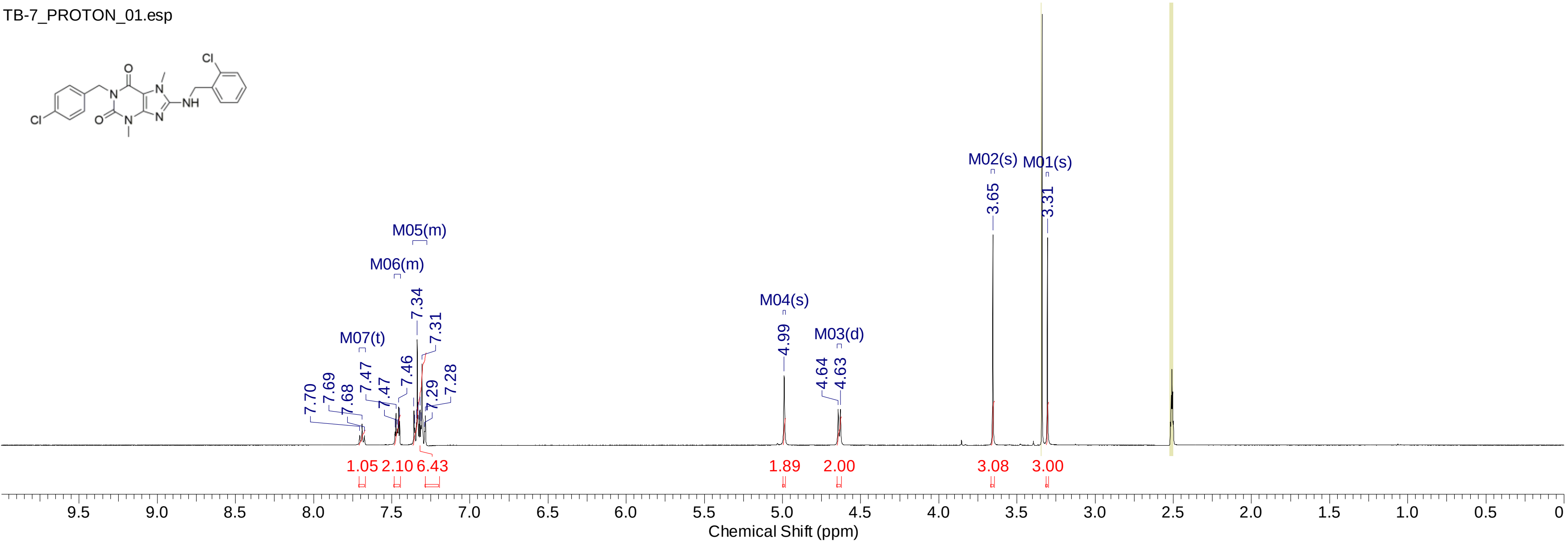

Figure 11S. ^{1}H NMR spectrum of 1-(4-chlorobenzyl)-8-((2-chlorobenzyl)amino)-3,7-dimethyl-3,7-dihydro-1H-purine-2,6-dione (**10**)

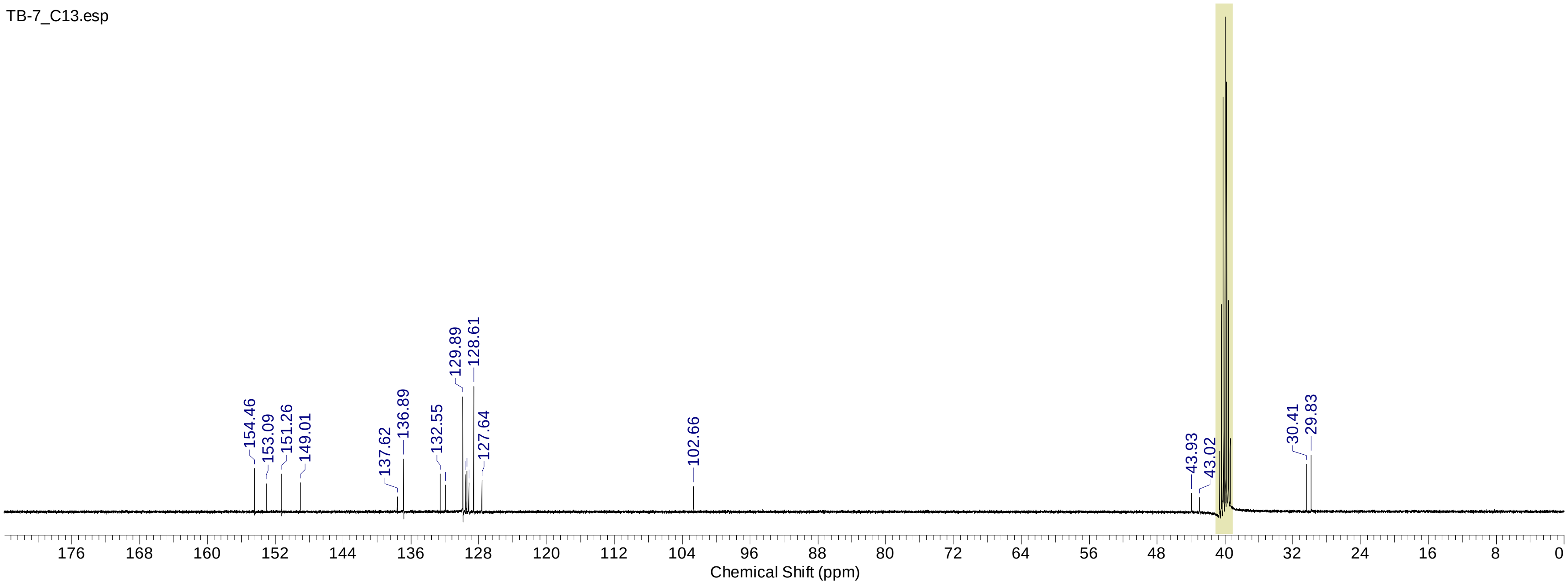

Figure 12S. ^{13}C NMR spectrum of 1-(4-chlorobenzyl)-8-((2-chlorobenzyl)amino)-3,7-dimethyl-3,7-dihydro-1H-purine-2,6-dione (**10**)

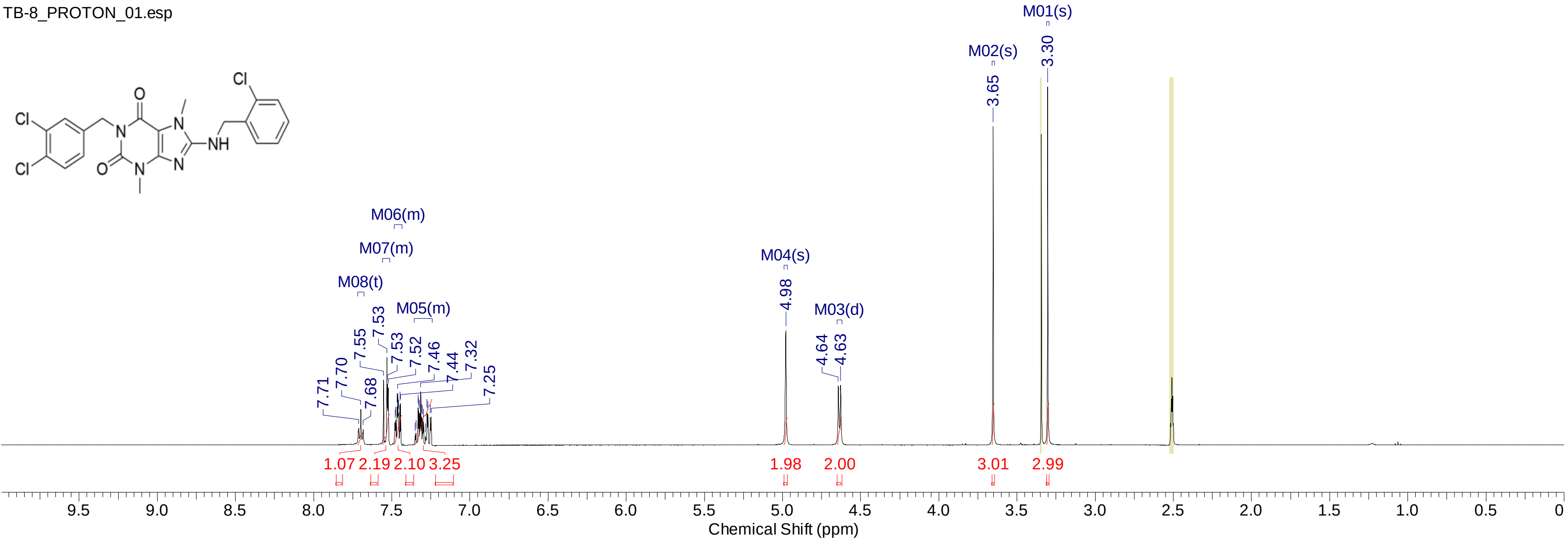

Figure 13S. ^{1}H NMR spectrum of 8-((2-chlorobenzyl)amino)-1-(3,4-dichlorobenzyl)-3,7-dimethyl-3,7-dihydro-1H-purine-2,6-dione (**11**)

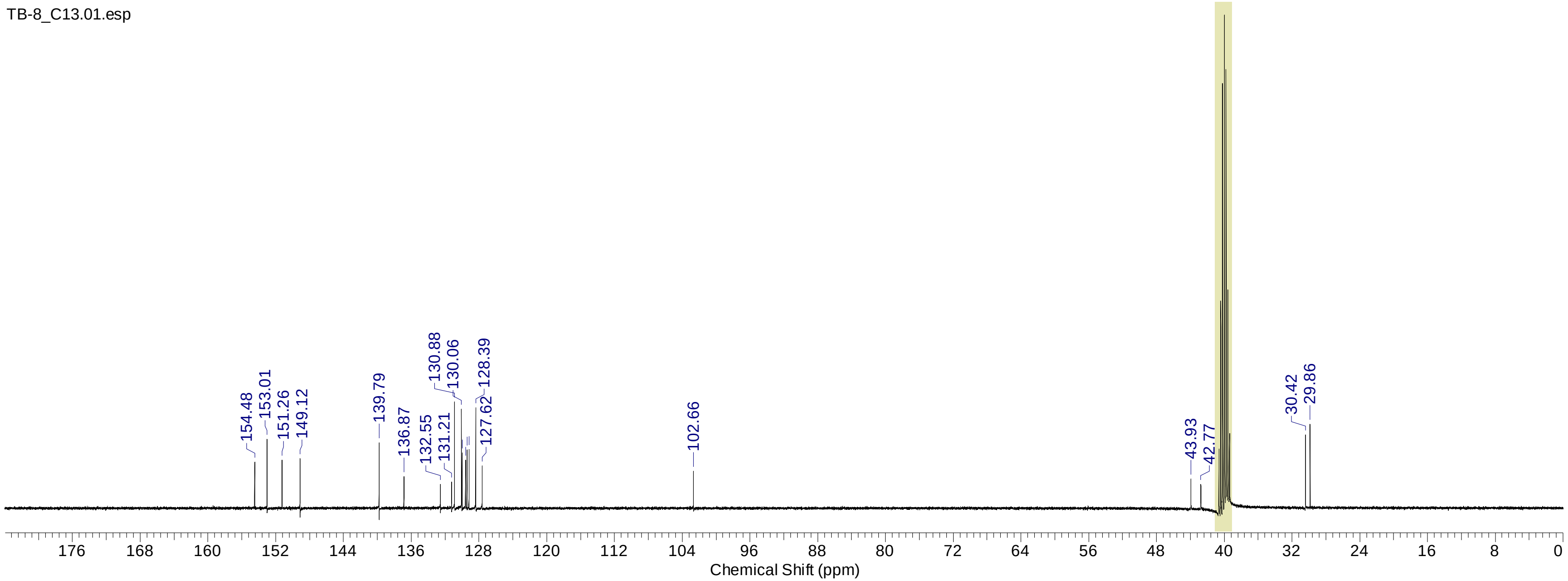

Figure 14S. ^{13}C NMR spectrum of 8-((2-chlorobenzyl)amino)-1-(3,4-dichlorobenzyl)-3,7-dimethyl-3,7-dihydro-1H-purine-2,6-dione (**11**)

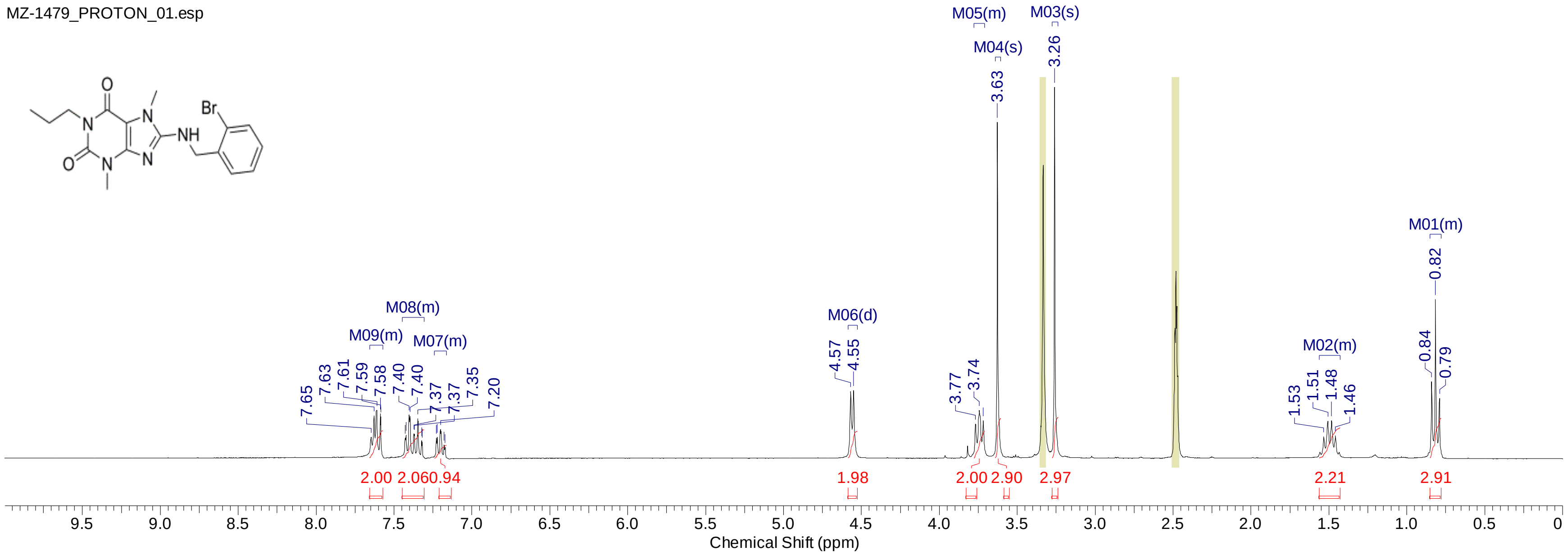

Figure 15S. ^{1}H NMR spectrum of 8-((2-bromobenzyl)amino)-3,7-dimethyl-1-propyl-3,7-dihydro-1H-purine-2,6-dione (**12**)

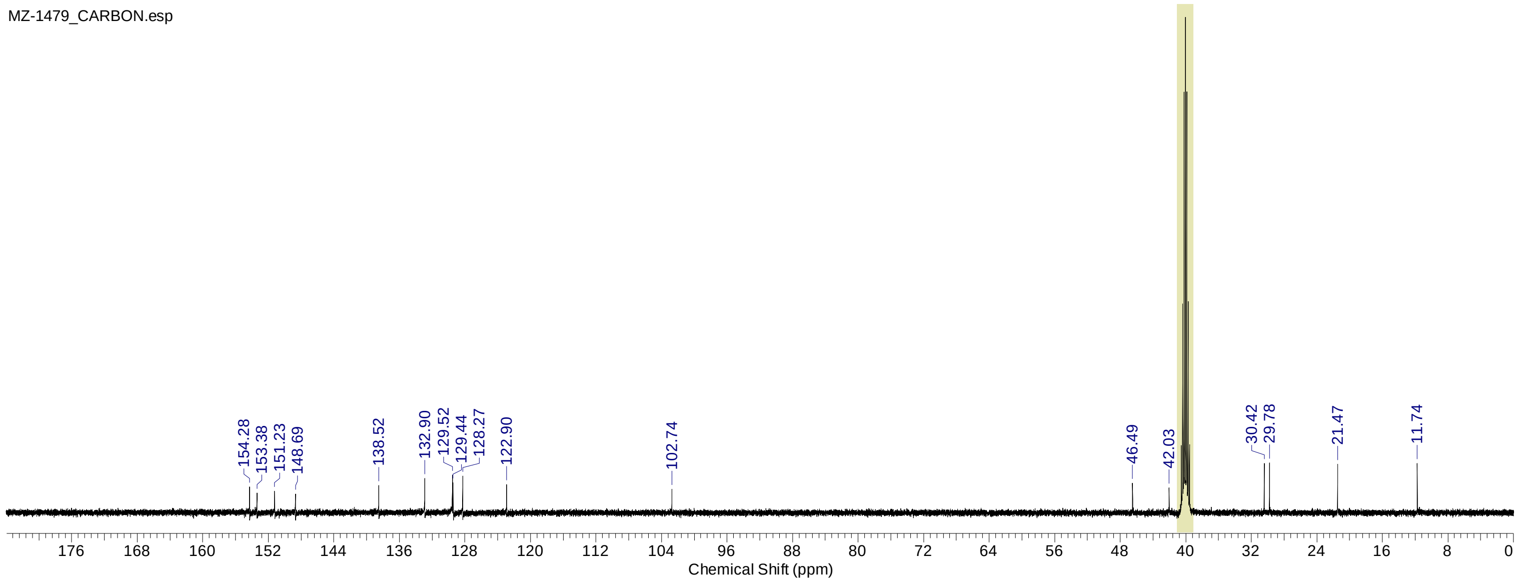

Figure 16S. ^{13}C NMR spectrum of 8-((2-bromobenzyl)amino)-3,7-dimethyl-1-propyl-3,7-dihydro-1H-purine-2,6-dione (**12**)

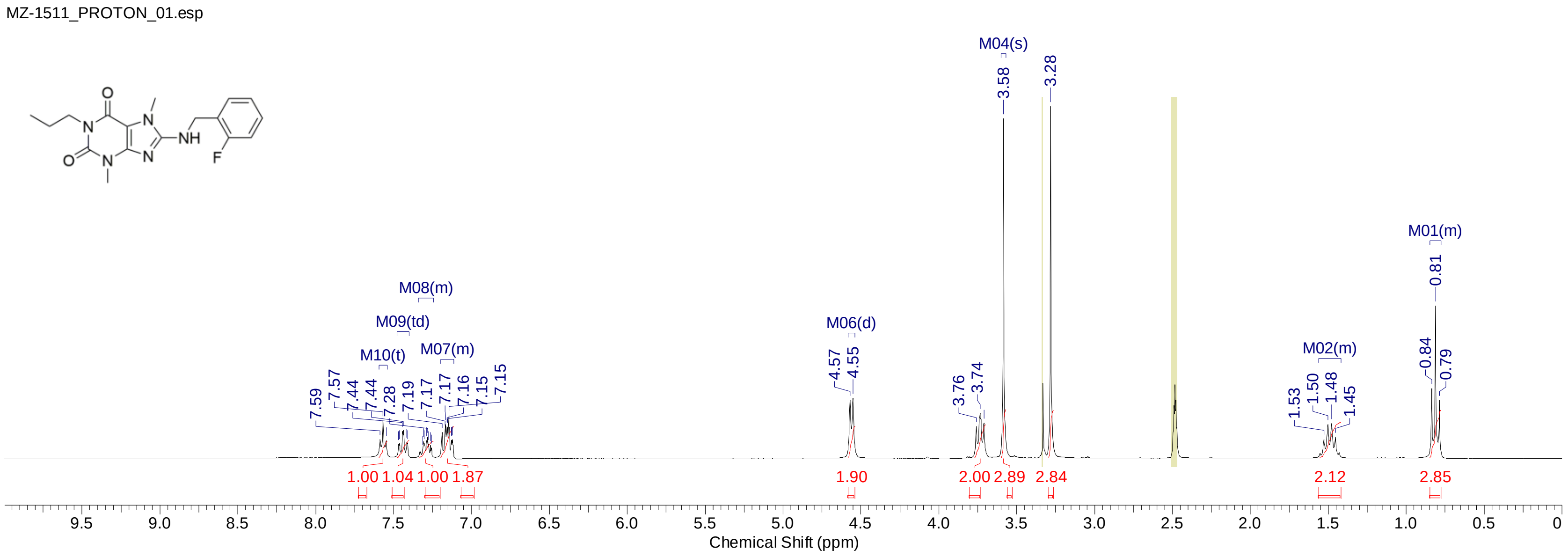

Figure 17S. ^{1}H NMR spectrum of 8-((2-fluorobenzyl)amino)-3,7-dimethyl-1-propyl-3,7-dihydro-1H-purine-2,6-dione (**13**)

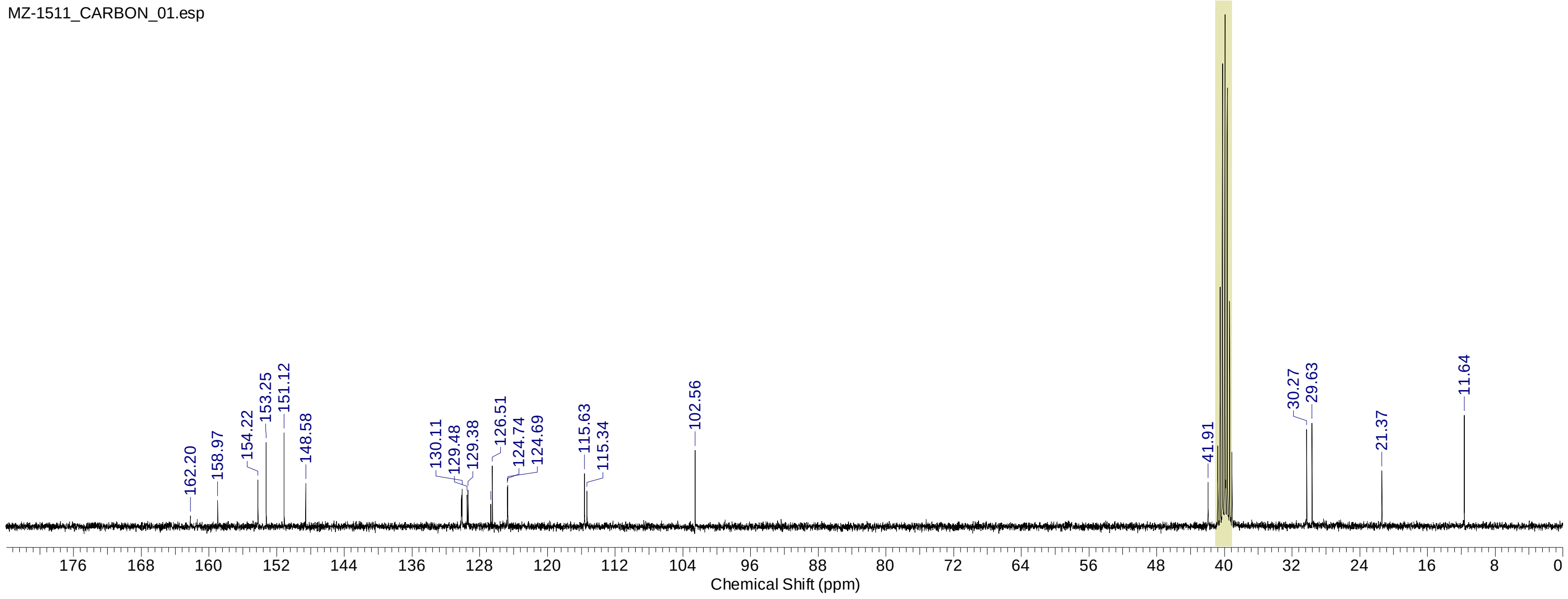

Figure 18S. ^{13}C NMR spectrum of 8-((2-fluorobenzyl)amino)-3,7-dimethyl-1-propyl-3,7-dihydro-1H-purine-2,6-dione (**13**)

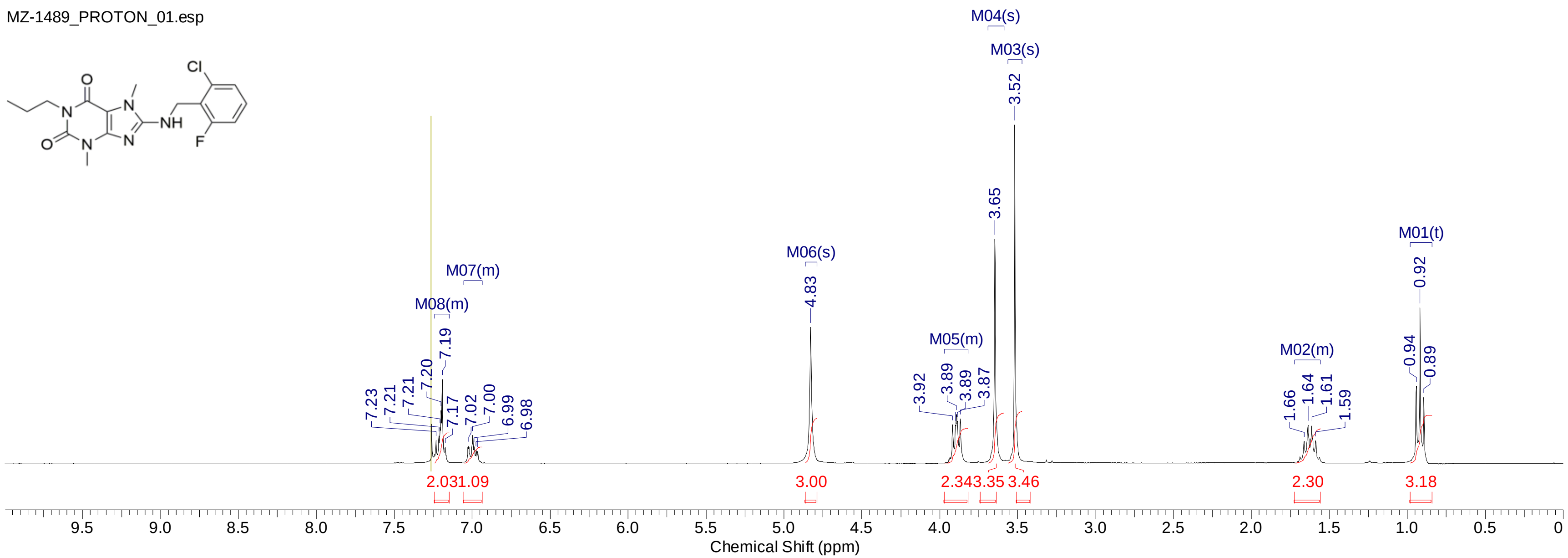

Figure 19S. ^{1}H NMR spectrum of 8-((2-chloro-6-fluorobenzyl)amino)-3,7-dimethyl-1-propyl-3,7-dihydro-1H-purine-2,6-dione (**14**)

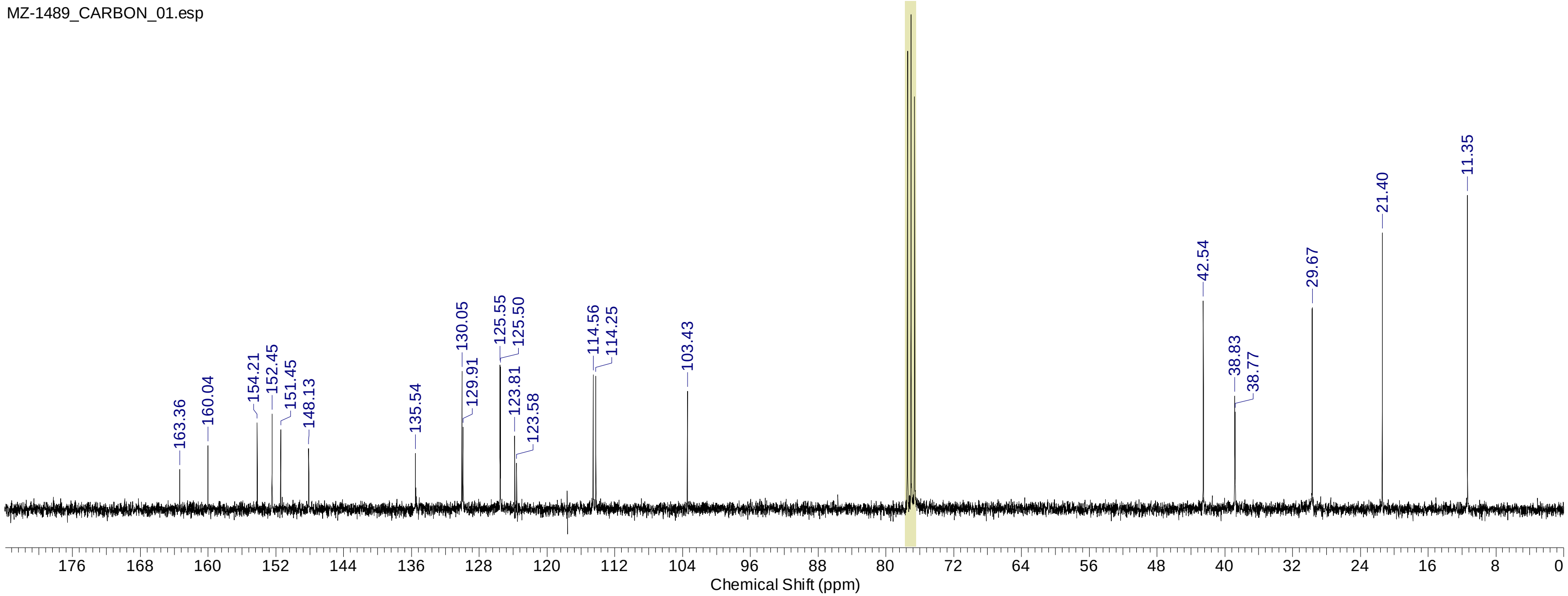

Figure 20S. ^{13}C NMR spectrum of 8-((2-chloro-6-fluorobenzyl)amino)-3,7-dimethyl-1-propyl-3,7-dihydro-1H-purine-2,6-dione (**14**)

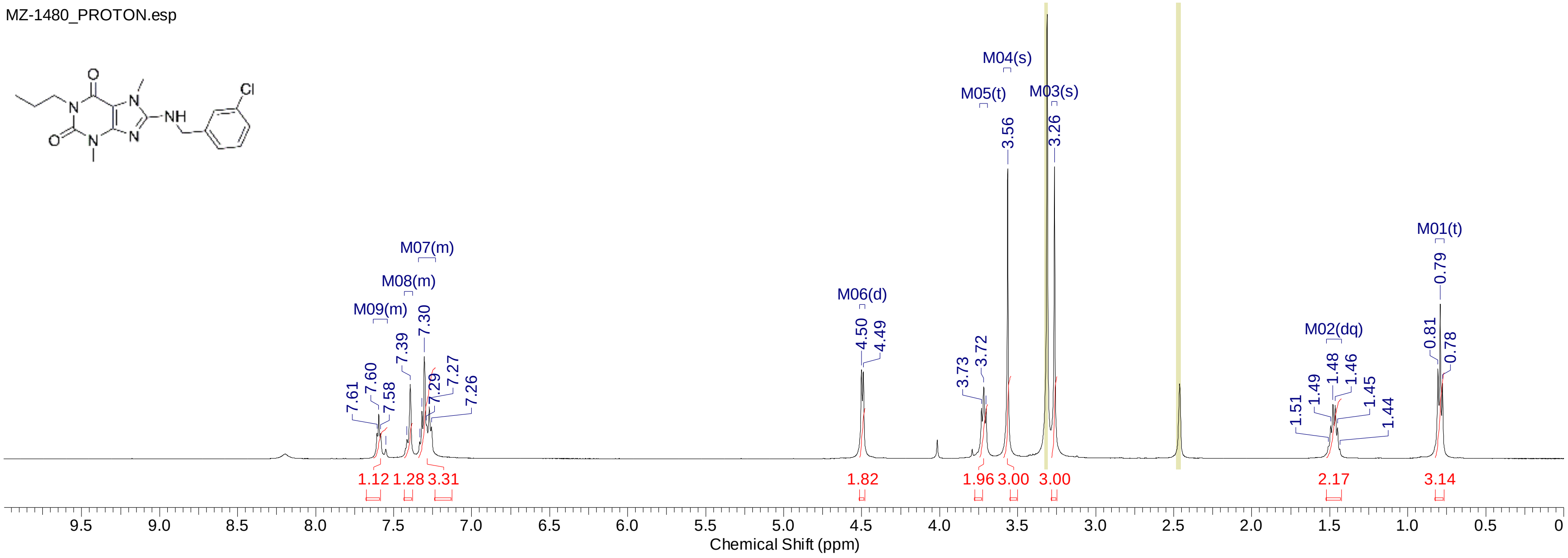

Figure 21S. ^{1}H NMR spectrum of 8-((3-chlorobenzyl)amino)-3,7-dimethyl-1-propyl-3,7-dihydro-1H-purine-2,6-dione (**15**)

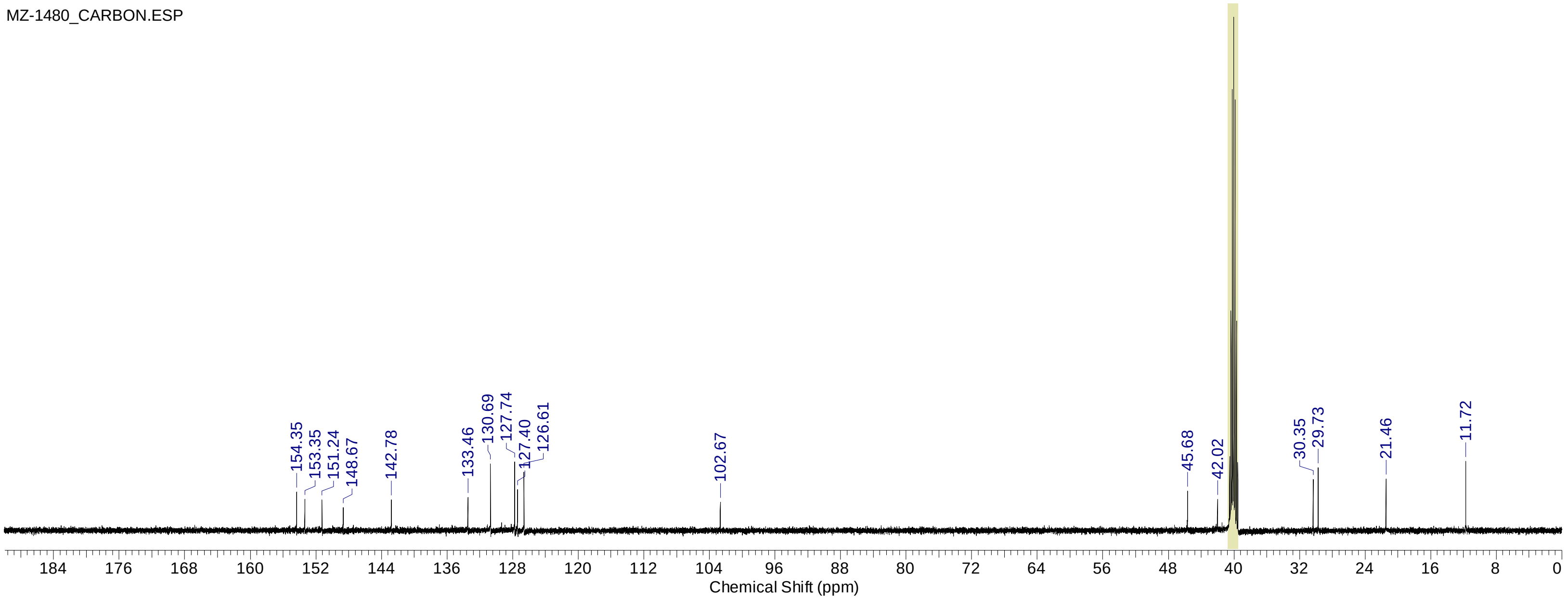

Figure 22S. ^{13}C NMR spectrum of 8-((3-chlorobenzyl)amino)-3,7-dimethyl-1-propyl-3,7-dihydro-1H-purine-2,6-dione (**15**)

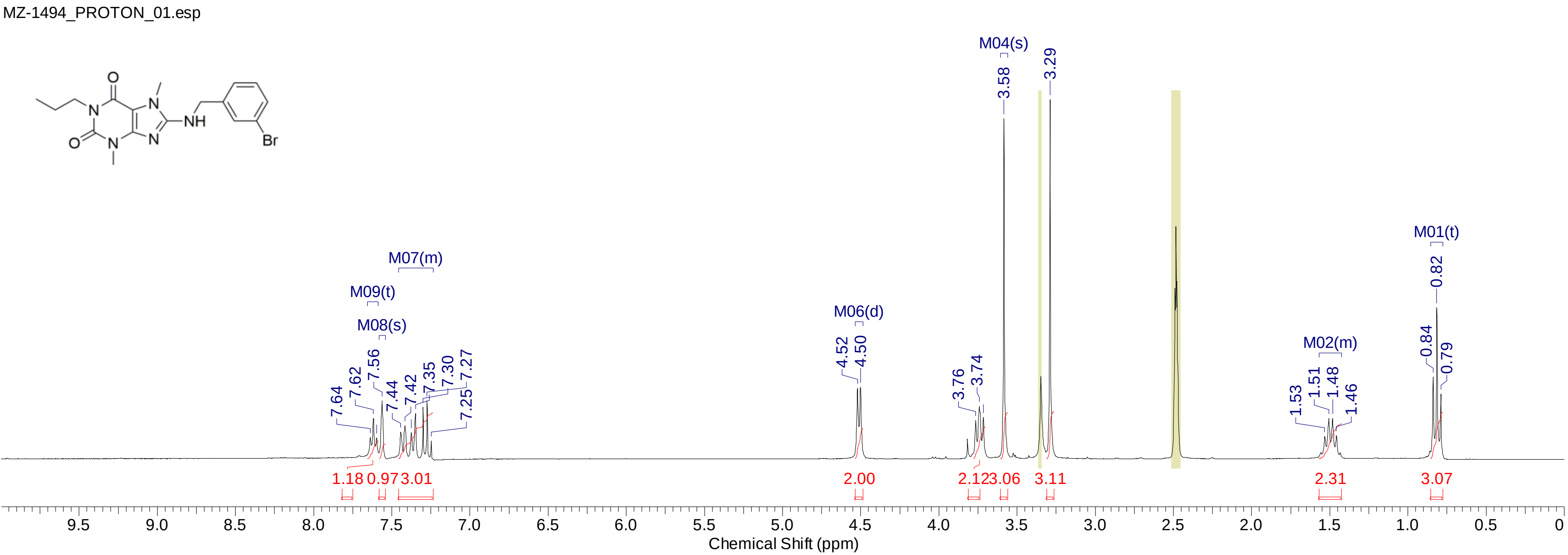

Figure 23S. ^{1}H NMR spectrum of 8-((3-bromobenzyl)amino)-3,7-dimethyl-1-propyl-3,7-dihydro-1H-purine-2,6-dione (**16**)

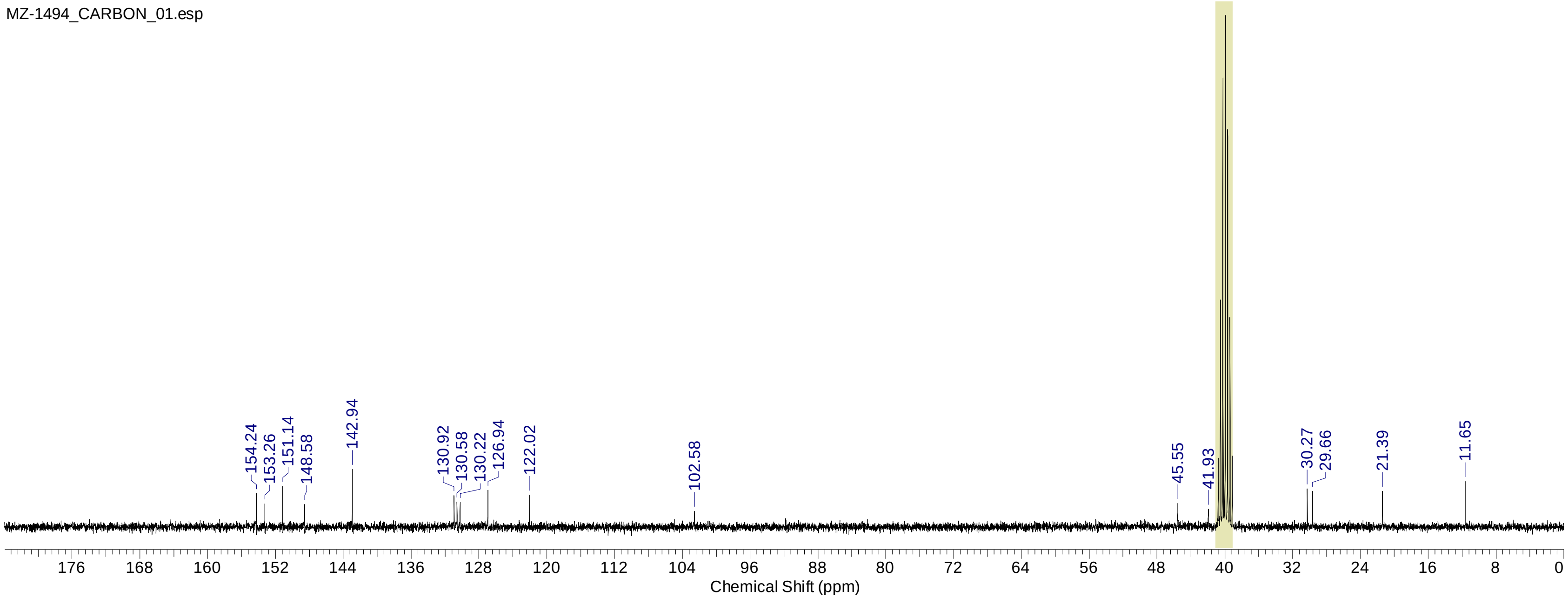

Figure 24S. ^{1}H NMR spectrum of 8-((3-bromobenzyl)amino)-3,7-dimethyl-1-propyl-3,7-dihydro-1H-purine-2,6-dione (**16**)

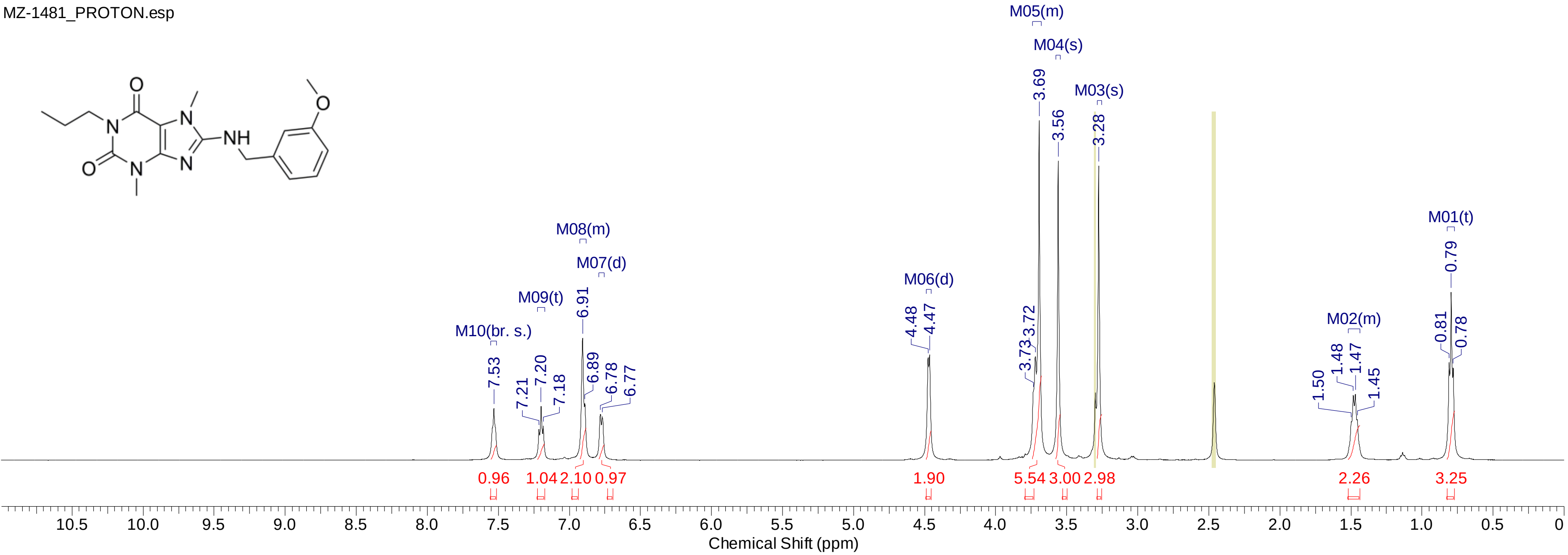

Figure 25S. ^{1}H NMR spectrum of 8-((3-methoxybenzyl)amino)-3,7-dimethyl-1-propyl-3,7-dihydro-1H-purine-2,6-dione (**17**)

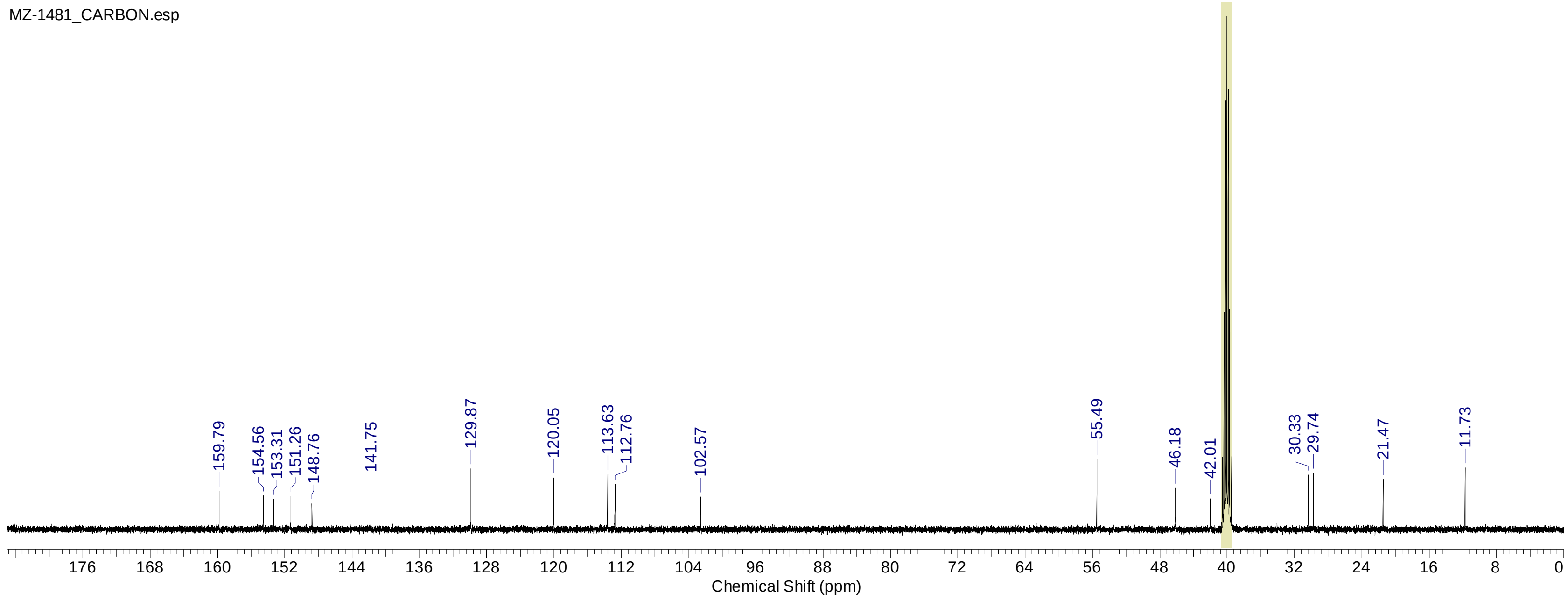

Figure 26S. ^{1}H NMR spectrum of 8-((3-methoxybenzyl)amino)-3,7-dimethyl-1-propyl-3,7-dihydro-1H-purine-2,6-dione (**17**)

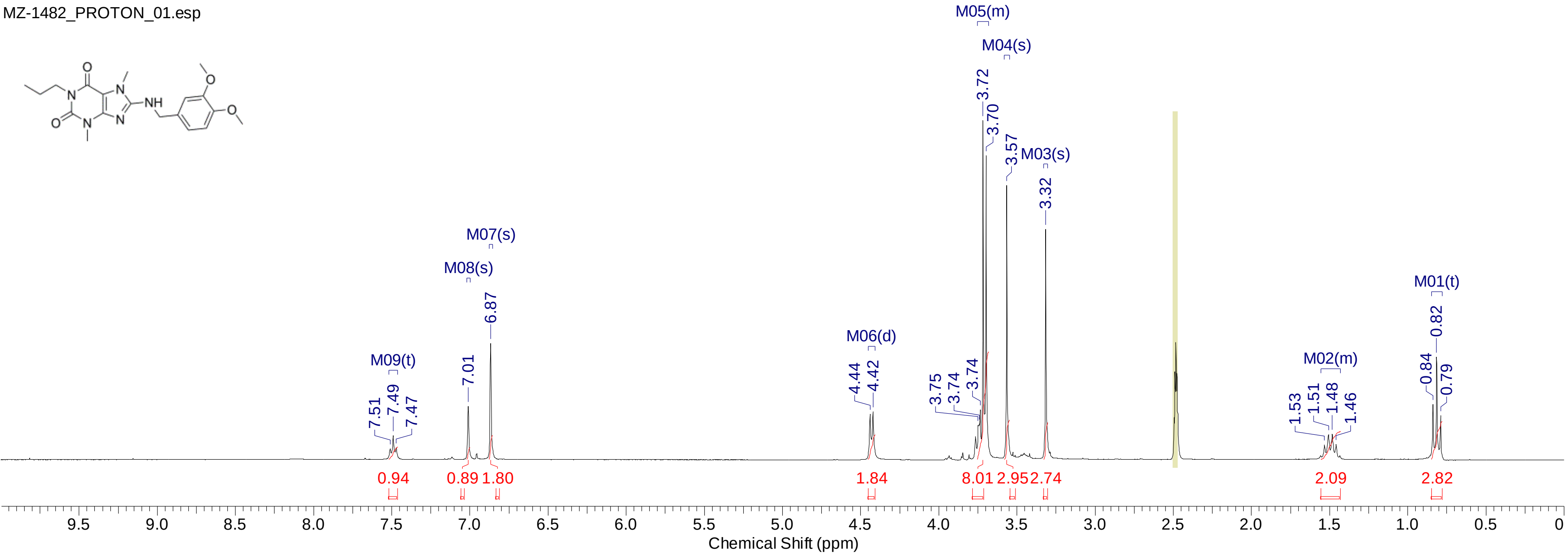

Figure 27S. ^{1}H NMR spectrum of 8-((3,4-dimethoxybenzyl)amino)-3,7-dimethyl-1-propyl-3,7-dihydro-1H-purine-2,6-dione (**18**)

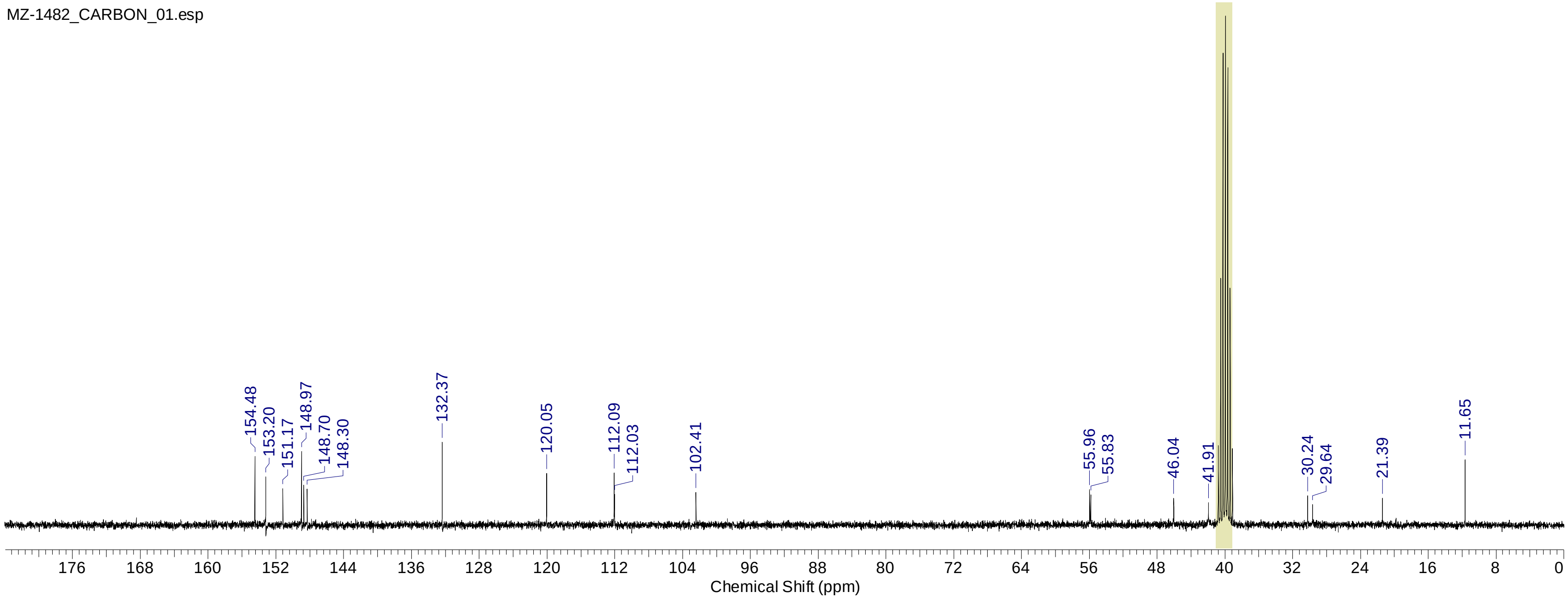

Figure 28S. ^{1}H NMR spectrum of 8-((3,4-dimethoxybenzyl)amino)-3,7-dimethyl-1-propyl-3,7-dihydro-1H-purine-2,6-dione (**18**)

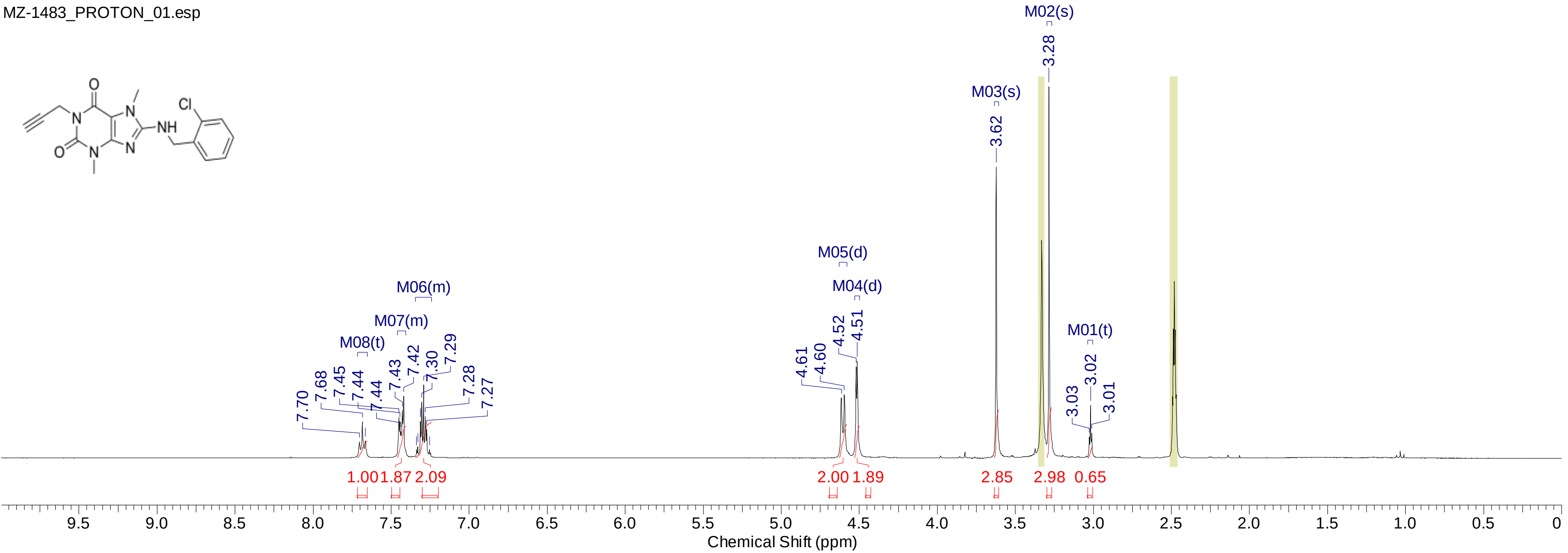

Figure 29S. ^{1}H NMR spectrum of 8-((2-chlorobenzyl)amino)-3,7-dimethyl-1-(prop-2-yn-1-yl)-3,7-dihydro-1H-purine-2,6-dione (**19**)

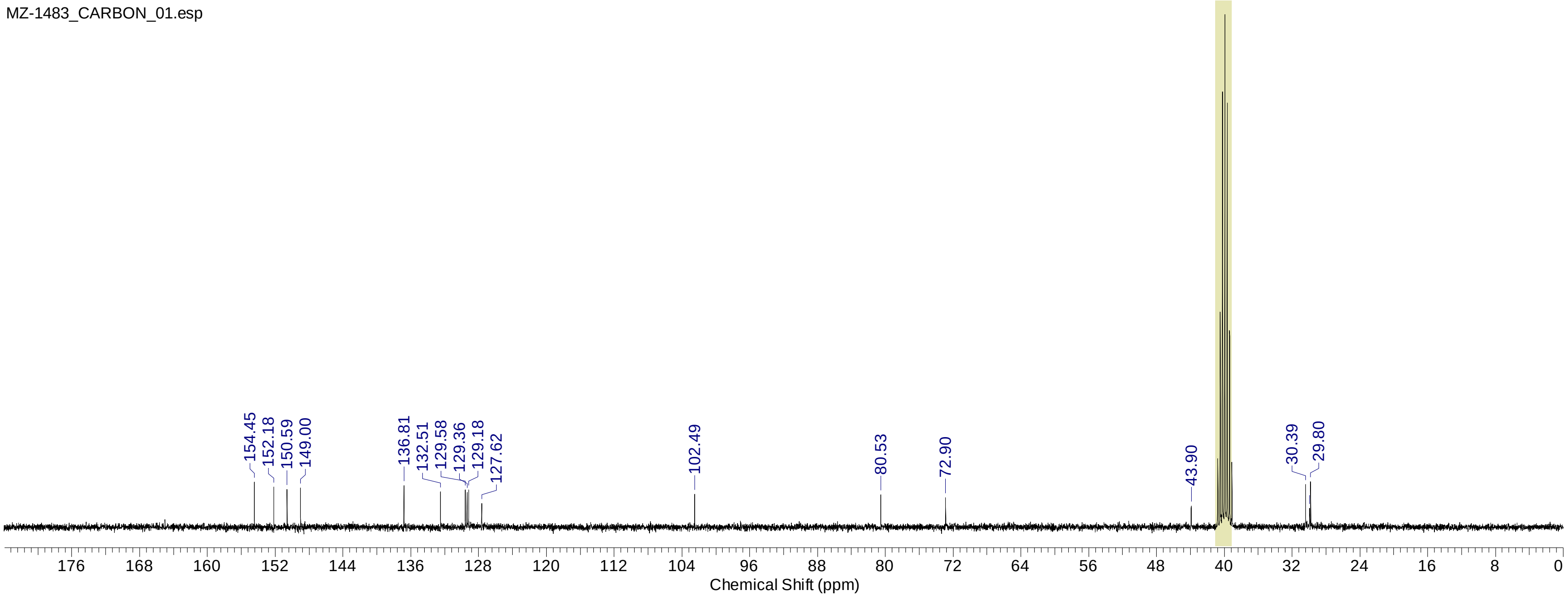

Figure 30S. ^{13}C NMR spectrum of 8-((2-chlorobenzyl)amino)-3,7-dimethyl-1-(prop-2-yn-1-yl)-3,7-dihydro-1H-purine-2,6-dione (**19**)

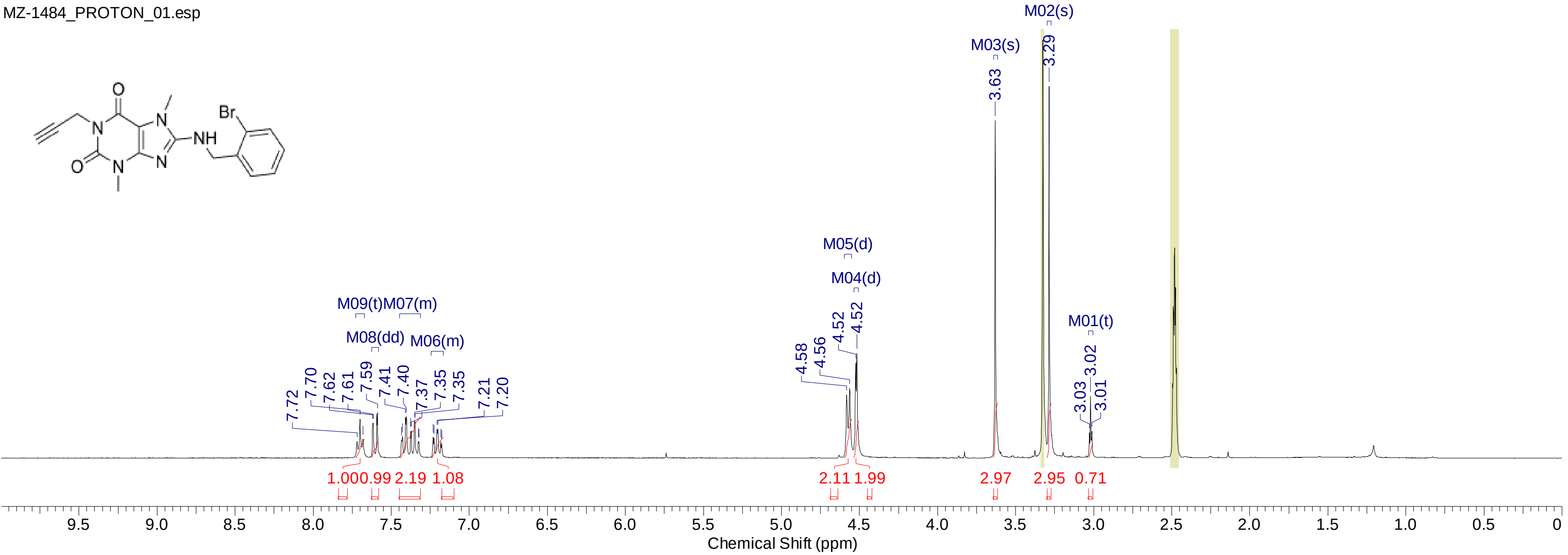

Figure 31S. ^{1}H NMR spectrum of 8-((2-bromobenzyl)amino)-3,7-dimethyl-1-(prop-2-yn-1-yl)-3,7-dihydro-1H-purine-2,6-dione (**20**)

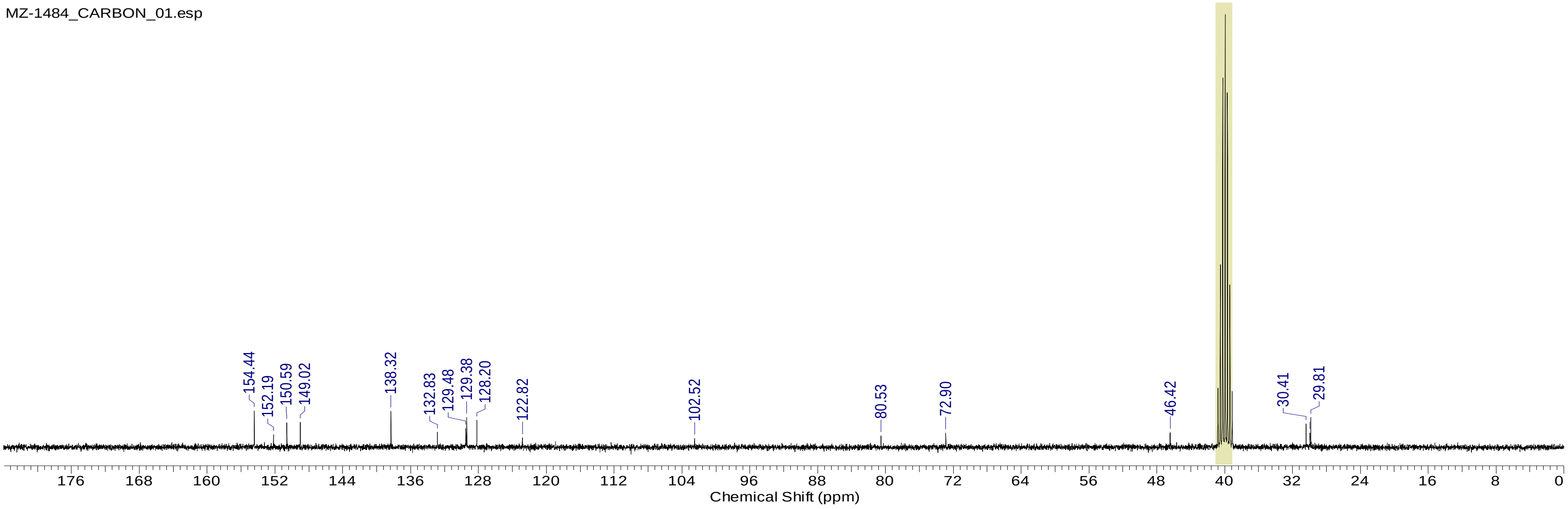

Figure 32S. ^{13}C NMR spectrum of 8-((2-bromobenzyl)amino)-3,7-dimethyl-1-(prop-2-yn-1-yl)-3,7-dihydro-1H-purine-2,6-dione (**20**)

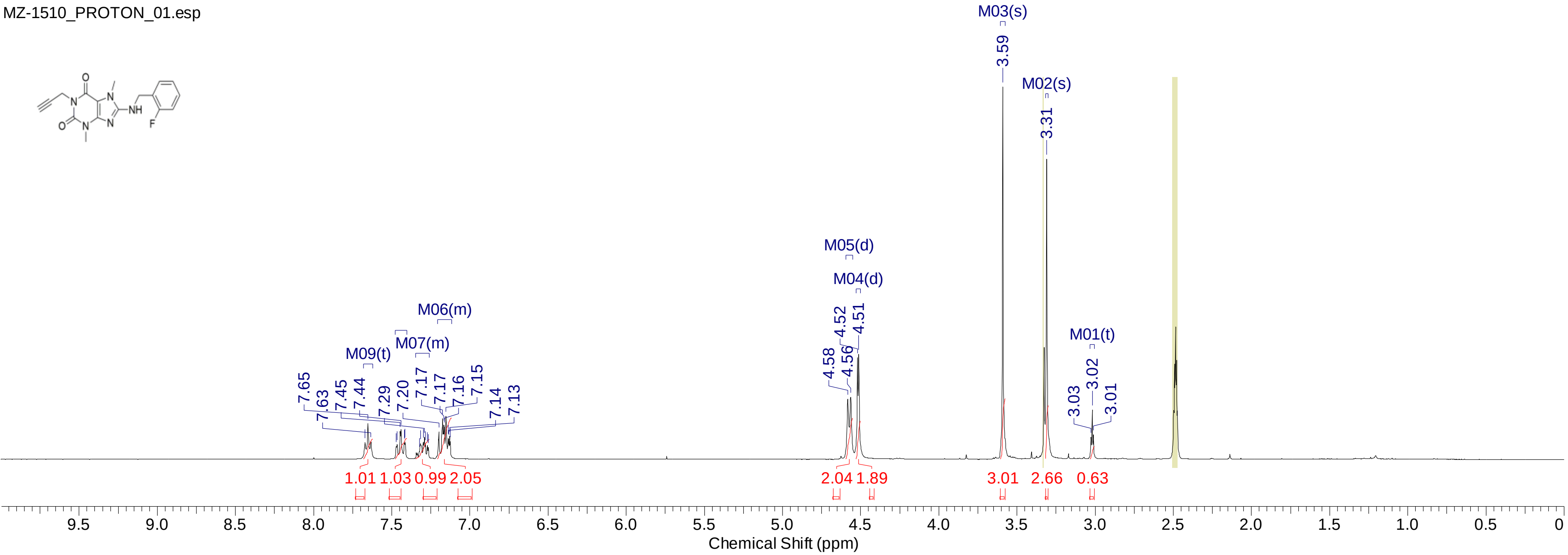

Figure 33S. ^{1}H NMR spectrum of 8-((2-fluorobenzyl)amino)-3,7-dimethyl-1-(prop-2-yn-1-yl)-3,7-dihydro-1H-purine-2,6-dione (**21**)

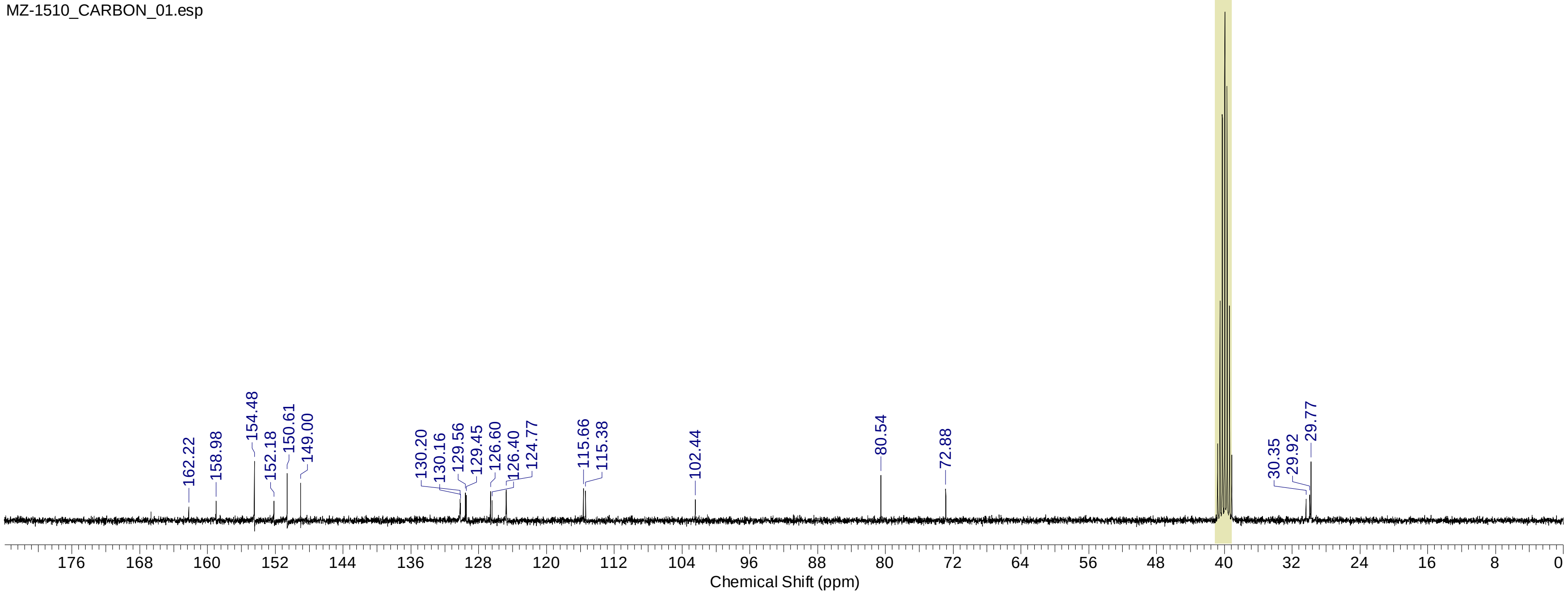

Figure 34S. ^{13}C NMR spectrum of 8-((2-fluorobenzyl)amino)-3,7-dimethyl-1-(prop-2-yn-1-yl)-3,7-dihydro-1H-purine-2,6-dione (**21**)

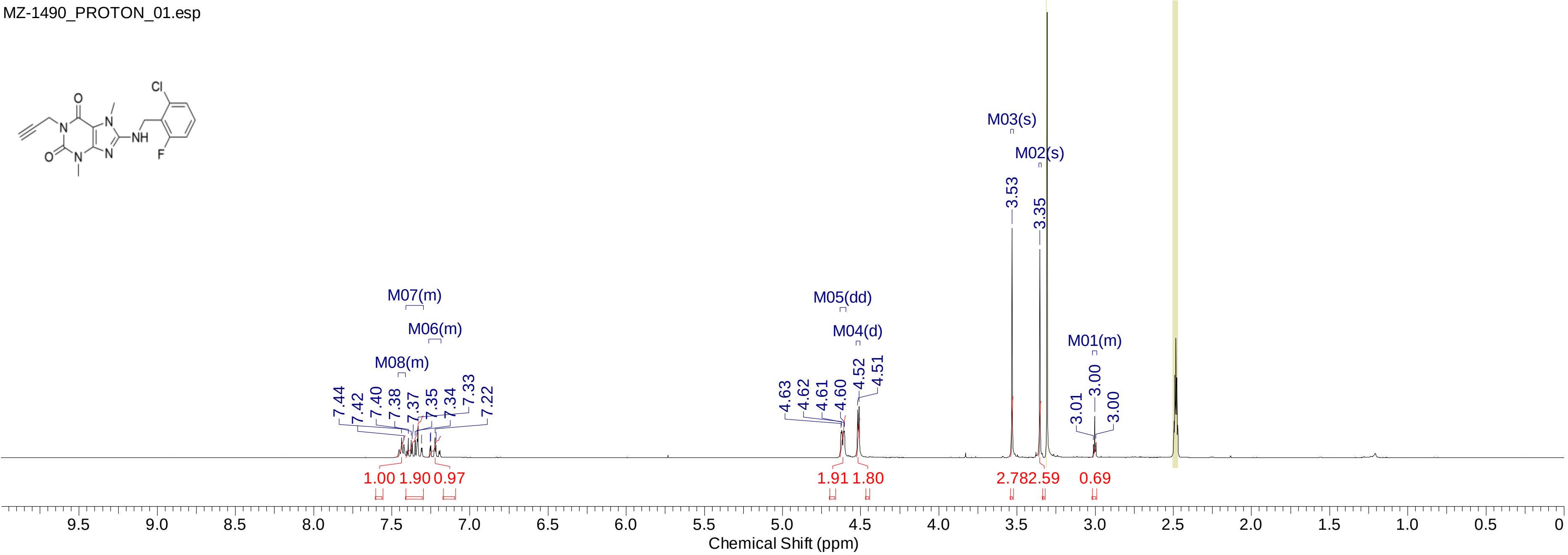

Figure 35S. ^{1}H NMR spectrum of 8-((2-chloro-6-fluorobenzyl)amino)-3,7-dimethyl-1-(prop-2-yn-1-yl)-3,7-dihydro-1H-purine-2,6-dione (**22**)

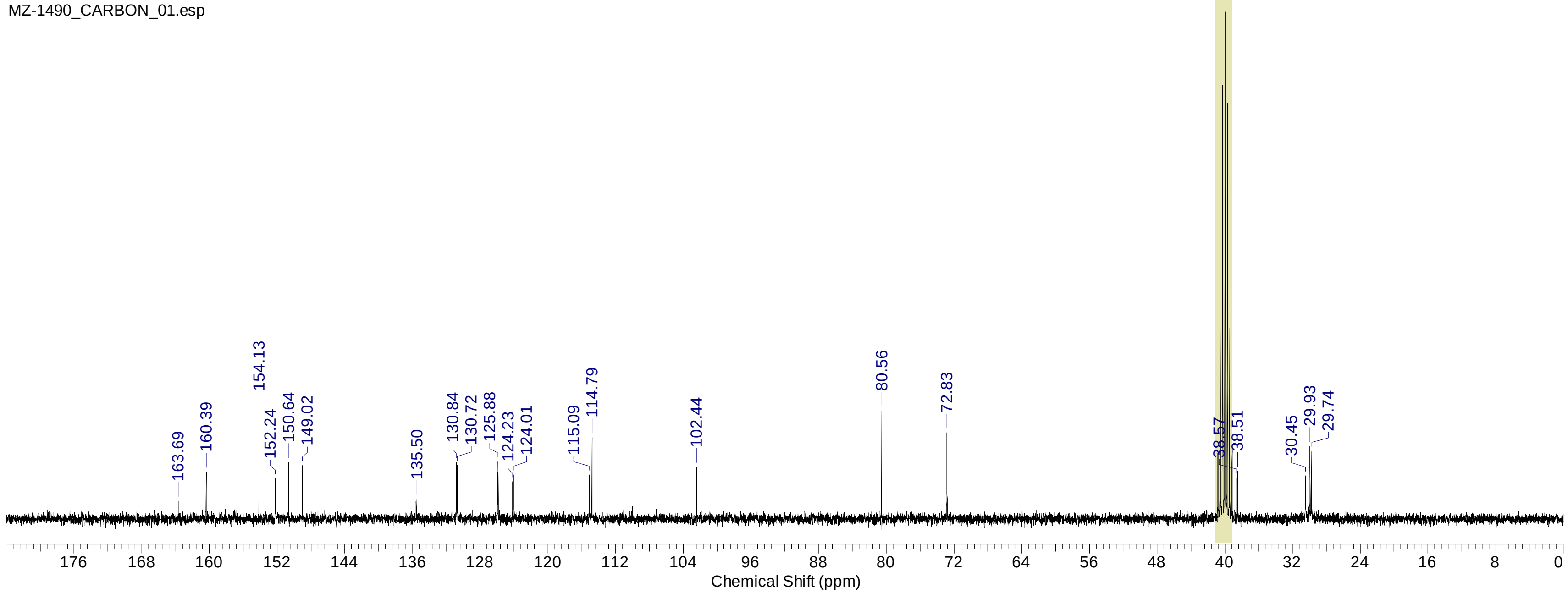

Figure 36S. ^{13}C NMR spectrum of of 8-((2-chloro-6-fluorobenzyl)amino)-3,7-dimethyl-1-(prop-2-yn-1-yl)-3,7-dihydro-1H-purine-2,6-dione (**22**)

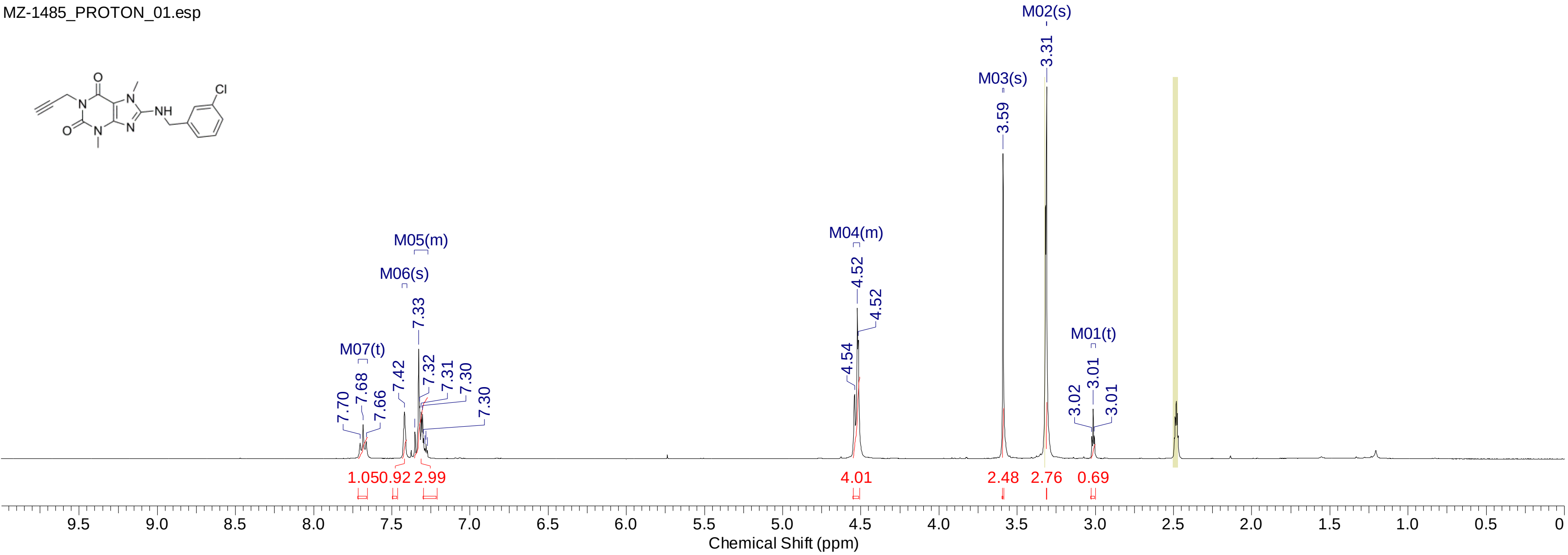

Figure 37S. ^{1}H NMR spectrum of 8-((3-chlorobenzyl)amino)-3,7-dimethyl-1-(prop-2-yn-1-yl)-3,7-dihydro-1H-purine-2,6-dione (**23**)

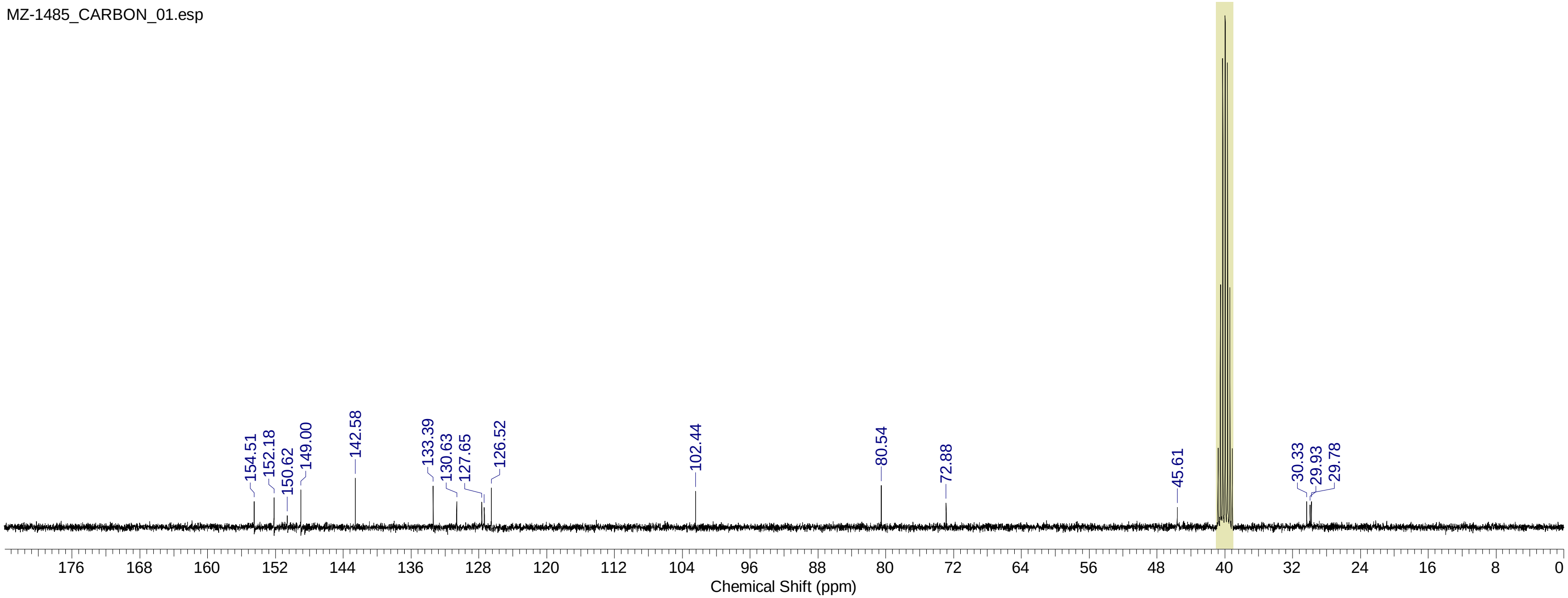

Figure 38S. ^{13}C NMR spectrum of 8-((3-chlorobenzyl)amino)-3,7-dimethyl-1-(prop-2-yn-1-yl)-3,7-dihydro-1H-purine-2,6-dione (**23**)

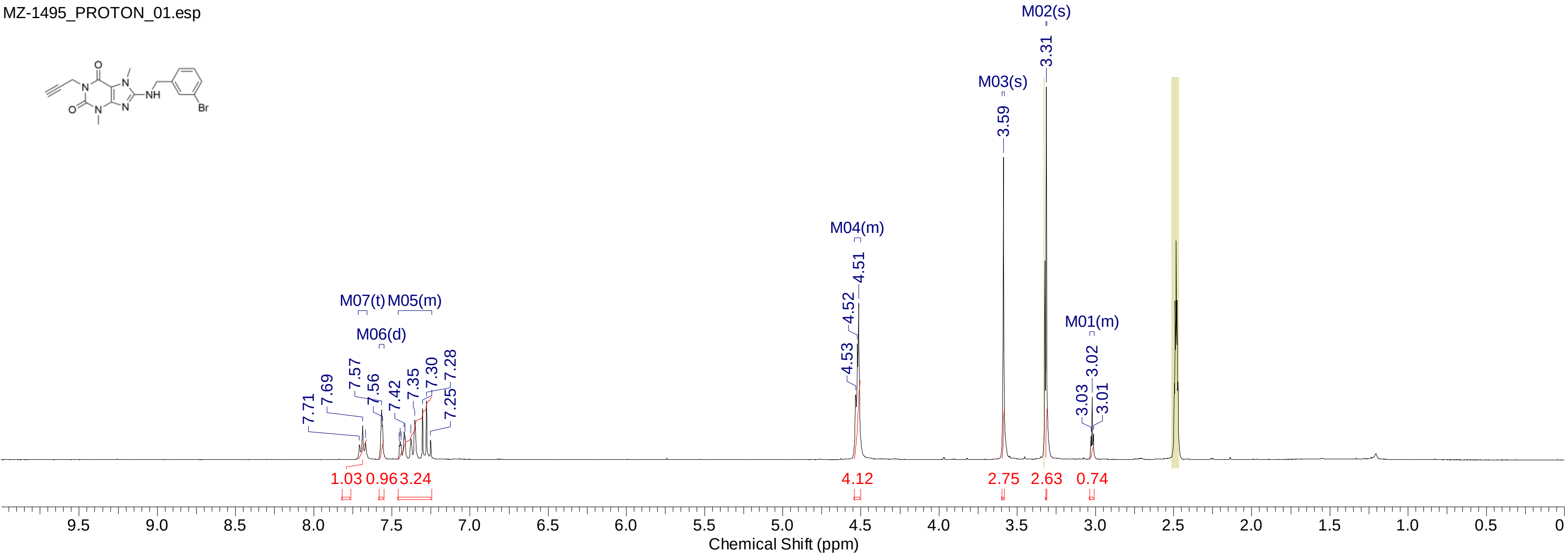

Figure 39S. ^{1}H NMR spectrum of 8-((3-bromobenzyl)amino)-3,7-dimethyl-1-(prop-2-yn-1-yl)-3,7-dihydro-1H-purine-2,6-dione (**24**)

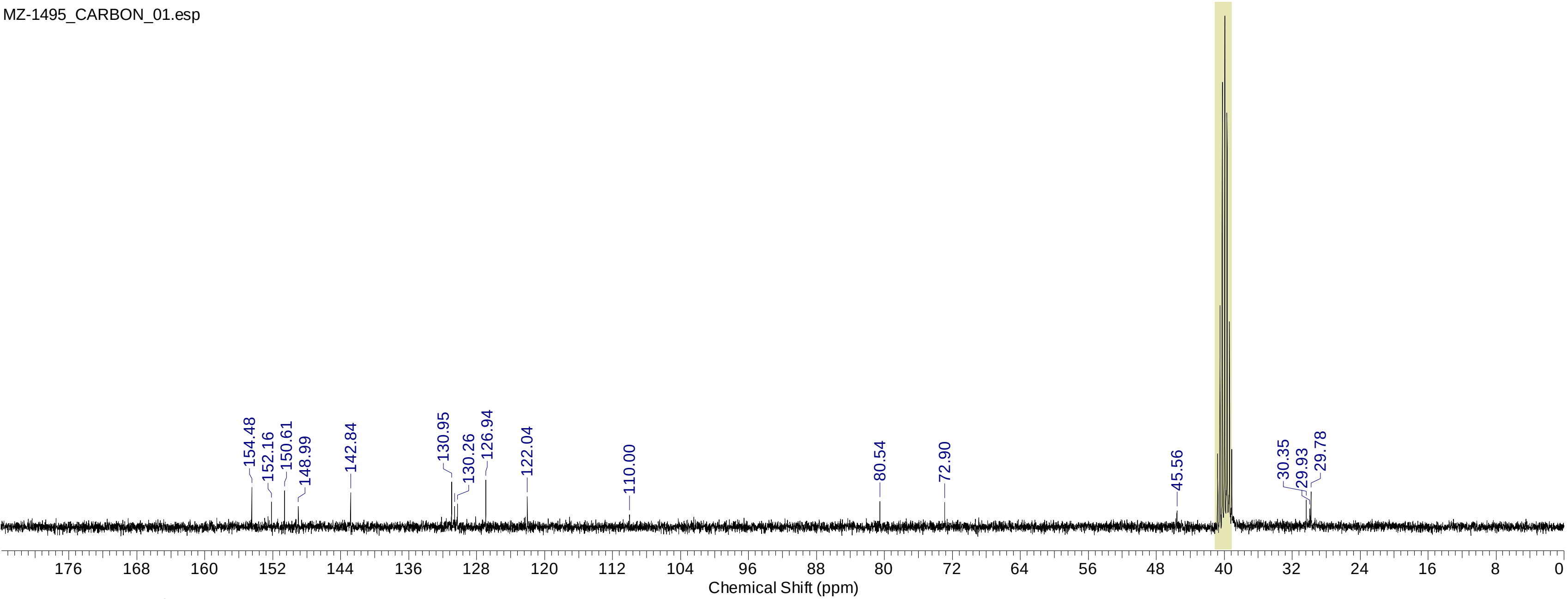

Figure 40S. ^{13}C NMR spectrum of 8-((3-bromobenzyl)amino)-3,7-dimethyl-1-(prop-2-yn-1-yl)-3,7-dihydro-1H-purine-2,6-dione (**24**)

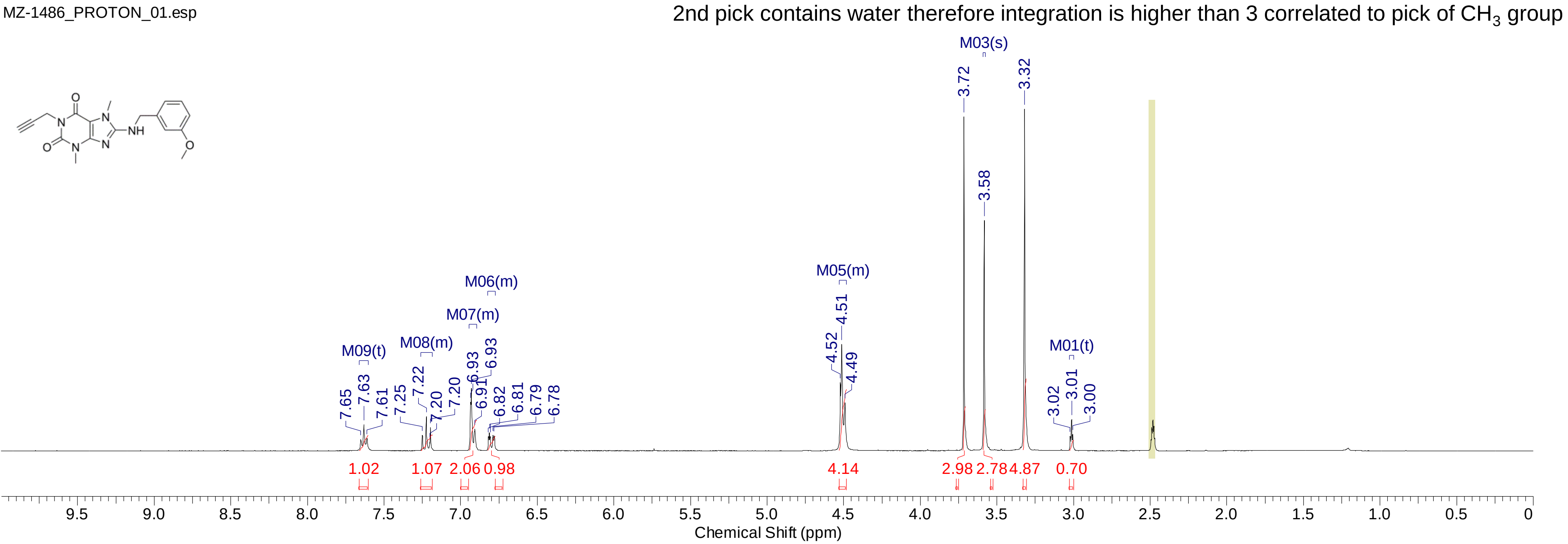

Figure 41S. ^{1}H NMR spectrum of 8-((3-methoxybenzyl)amino)-3,7-dimethyl-1-(prop-2-yn-1-yl)-3,7-dihydro-1H-purine-2,6-dione (**25**)

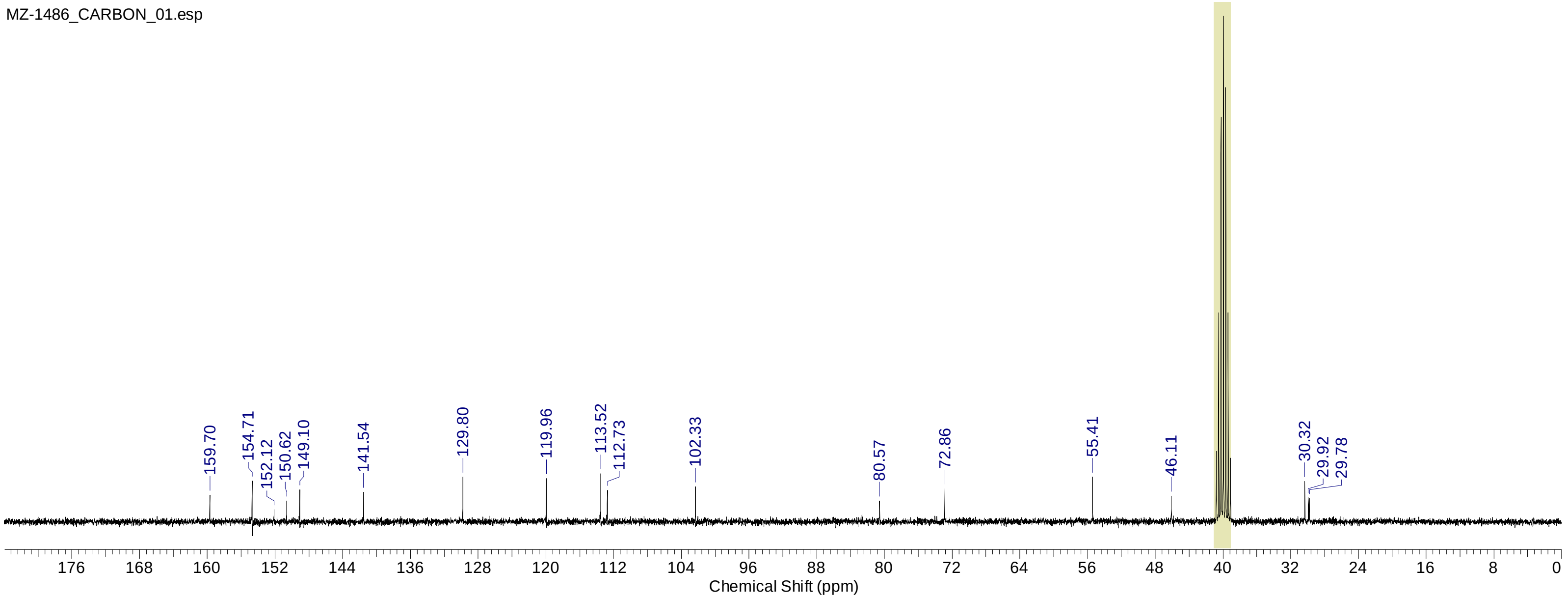

Figure 42S. ^{13}C NMR spectrum of 8-((3-methoxybenzyl)amino)-3,7-dimethyl-1-(prop-2-yn-1-yl)-3,7-dihydro-1H-purine-2,6-dione (**25**)

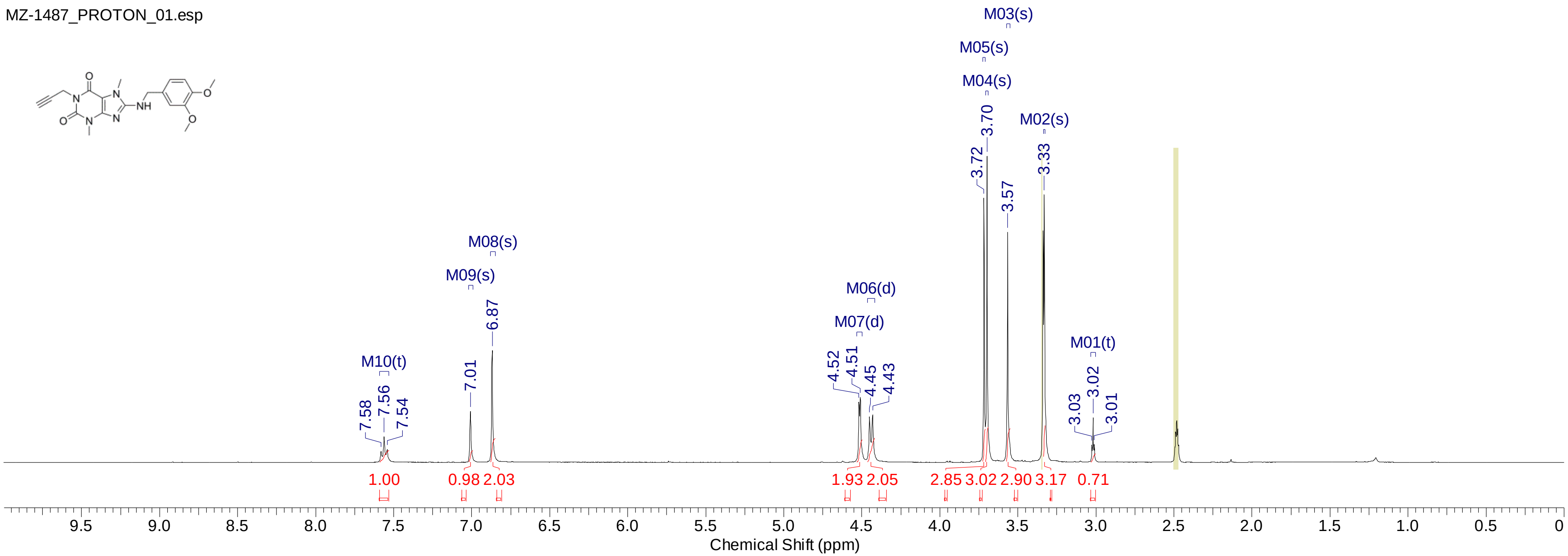

Figure 43S. ^{1}H NMR spectrum of 8-((3,4-dimethoxybenzyl)amino)-3,7-dimethyl-1-(prop-2-yn-1-yl)-3,7-dihydro-1H-purine-2,6-dione (**26**)

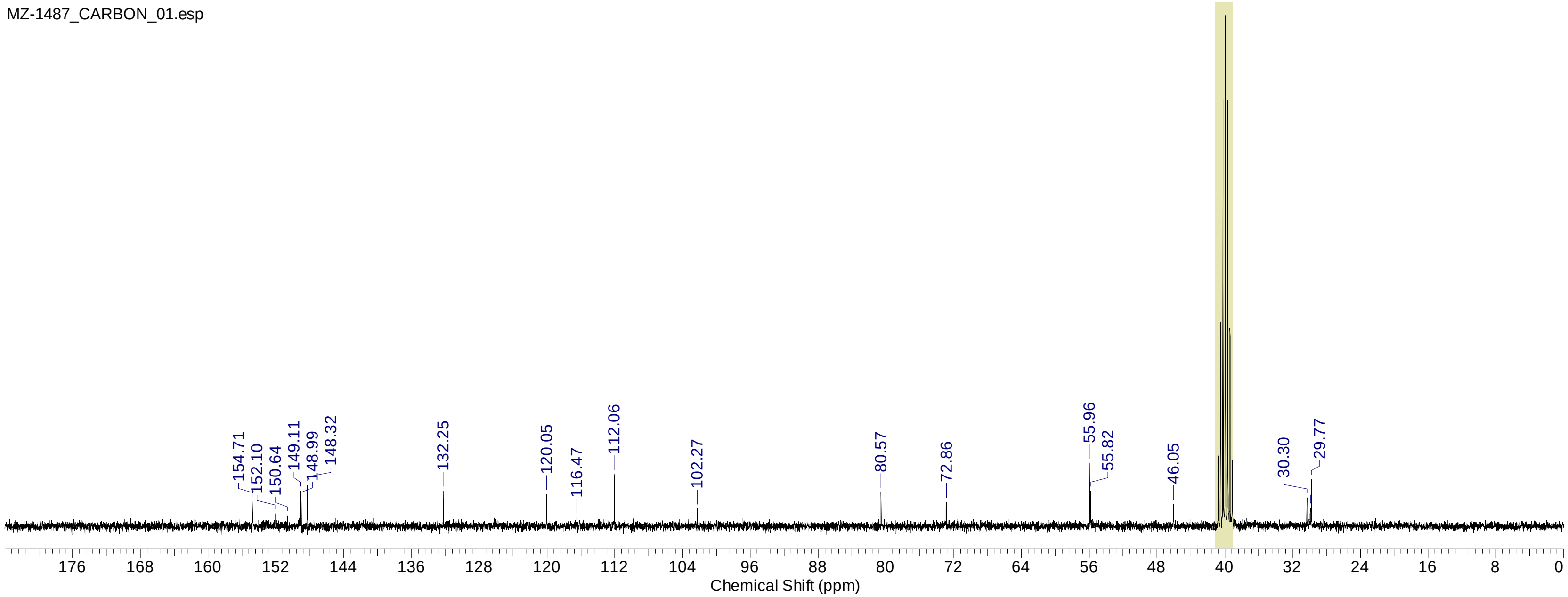

Figure 44S. ^{1}H NMR spectrum of 8-((3,4-dimethoxybenzyl)amino)-3,7-dimethyl-1-(prop-2-yn-1-yl)-3,7-dihydro-1H-purine-2,6-dione (**26**)

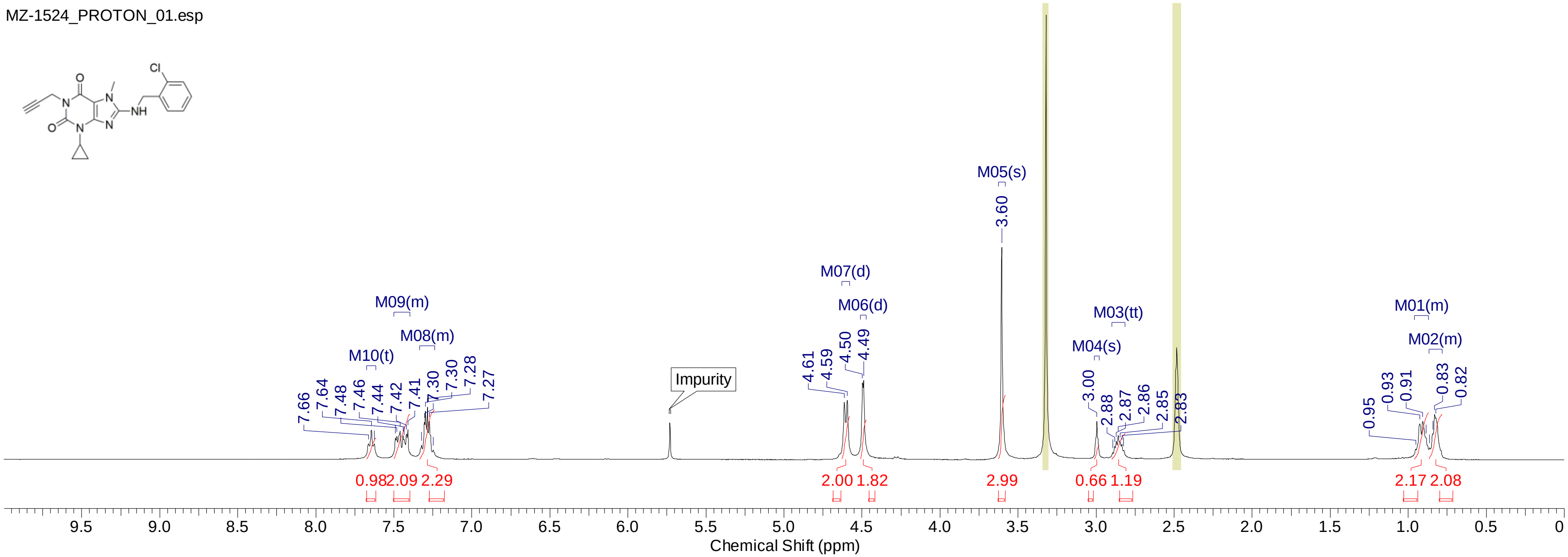

Figure 45S. ^{1}H NMR spectrum of 8-((2-chlorobenzyl)amino)-3-cyclopropyl-7-methyl-1-(prop-2-yn-1-yl)-3,7-dihydro-1H-purine-2,6-dione (**27**)

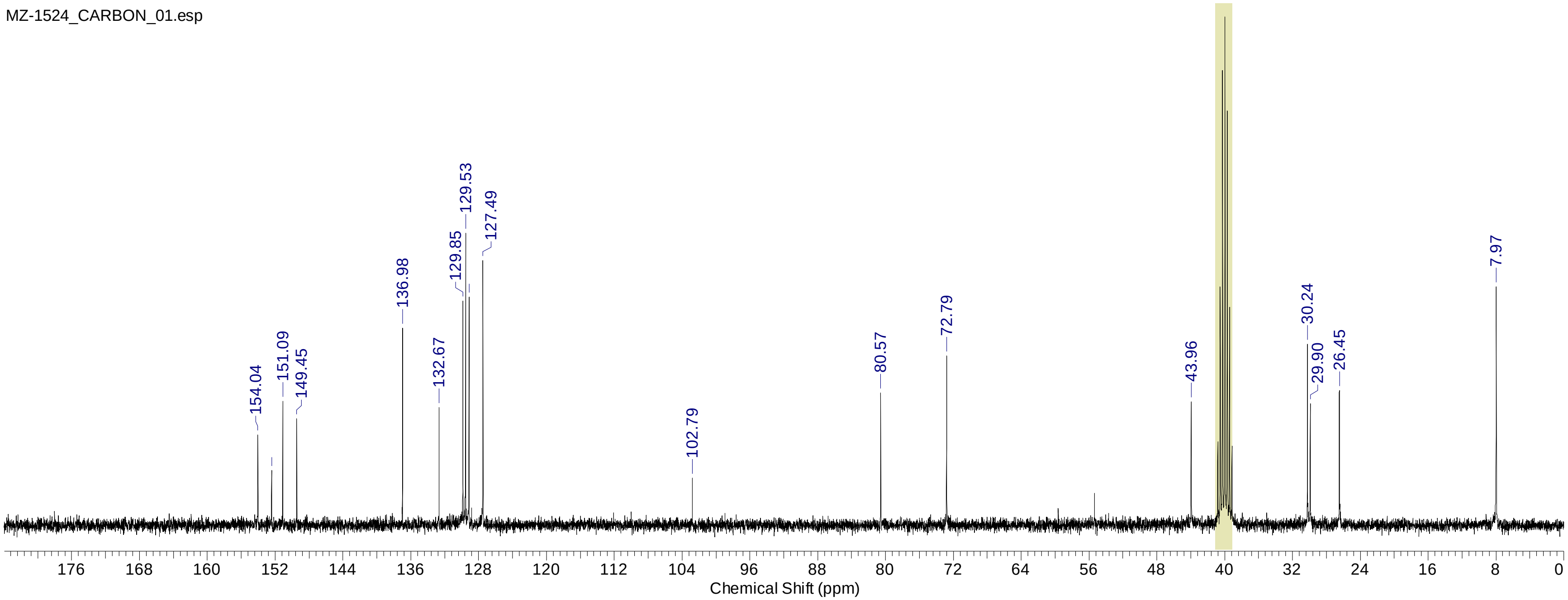

Figure 46S. ^{13}C NMR spectrum of 8-((2-chlorobenzyl)amino)-3-cyclopropyl-7-methyl-1-(prop-2-yn-1-yl)-3,7-dihydro-1H-purine-2,6-dione (**27**)

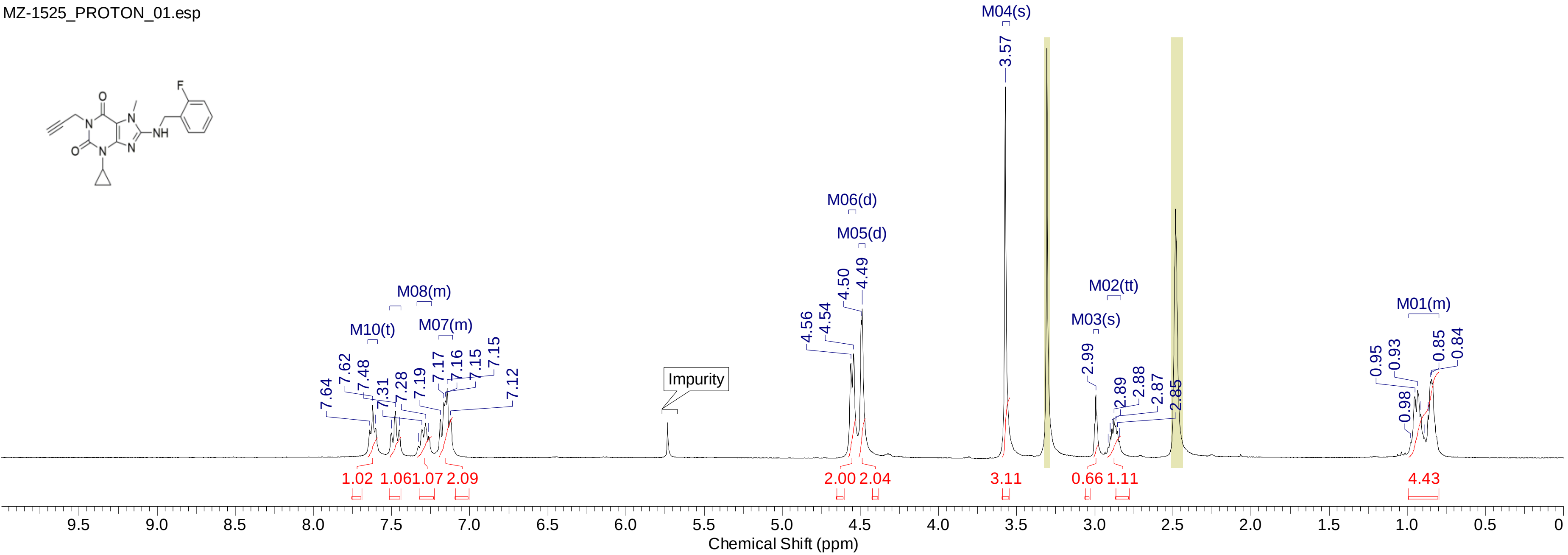

Figure 47S. ¹H NMR spectrum of 3-cyclopropyl-8-((2-fluorobenzyl)amino)-7-methyl-1-(prop-2-yn-1-yl)-3,7-dihydro-1H-purine-2,6-dione (**28**)

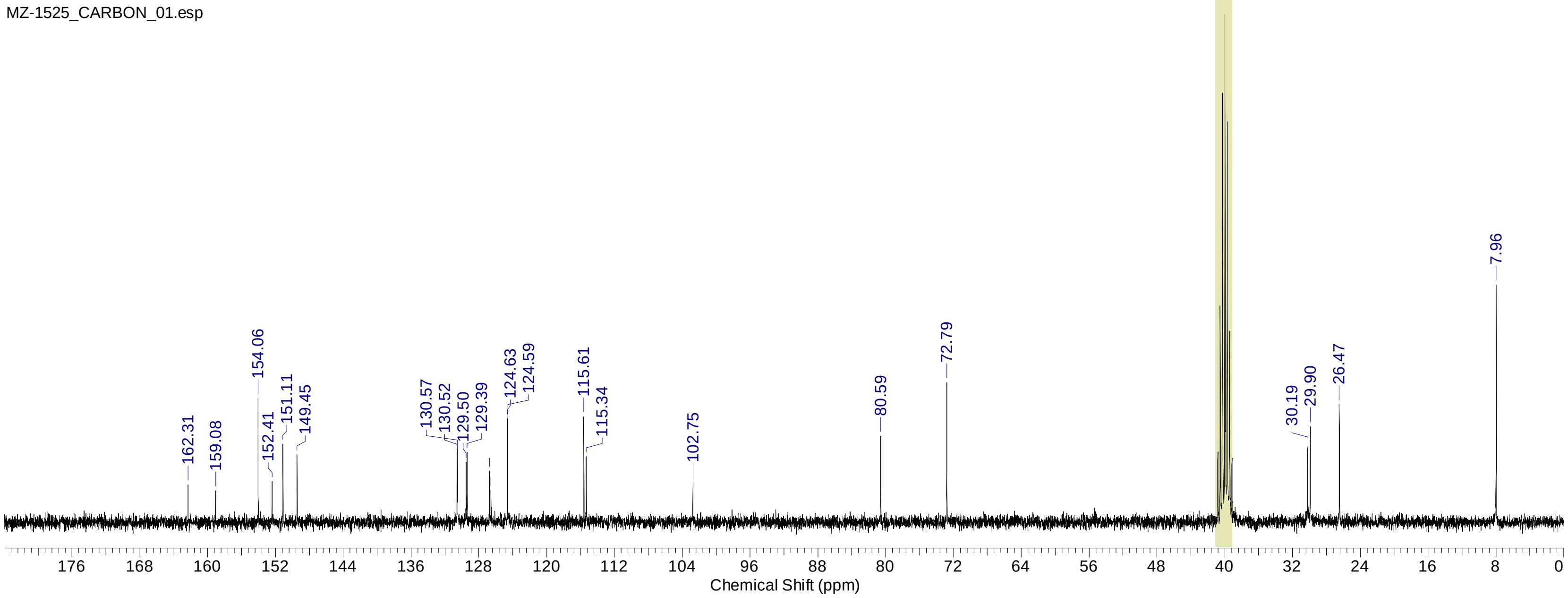

Figure 48S. ¹³C NMR spectrum of 3-cyclopropyl-8-((2-fluorobenzyl)amino)-7-methyl-1-(prop-2-yn-1-yl)-3,7-dihydro-1H-purine-2,6-dione (**28**)

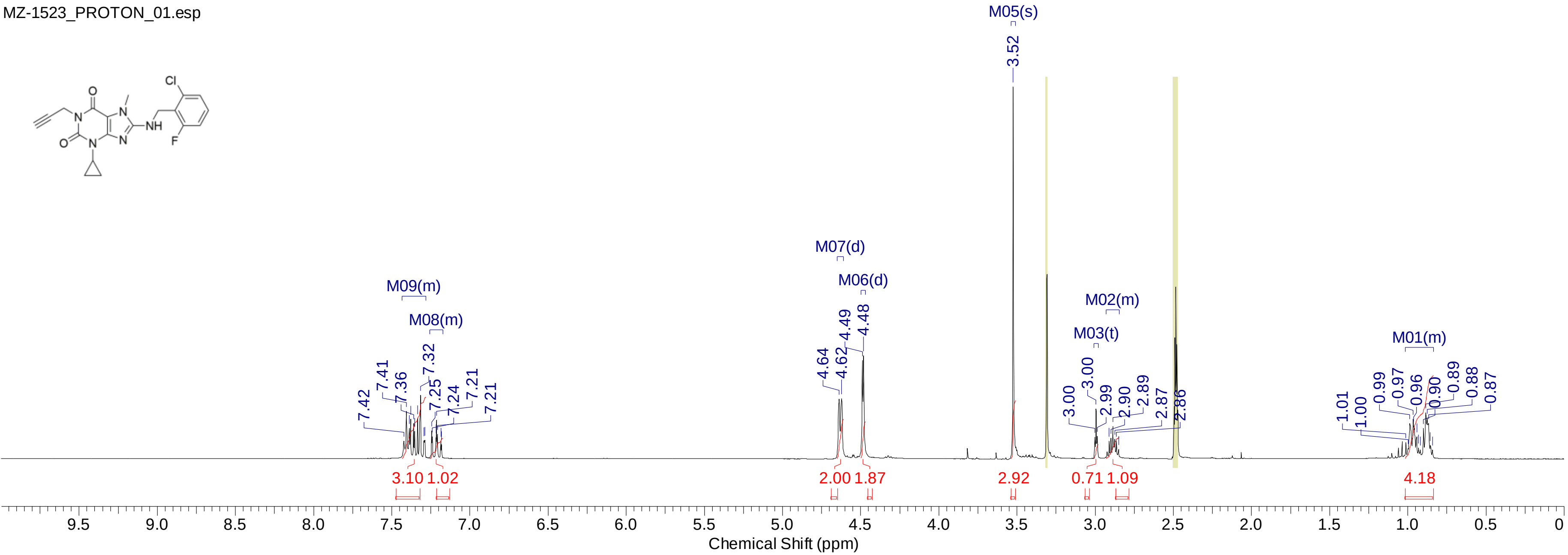

Figure 49S. ^{1}H NMR spectrum of 8-((2-chloro-6-fluorobenzyl)amino)-3-cyclopropyl-7-methyl-1-(prop-2-yn-1-yl)-3,7-dihydro-1H-purine-2,6-dione (**29**)

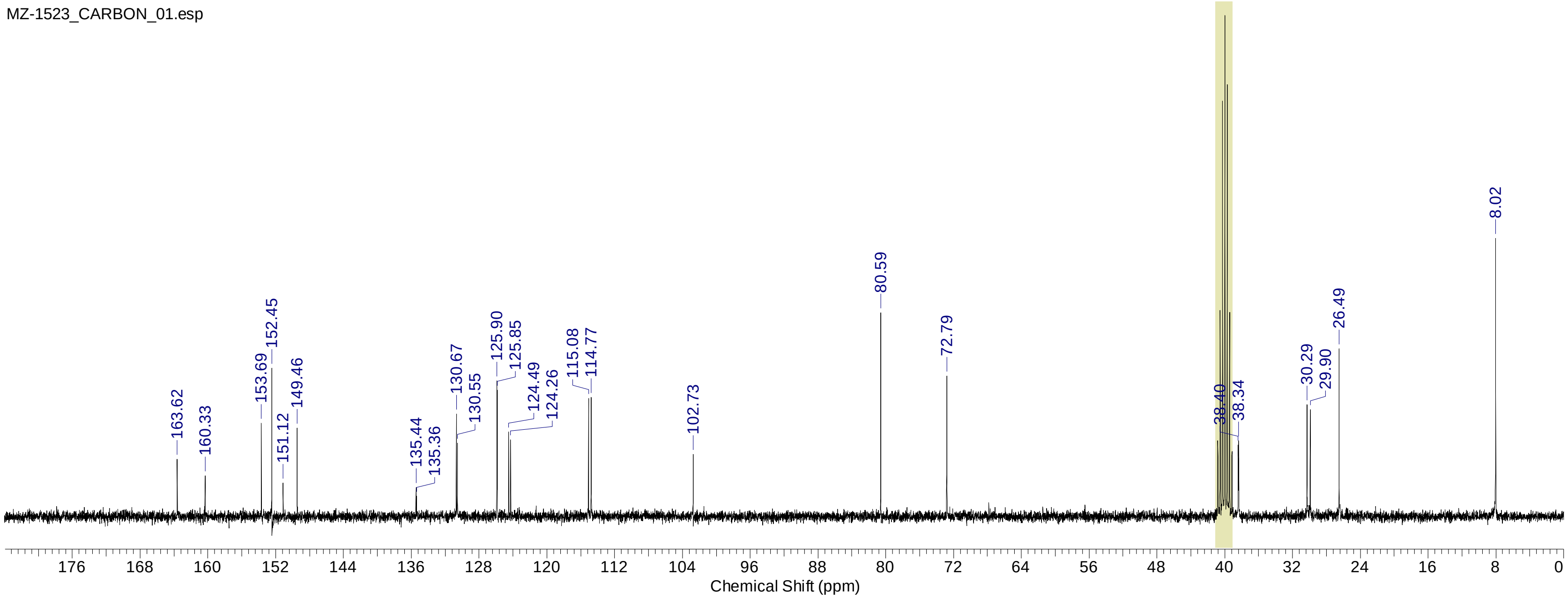

Figure 50S. ^{13}C NMR spectrum of 8-((2-chloro-6-fluorobenzyl)amino)-3-cyclopropyl-7-methyl-1-(prop-2-yn-1-yl)-3,7-dihydro-1H-purine-2,6-dione (**29**)
