# Supplementary material for: Anti-Inflammatory Activities of 8-Benzylaminoxanthines Showing High Adenosine A2A and Dual A1/A2A Receptor Affinity
Source: Int J Mol Sci. 2023 Sep 5;24(18):13707. doi: 10.3390/ijms241813707 (PMC10531311; doi:10.3390/ijms241813707)
Supplement: Supplementary file 1 [file ijms-24-13707-s001.zip › Metabolic stability -Figures S5-S12.pdf]

Metabolic stability studies of selected compounds

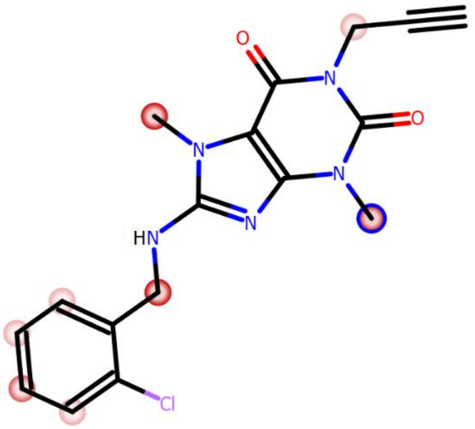

MZ1483 (compound **19**)

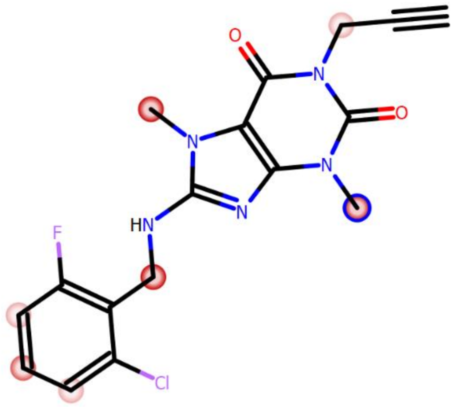

MZ1490 (compound **22**)

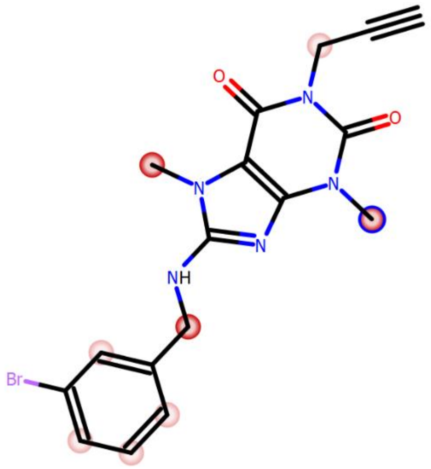

MZ1495 (compound **24**)

Figure S5. The MetaSite 6.0.1. software prediction of the most probable sites of metabolism. The darker red color - the higher probability to be involved in the metabolism pathway. The blue circle marked the site of compound with the highest probability of metabolic bioconversion**.**

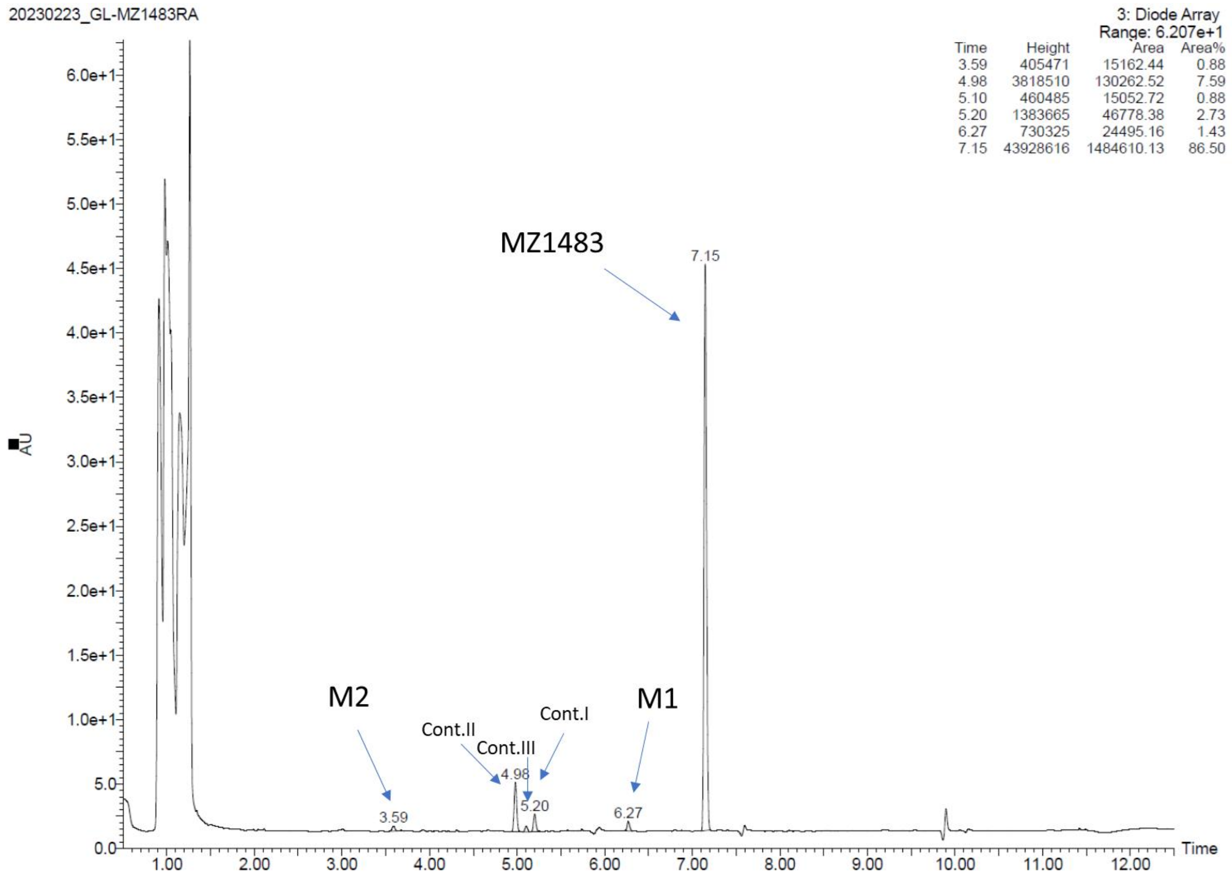

Figure S6. UPLC spectra after 120 min incubation of compound **MZ1483** with human liver microsomes (HLMs) in TRIS buffer pH=7.4 at 37ºC. Two metabolites were found. Three contaminations were identified.

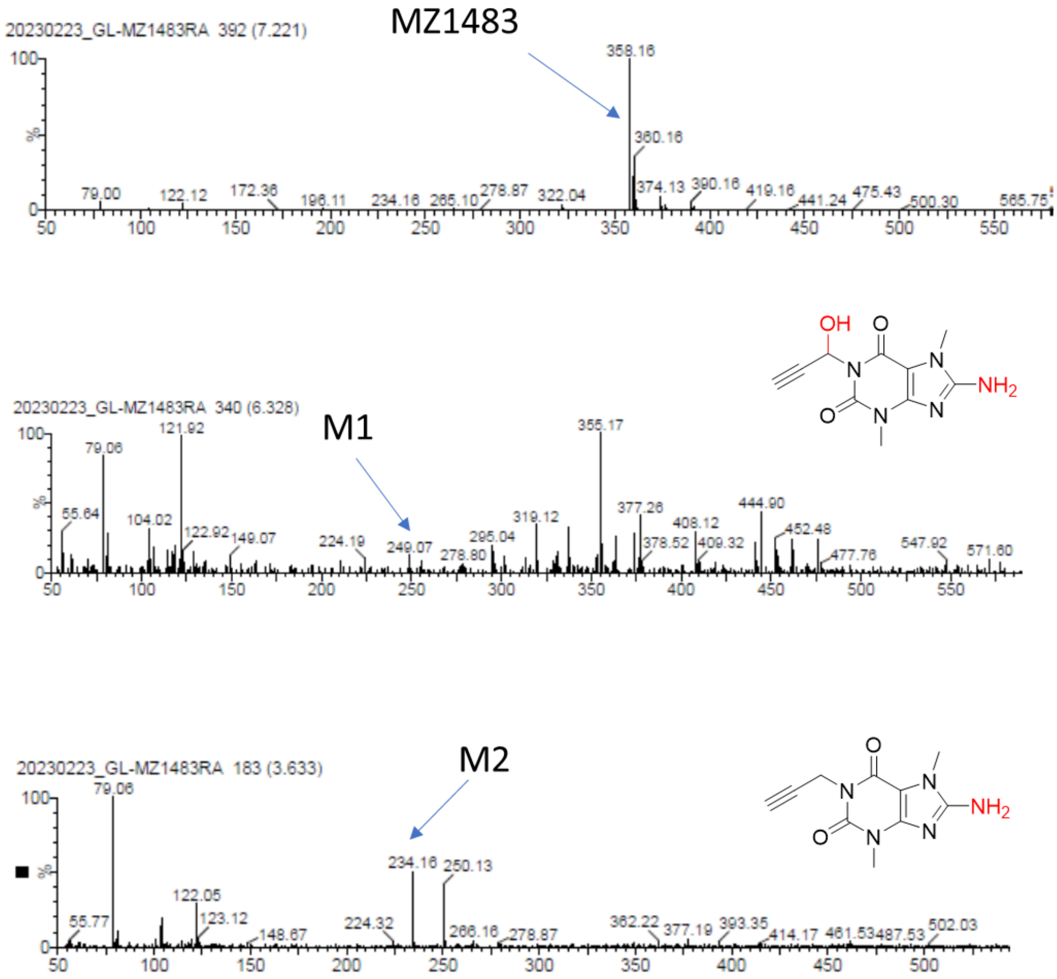

Figure S7. MS analyses of **MZ1483** and its metabolites M1 and M2. The most probable structures of metabolites were proposed based on MS analyses and the data obtained *in silico* (**Figure S5**).

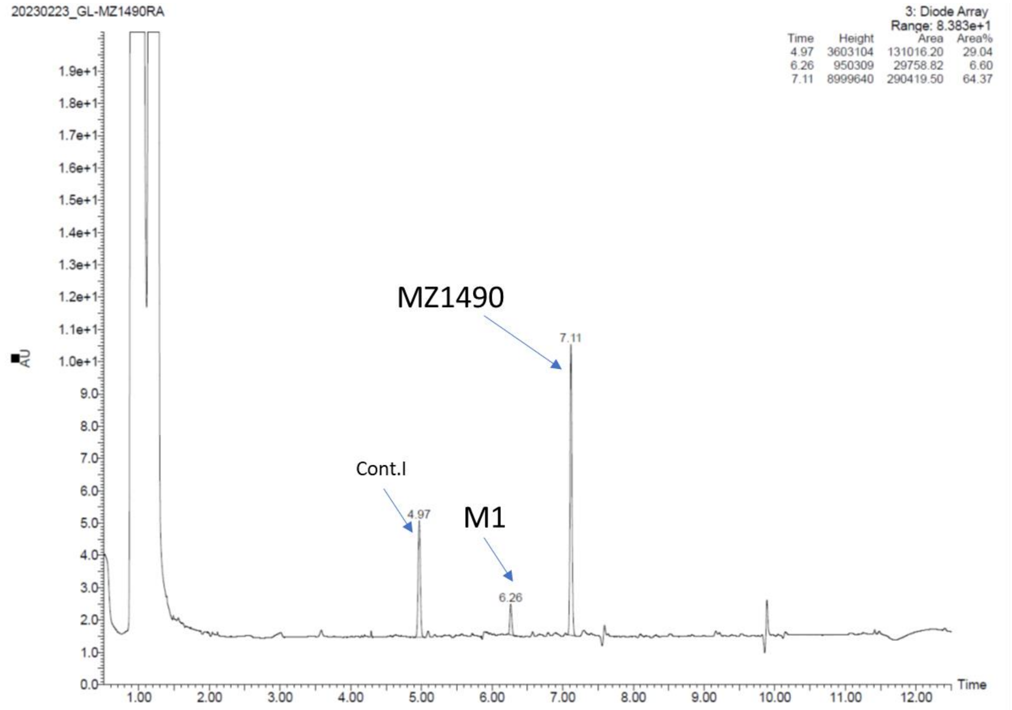

Figure S8. UPLC spectra after 120 min incubation of compound **MZ1490** with human liver microsomes (HLMs) in TRIS buffer pH = 7.4 at 37 ºC. One metabolite was found. One contamination was identified.

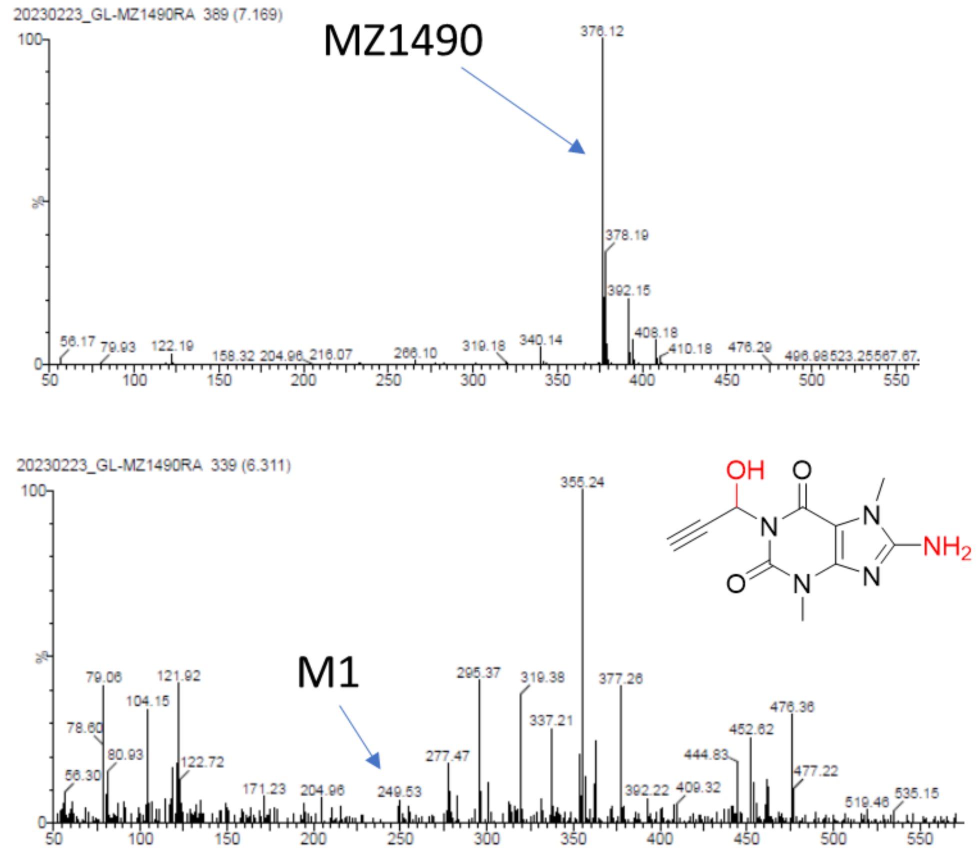

Figure S9. MS analyses of **MZ1490** and its metabolite M1. The most probable structure of metabolite was proposed based on MS analyses and the data obtained *in silico* (**Figure S5**).

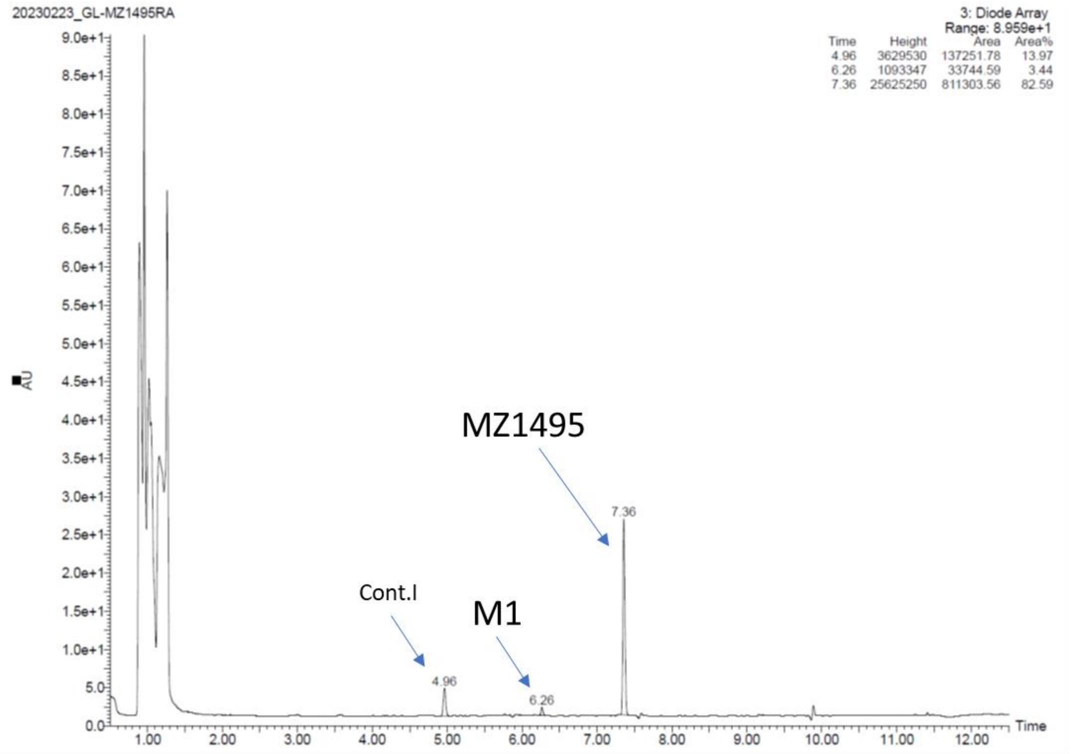

Figure S10. UPLC spectra after 120 min incubation of compound **MZ1495** with human liver microsomes (HLMs) in TRIS buffer pH = 7.4 at 37 ºC. One metabolite was found. One contamination was identified.

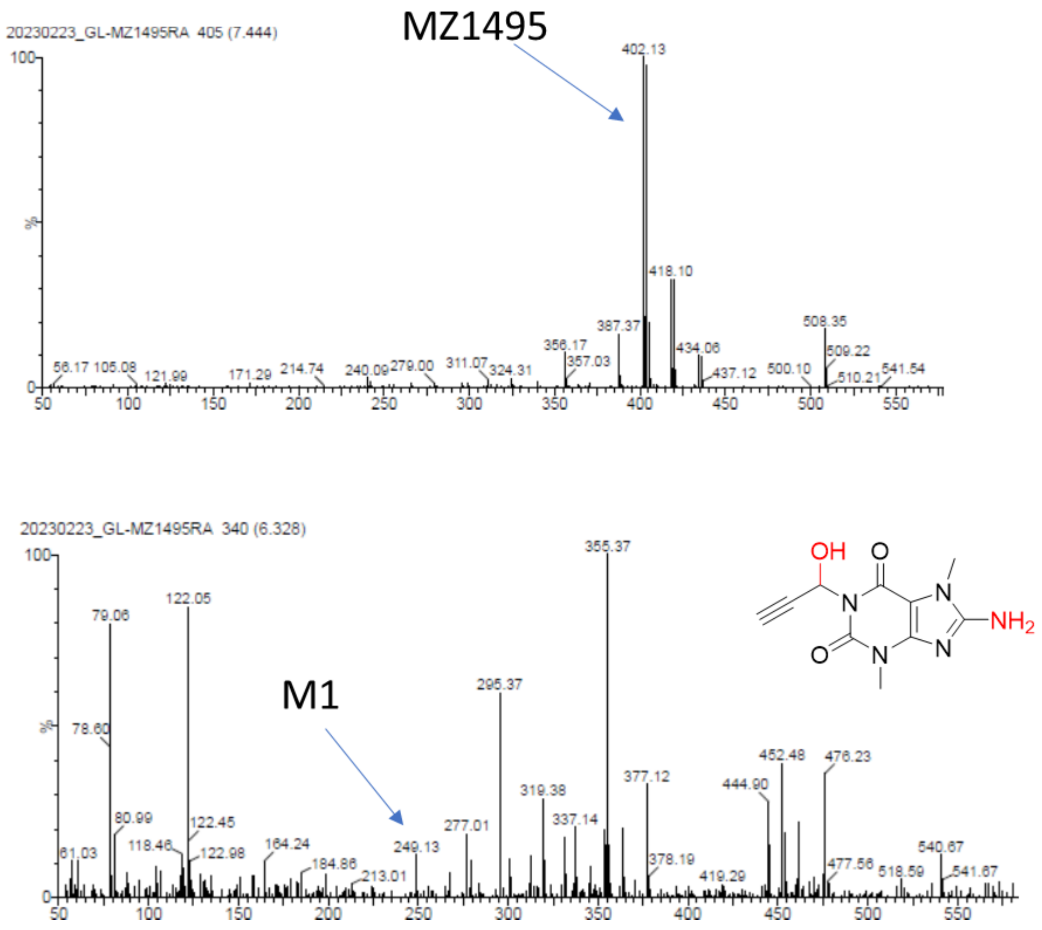

Figure S11. MS analyses of **MZ1495** and its metabolite M1. The most probable structure of metabolite was proposed based on MS analyses and the data obtained *in silico* (**Figure S5**).

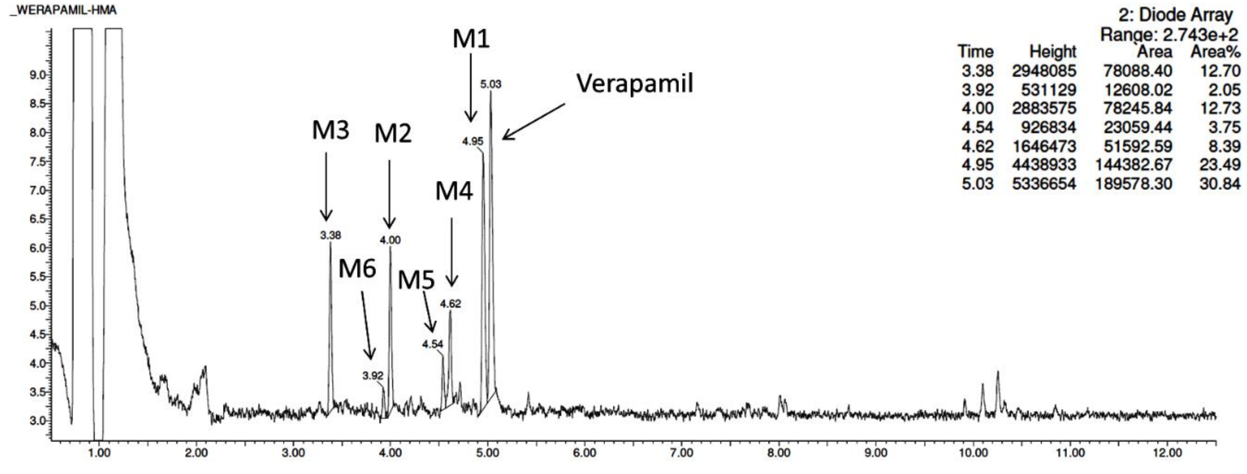

Figure S12. UPLC spectra after 120 min incubation of reference unstable drug **Verapamil** with human liver microsomes (HLMs) in TRIS buffer pH = 7.4 at 37 ºC.
