# Supplementary material for: Anti-Inflammatory Activities of 8-Benzylaminoxanthines Showing High Adenosine A2A and Dual A1/A2A Receptor Affinity
Source: Int J Mol Sci. 2023 Sep 5;24(18):13707. doi: 10.3390/ijms241813707 (PMC10531311; doi:10.3390/ijms241813707)
Supplement: Supplementary file 1 [file ijms-24-13707-s001.zip › Molecular docking -Figures S1-S4.pdf]

Molecular docking studies – Figures S1-S4

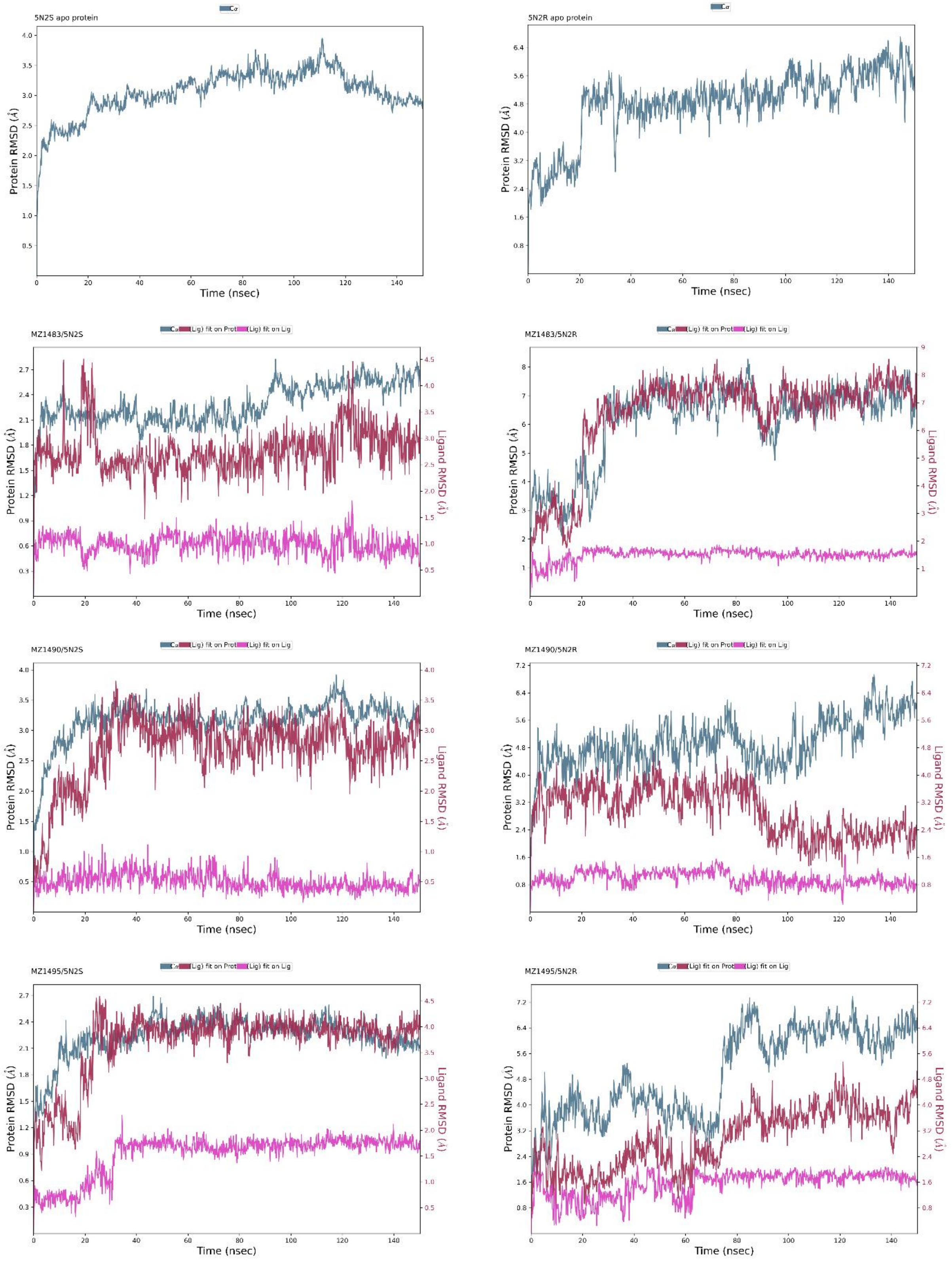

Figure S1 – Plots showing the RMSD evolution of 6 complexes and corresponding apo proteins (each protein structure used for each ligand) of a protein (left Y-axis) and ligand (right Y-axis). All protein frames are first aligned on the reference frame backbone, and then the RMSD is calculated based on the atom selection. Pink – Ligand fit on ligand (1st frame), Grayblue – Ca, maroon – Ligand fit on protein.

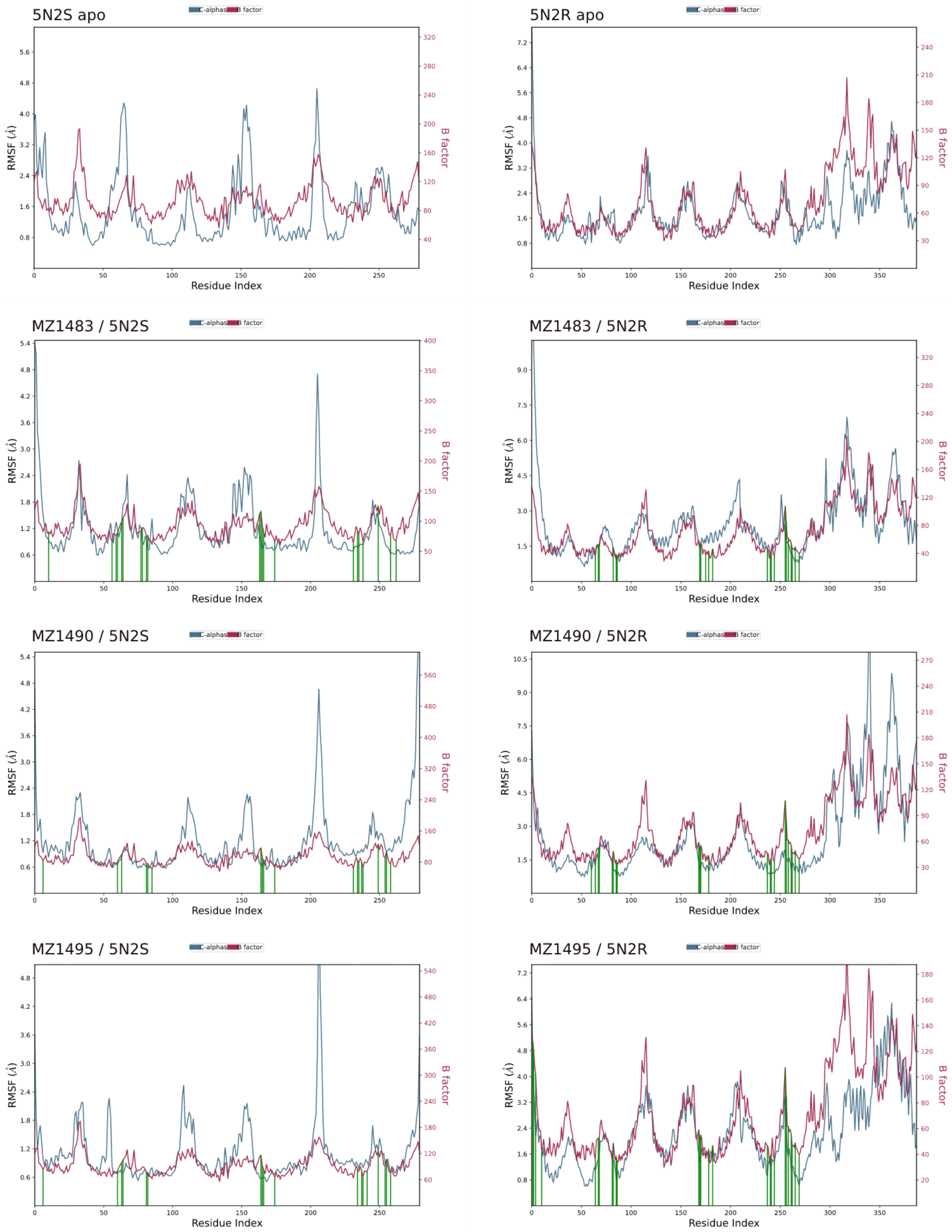

Figure S2 – Plots showing the RMSF 6 complexes and corresponding apo proteins (each protein structure used for each ligand), colored grey. Peaks indicate areas of the protein that fluctuate the most during the simulation. Protein residues that interact with the ligand are marked with green-colored vertical bars. The experimental x-ray B-factor (right Y-axis) is showed in magenta.

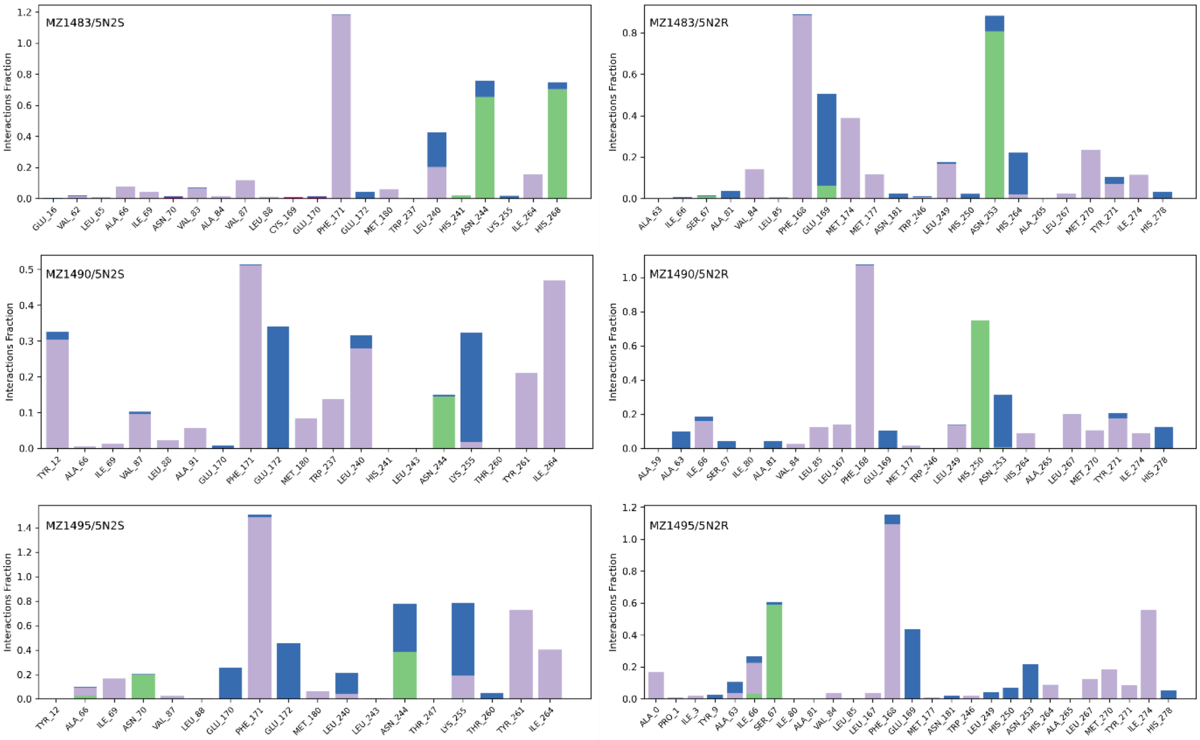

Figure S3 –Histograms of Protein -ligand interactions monitored throughout the simulation for 6 simulated complexes (each protein structure used for each ligand). Green – H-bonds, Violet – hydrophobic interactions, Pink – Ionic interactions, Blue – water bridges.

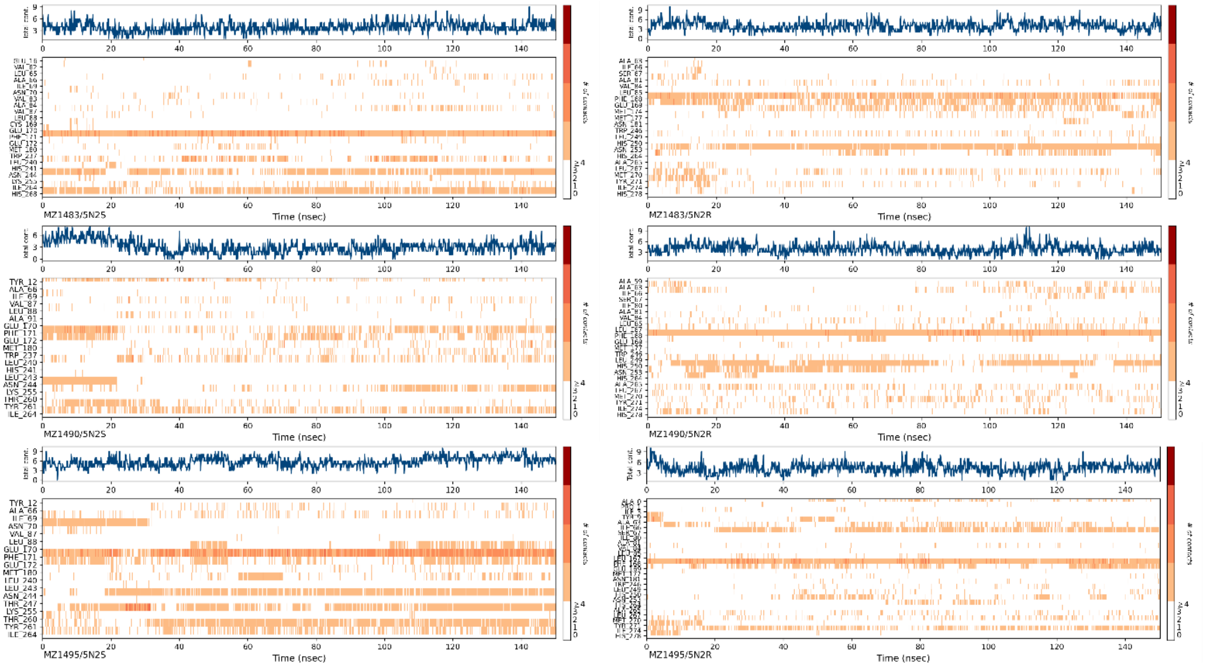

Figure S4 – Protein-ligand contacts timeline for 6 simulated complexes. The top panels (blue) shows the total number of specific contacts the protein makes with the ligand over the course of the trajectory. The bottom panels shows which residues interact with the ligand in each trajectory frame. Some residues make more than one specific contact with the ligand, which is represented by a darker shade of orange, according to the scale to the right of the plot.
