# Supplementary material for: Anti-Inflammatory Activities of 8-Benzylaminoxanthines Showing High Adenosine A2A and Dual A1/A2A Receptor Affinity
Source: Int J Mol Sci. 2023 Sep 5;24(18):13707. doi: 10.3390/ijms241813707 (PMC10531311; doi:10.3390/ijms241813707)
Supplement: Supplementary file 1 [file ijms-24-13707-s001.zip › Molecular docking -Table S1.pdf]

Table S1 SMILES notation for the ligands obtained within the presented study

Ligand	SMILES notation
2	ClC1=CC=CC=C1CNC(N2C)=NC3=C2C(N(CC)C(N3C)=O)=O
5	O=C1C2=C(N=C(NCC3=C(F)C=CC=C3Cl)N2C)N(C)C(N1CCC)=O
6	ClC1=CC=CC=C1CNC(N2C)=NC3=C2C(N(CCCC)C(N3C)=O)=O
7	ClC1=CC=CC=C1CNC(N2C)=NC3=C2C(N(CCCCC)C(N3C)=O)=O
8	ClC1=CC=CC=C1CNC(N2C)=NC3=C2C(N(CCCCCC)C(N3C)=O)=O
9	ClC1=CC=CC=C1CNC(N2C)=NC3=C2C(N(CC4=CC=CC=C4)C(N3C)=O)=O
10	ClC1=CC=CC=C1CNC(N2C)=NC3=C2C(N(CC4=CC=C(Cl)C=C4)C(N3C)=O)=O
11	ClC1=CC=CC=C1CNC(N2C)=NC3=C2C(N(CC4=CC(Cl)=C(Cl)C=C4)C(N3C)=O)=O
12	O=C1C2=C(N=C(NCC3=CC=CC=C3Br)N2C)N(C)C(N1CCC)=O
13	O=C1C2=C(N=C(NCC3=CC=CC=C3F)N2C)N(C)C(N1CCC)=O
14	O=C1C2=C(N=C(NCC3=C(F)C=CC=C3Cl)N2C)N(C)C(N1CCC)=O
15	O=C1C2=C(N=C(NCC3=CC=CC(Cl)=C3)N2C)N(C)C(N1CCC)=O
16	O=C1C2=C(N=C(NCC3=CC=CC(Br)=C3)N2C)N(C)C(N1CCC)=O
17	O=C1C2=C(N=C(NCC3=CC=CC(OC)=C3)N2C)N(C)C(N1CCC)=O
18	O=C1C2=C(N=C(NCC3=CC=C(OC)C(OC)=C3)N2C)N(C)C(N1CCC)=O
19	O=C1C2=C(N=C(NCC3=CC=CC=C3Cl)N2C)N(C)C(N1CC#C)=O
20	O=C1C2=C(N=C(NCC3=CC=CC=C3Br)N2C)N(C)C(N1CC#C)=O
21	O=C1C2=C(N=C(NCC3=CC=CC=C3F)N2C)N(C)C(N1CC#C)=O
22	O=C1C2=C(N=C(NCC3=C(F)C=CC=C3Cl)N2C)N(C)C(N1CC#C)=O
23	O=C1C2=C(N=C(NCC3=CC=CC(Cl)=C3)N2C)N(C)C(N1CC#C)=O
24	O=C1C2=C(N=C(NCC3=CC=CC(Br)=C3)N2C)N(C)C(N1CC#C)=O
25	O=C1C2=C(N=C(NCC3=CC=CC(OC)=C3)N2C)N(C)C(N1CC#C)=O
26	O=C1C2=C(N=C(NCC3=CC=C(OC)C(OC)=C3)N2C)N(C)C(N1CC#C)=O
27	O=C1C2=C(N=C(NCC3=CC=CC=C3Cl)N2C)N(C4CC4)C(N1CC#C)=O
28	O=C1C2=C(N=C(NCC3=CC=CC=C3F)N2C)N(C4CC4)C(N1CC#C)=O
29	O=C1C2=C(N=C(NCC3=C(F)C=CC=C3Cl)N2C)N(C4CC4)C(N1CC#C)=O
